# Supplementary material for: Trained immunity induced by high‐salt diet impedes stroke recovery
Source: EMBO Rep. 2023 Nov 15;24(12):e57164. doi: 10.15252/embr.202357164 (PMC10702837; doi:10.15252/embr.202357164)
Supplement: Supplementary file 1 — Appendix [file EMBR-24-e57164-s014.pdf]

**Trained immunity induced by high salt diet impedes stroke recovery**

Tze-Yen Lin, Danye Jiang, Wan-Ru Chen, Jhih Syuan Lin, Xin-Yu Zhang, Chih-Hung Chen, Chia-Lang Hsu, Liang-Chuan Lai, Ping-Hung Chen, Kai-Chien Yang, Lauren H Sansing, Che-Feng Chang*

*Correspondence to:

Che-Feng Chang, Department and Graduate Institute of Physiology, National Taiwan University College of Medicine, No. 1, Sec. 1, Jen-Ai Rd., Room 1038, Taipei 100, Taiwan.

Phone: +886-2-23123456 ext. 288238; E-mail: chefengchang@ntu.edu.tw

## Contents

**Appendix Figure S1.** Gating strategies to distinguish monocyte-derived macrophages (MDMs) and microglia in the ICH brain……………………………………………………………………2

**Appendix Figure S2.** Significantly enriched canonical pathways identified in HSD HSPCs…...3

**Appendix Figure S3.** The efficiency of NR4a1-expressing lentivirus and NR4a1 overexpression ……………………………………………………………………………….………....5

## Appendix Figure S1

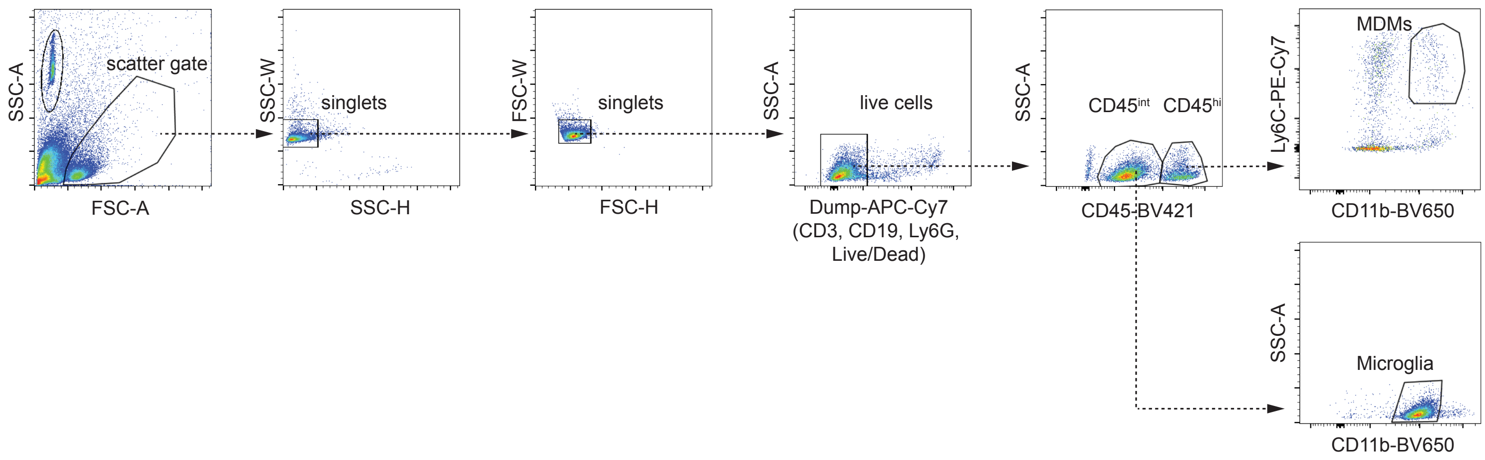


**Appendix Figure S1. Gating strategies to distinguish monocyte-derived macrophages (MDMs) and microglia in the ICH brain.** MDMs are identified as the $CD45^{hi}CD11b^{+}Ly6C^{+}$ population and microglia as the $CD45^{int}CD11b^{+}$ population.

## Appendix Figure S2

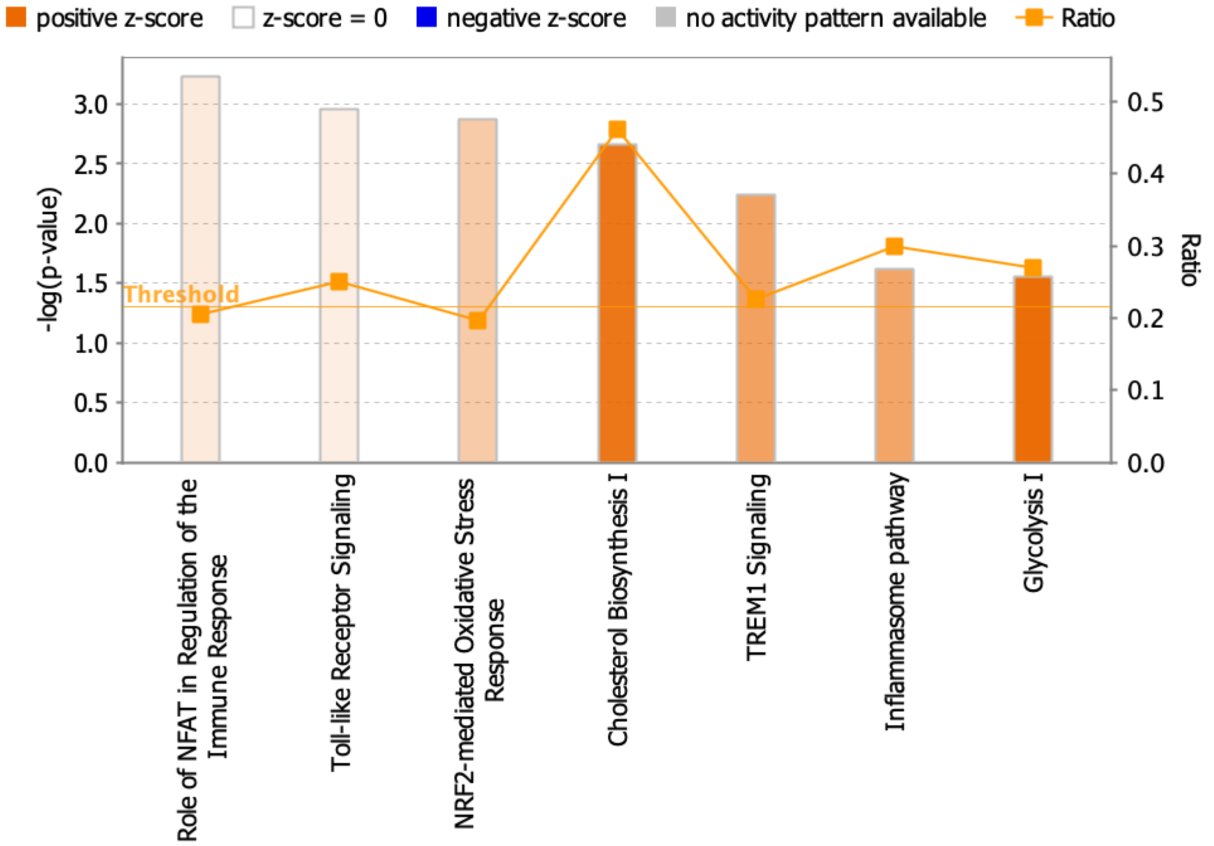


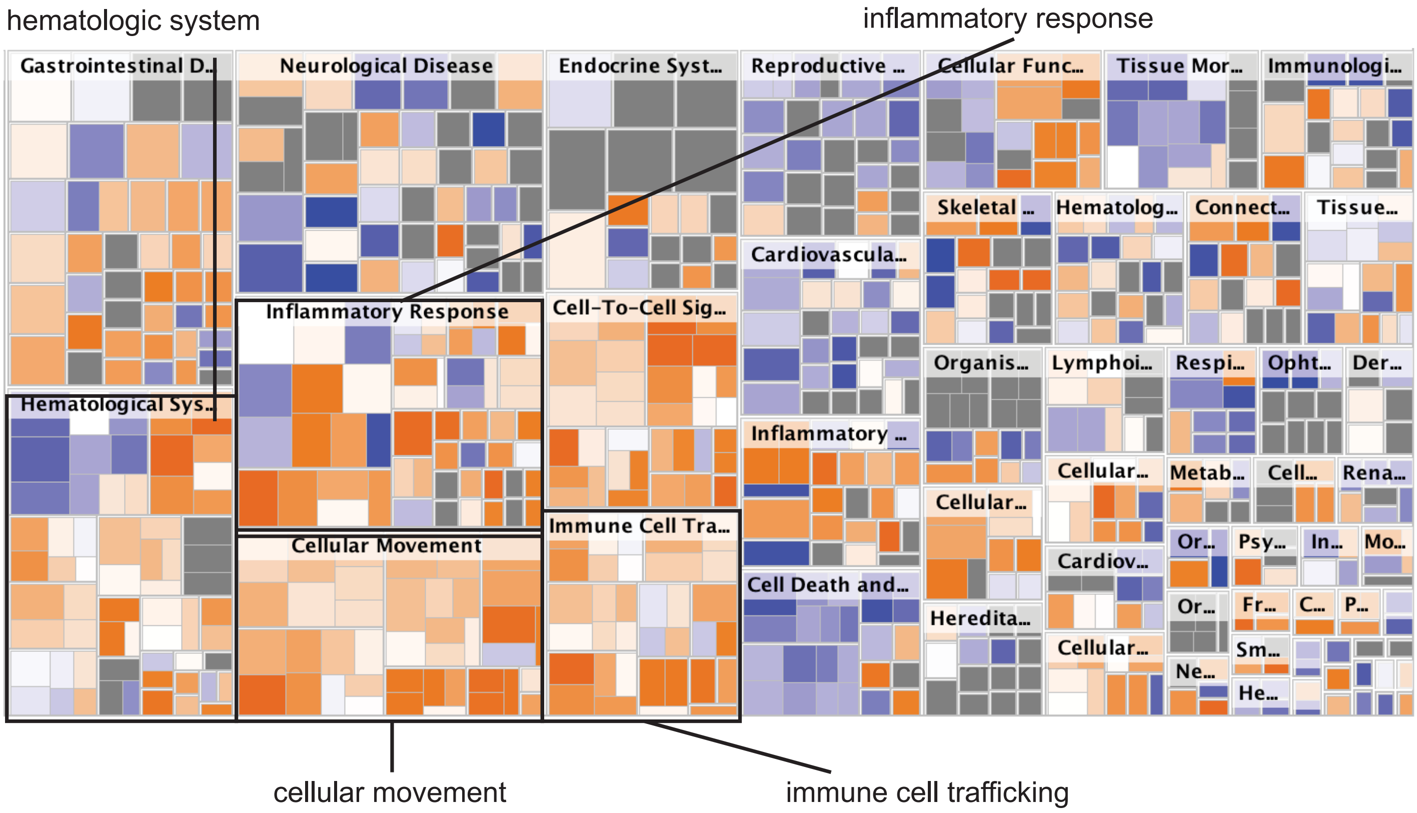

**Appendix Figure S2. Ingenuity pathway analysis (IPA) of significantly enriched canonical pathways identified in HSD HSPCs.** Top: The classical pathway enrichment analysis indicates pathway activation (orange, z-score >0). Bottom: Heat maps of disease and functional categories show the relationships between changes in differentially expressed gene levels and the activation and inhibition of diseases and functions. Orange indicates that the disease or functional status is activated (z-score >0), blue indicates that the disease or functional status is suppressed (z-score <0), and gray indicates that the disease or functional status is undetermined (z-score cannot be calculated).

## Appendix Figure S3

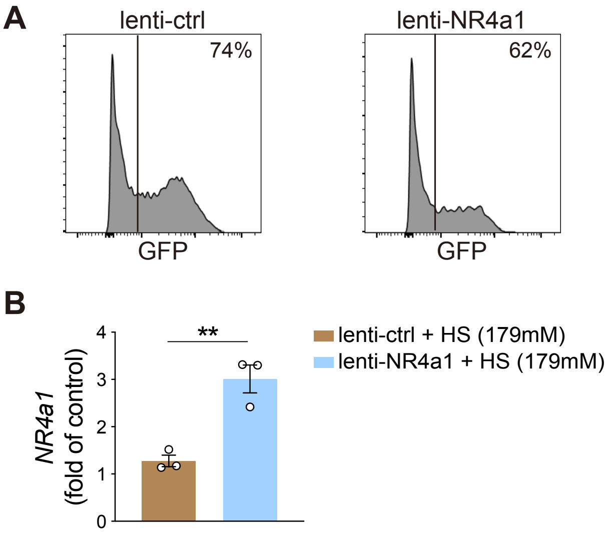


**Appendix Figure S3. The efficiency of NR4a1-expressing lentivirus and NR4a1 overexpression.** (**A**) Representative histograms showing transduction efficiency of GFP-tagged control lentiviral vector (lenti-ctrl) and NR4a1-expressing vector (lenti-NR4a1). The transduction efficiency in bone marrow cells was assessed by measuring the percentage of GFP+ cells. (**B**) Expression of NR4a1 in BMDMs after NR4a1-expressing lentivirus transduction and high-salt differentiation (lenti-NR4a1+HS) compared to those with control vector transduction and high-salt differentiation (lenti-ctrl+HS). Data are mean ± SEM, $n$=3/group, $^{**}P < 0.01$ (Student's $t$-test), $n$: biological replicates.
