# Supplementary material for: Trained immunity induced by high‐salt diet impedes stroke recovery
Source: EMBO Rep. 2023 Nov 15;24(12):e57164. doi: 10.15252/embr.202357164 (PMC10702837; doi:10.15252/embr.202357164)
Supplement: Supplementary file 2 — Expanded View Figures PDF [file EMBR-24-e57164-s002.pdf]

Expanded View Figures

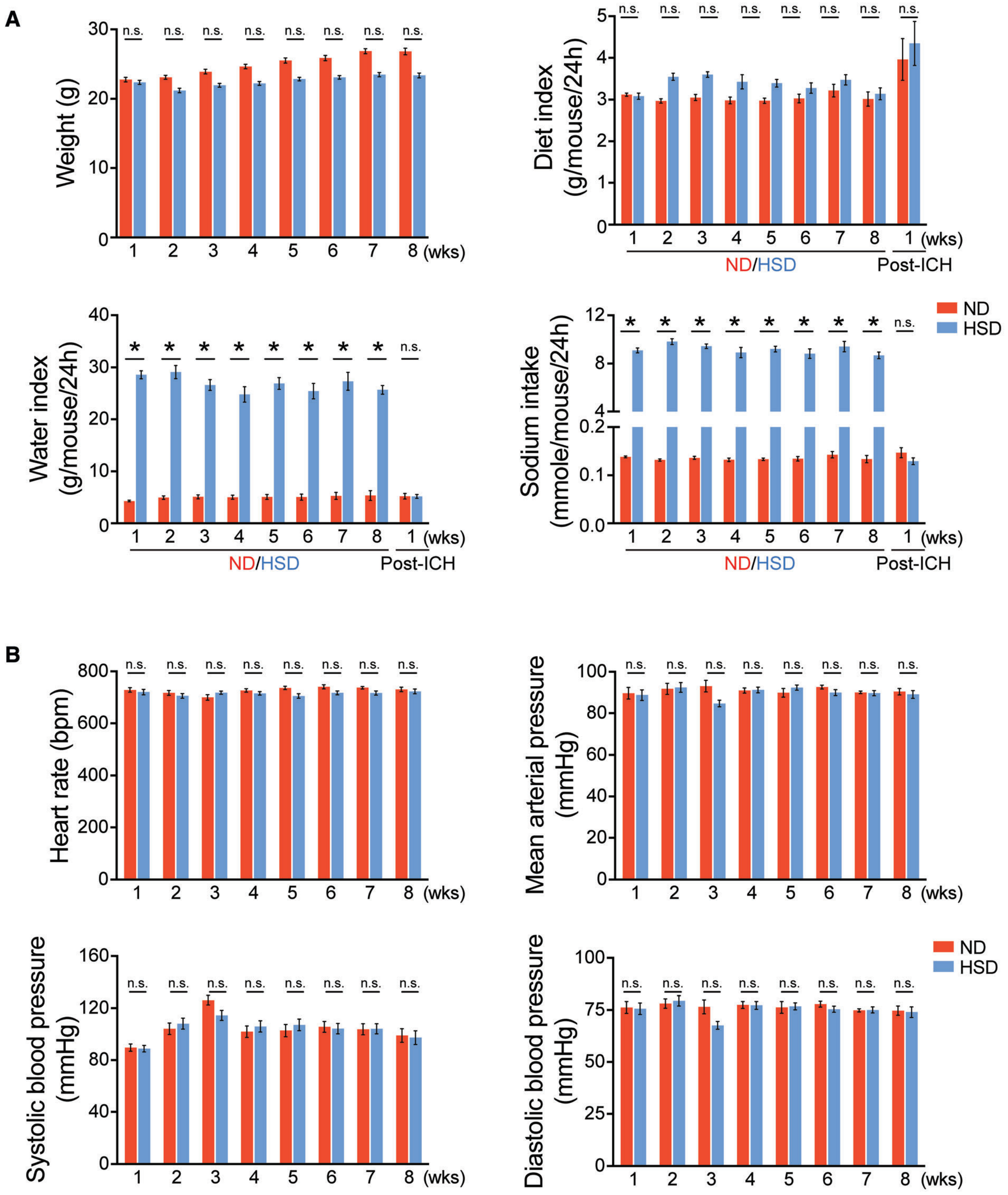

Figure EV1.

Figure EV1. Physiological measurements of ND and HSD mice.

A Graphs depicting body weight, food intake, water intake, and sodium intake in ND (*n* = 22–33) and HSD (*n* = 24–35) mice over 8 weeks of diet manipulation. Two-way ANOVA and Bonferroni test. *n*: biological replicates.

B Graphs depicting heart rate, mean arterial pressure, systolic blood pressure, and diastolic blood pressure in ND and HSD animals over 8 weeks of diet manipulation. *n* = 7–11/group, two-way ANOVA and Bonferroni test. *n*: biological replicates.

Data information: All data are mean ± SEM; $^*P < 0.05$, n.s., not significant.
Source data are available online for this figure.

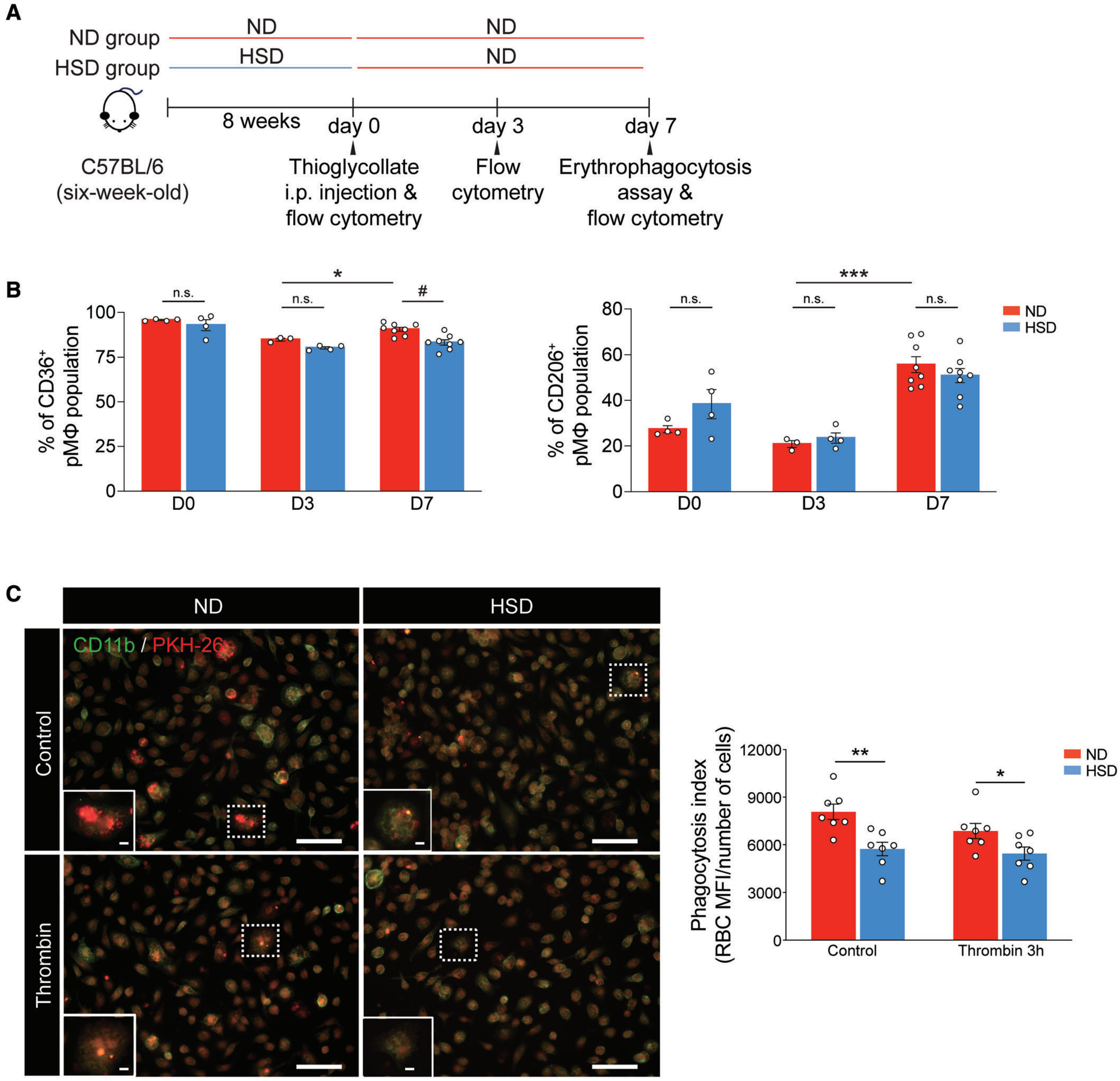

Figure EV2.

© 2023 The Authors

Figure EV2. HSD reduces alternative activation of peritoneal macrophages in a thioglycollate-elicited peritoneal model.

A Schematic of experimental design.
B Bar graphs depicting the percentages of $CD36^+$ and $CD206^+$ peritoneal macrophages from ND and HSD mice on days 0, 3, and 7 following thioglycollate injection. $n = 4$/group on day 0, $n = 3$ ND, 4 HSD on day 3, and $n = 8$/group on day 7, two-way ANOVA and Bonferroni test. *n*: biological replicates.
C Representative images showing engulfment of heat-shocked PKH26-labeled erythrocytes (red) in $CD11b^+$ (green) ND and HSD peritoneal macrophages with or without thrombin stimulation. Quantifications of erythrophagocytosis in ND and HSD peritoneal macrophages with or without thrombin stimulation. $n = 7$/group, Student's *t*-test. *n*: biological replicates. Scale bar: 50 μm and 5 μm high-magnification insets.

Data information: All data are mean ± SEM, each symbol represents one mouse or one biological replicate, $*P < 0.05$, $**P < 0.01$, $***P < 0.001$, and $^{\#}P < 0.001$, n.s., not significant.
Source data are available online for this figure.

Figure EV3. HSD impairs mitochondrial oxidative phosphorylation and promotes glycolysis in bone marrow (BM) cells.

A OCR traces and maximum respiratory capacity of BM cells after 4-week ND and HSD. $n = 9$ ND, 6 HSD, Student's *t*-test. *n*: biological replicates.
B OCR traces and maximum respiratory capacity of BM cells after 8-week ND and HSD. $n = 9$ ND, 6 HSD, Student's *t*-test. *n*: biological replicates.
C ECAR traces and glycolytic capacity of BM cells after 4-week ND and HSD. $n = 5$/group, Student's *t*-test. *n*: biological replicates.
D ECAR traces and glycolytic capacity of BM cells after 8-week ND and HSD. $n = 8$/group, Student's *t*-test. *n*: biological replicates.

Data information: All data are mean ± SEM, each symbol represents one mouse, $*P < 0.05$, $**P < 0.01$, and $***P < 0.001$; OCR, oxygen consumption rate, OM, oligomycin, FCCP, carbonyl cyanide-4 (trifluoromethoxy) phenylhydrazone, ROT/AA, rotenone and antimycin A, ECAR, extracellular acidification rate, and 2-DG, 2-deoxy-D-glucose.
Source data are available online for this figure.

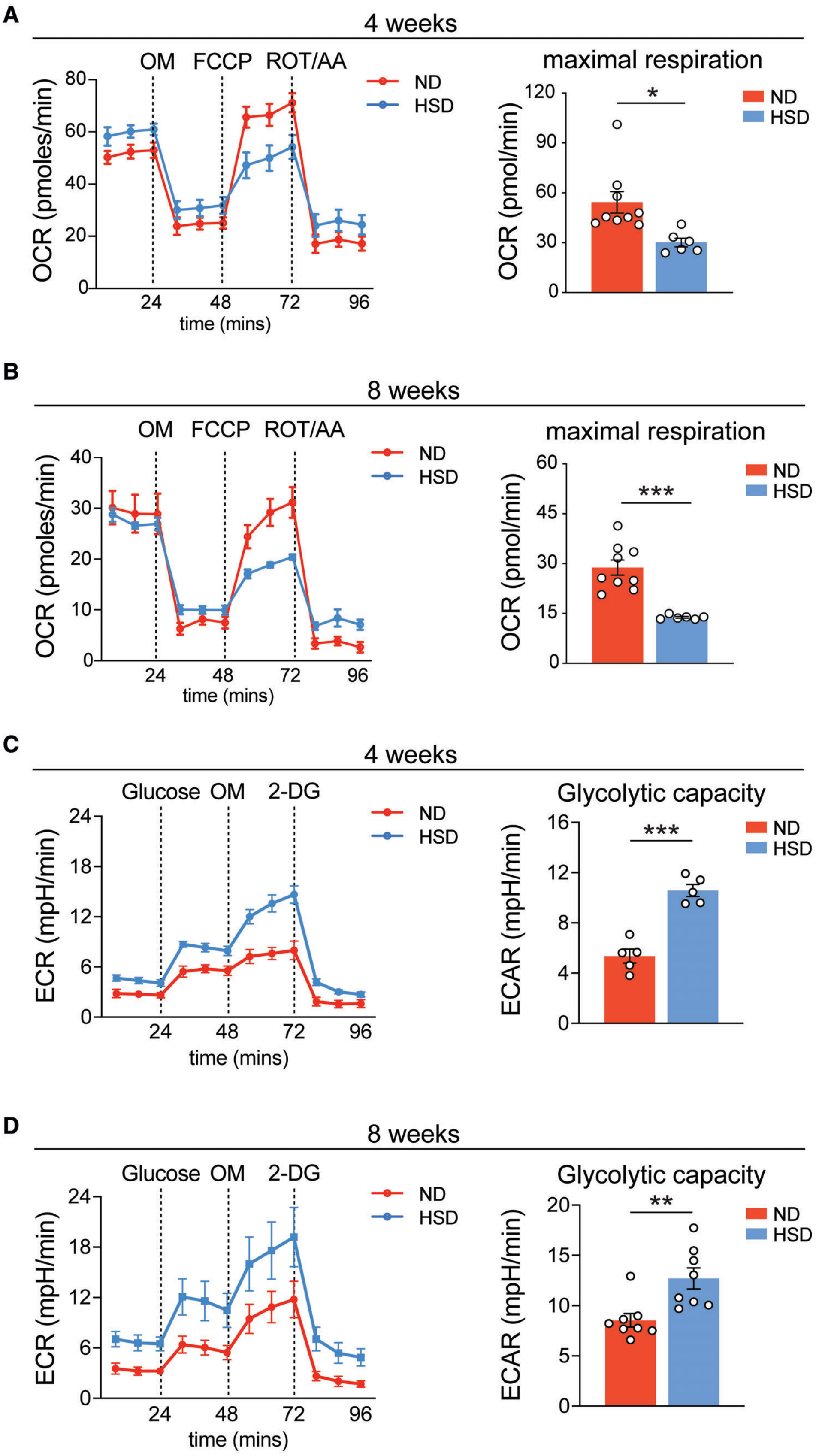

Figure EV3.

© 2023 The Authors

Figure EV4. Four-week HSD reduces ICH recovery, HSD➔ND chimeric mice have worse functional outcomes after collagenase ICH, and ND BM cell transplantation to HSD mice rescues HSD-aggravated ICH injuries.

A Schematic of the 4-week diet regimen. Six-week-old mice were fed with ND or HSD for 4 weeks before being subjected to ICH surgery. Behavioral tests and flow cytometry were performed 1 week after blood ICH induction.

B Cylinder test results of mice after 4-week ND and HSD on days 1, 3, and 7 after ICH. $n = 9$ ND, 11 HSD, two-way ANOVA and Bonferroni test. *n*: biological replicates.

C Percentages of $CD36^+$, $CD206^+$, and $CD36^+CD206^+$ MDMs in 4-week ND and HSD mice after ICH. $n = 5$/group, Student's *t*-test. *n*: biological replicates.

D Percentages of $CD36^+$ or $CD206^+$ microglia (MG) after 4-week ND and HSD. $n = 5$/group, Student's *t*-test. *n*: biological replicates.

E Survival rates of ND and HSD chimeras that were subjected to a collagenase ICH model.

F Cylinder test and forelimb placement test results of ND➔ND and HSD➔ND chimeras. $n = 6$ ND chimeras and 3 HSD chimeras on day 3, $n = 4$ ND chimeras and 2 HSD chimeras on day 10.

G Schematic of experimental design. ND and HSD BM were transplanted into HSD recipients.

H Cylinder test of ND➔HSD ($n = 7$) and HSD➔HSD ($n = 8$) chimeric mice on days 3, 7, and 10 post-ICH. Two-way ANOVA and Bonferroni test. *n*: biological replicates.

I Representative images and quantification of $Ly76^+$ hematomas co-labeled with DAPI in ND➔HSD ($n = 7$) and HSD➔HSD ($n = 8$) chimeras. Student's *t*-test. *n*: biological replicates. Scale bar: 500 μm.

J Representative images and quantifications of $NeuN^+$ neurons and $GFAP^+$ cells co-labeled with DAPI in ND➔HSD ($n = 7$) and HSD➔HSD ($n = 8$) chimeras. Student's *t*-test. *n*: biological replicates. Scale bar: 100 μm.

K Representative images and quantifications of $CD206^+IBA1^+$ and $MAC2^+IBA1^+$ cells in ND➔HSD ($n = 8$) and HSD➔HSD ($n = 8$) chimeras. Student's *t*-test. *n*: biological replicates. Scale bar: 100 μm.

Data information: All data are mean ± SEM, each symbol represents one mouse or one biological replicate, $^*P < 0.05$, $^{**}P < 0.01$, and $^{***}P < 0.001$, n.s., not significant. Source data are available online for this figure.

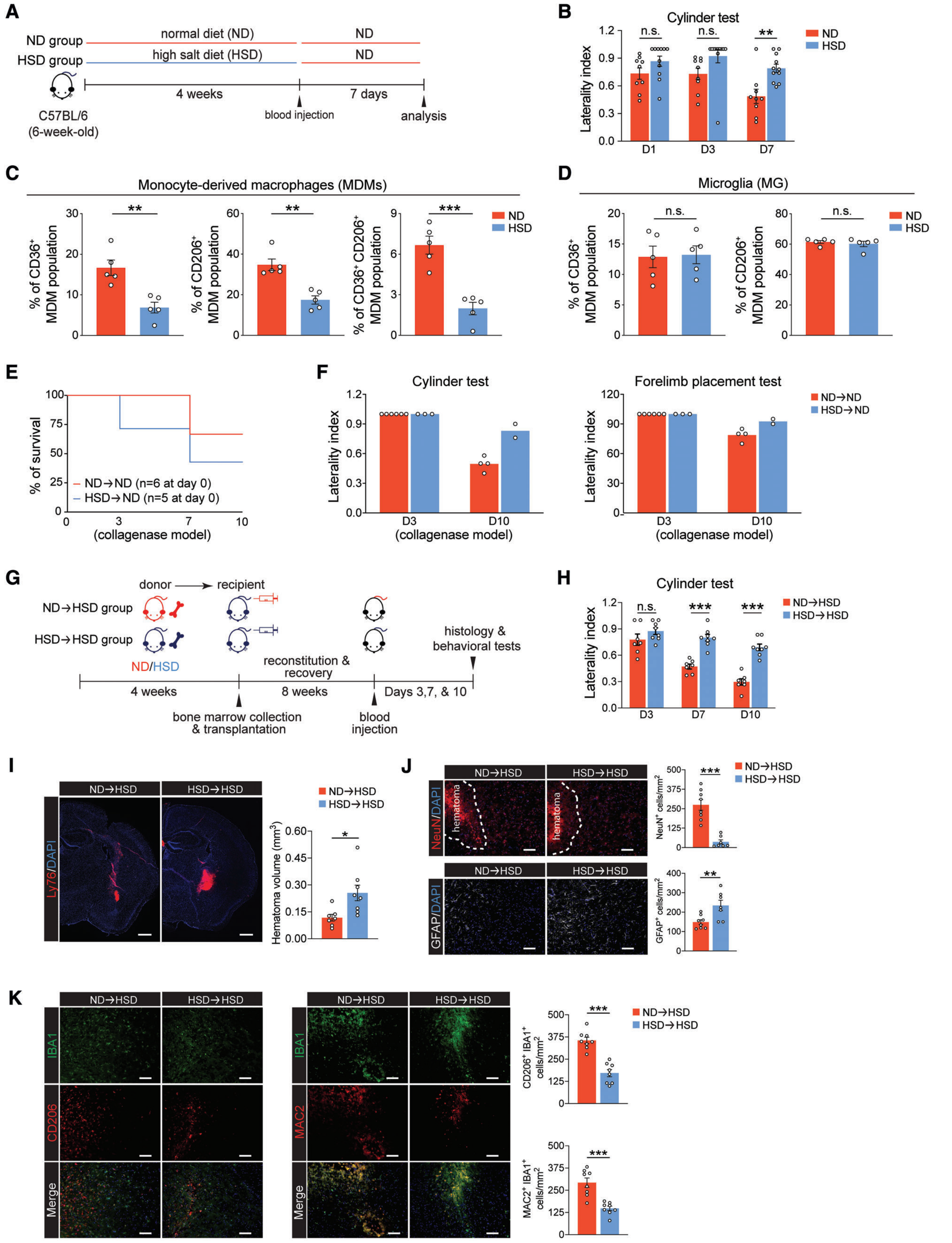

Figure EV4.

© 2023 The Authors

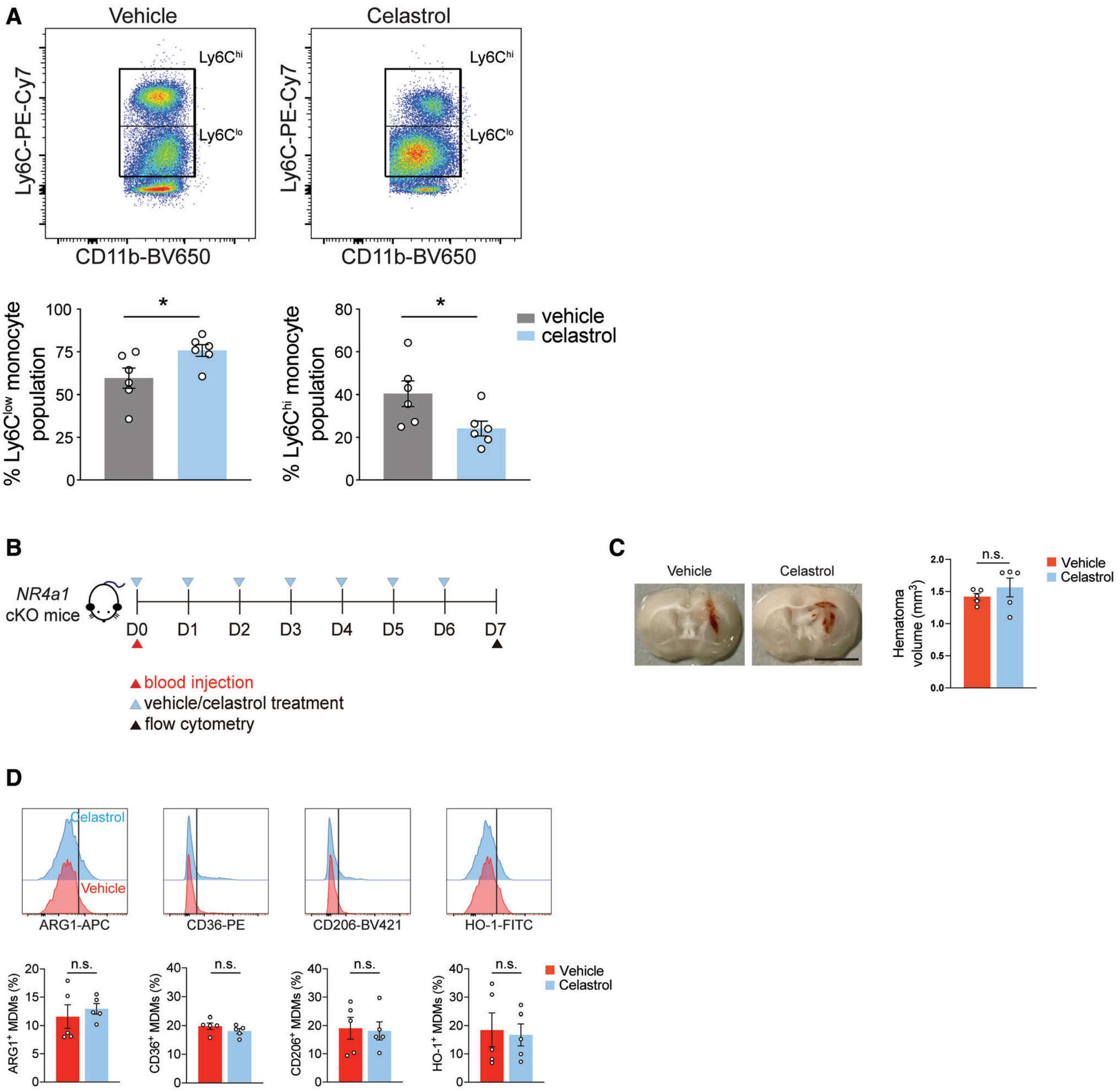

Figure EV5. Celastrol treatment increases Ly6C^{low} monocytes and decreases Ly6C^{hi} proinflammatory monocytes, and deletion of NR4a family in macrophages abolishes the protective effects of celastrol in the ICH brain.

A Top: representative pseudocolor plots showing Ly6C^{hi} and Ly6C^{low} monocytes in HSD mice that received vehicle or celastrol treatments. Bottom: quantifications showing percentages of Ly6C^{low} and Ly6C^{hi} monocytes in vehicle and celastrol groups. n = 6/group, Student's t-test. n: biological replicates.

B Schematic of celastrol treatment in *NR4a1* cKO mice and experimental design.

C Representative images and quantification of brain hematoma in vehicle- and celastrol-treated *NR4a1* cKO mice on day 7 post-ICH. n = 5/group, Student's t-test. n: biological replicates. Scale bar: 5 mm.

D Representative histograms and quantifications of ARG1$^+$, CD36$^+$, CD206$^+$, and HO-1$^+$ MDMs in vehicle- and celastrol-treated *NR4a1* cKO ICH mice. n = 5/group, Student's t-test. n: biological replicates.

Data information: All data are mean ± SEM, each symbol represents one mouse, *$P < 0.05$, n.s., not significant.
Source data are available online for this figure.
