# Supplementary material for: Trained immunity induced by high‐salt diet impedes stroke recovery
Source: EMBO Rep. 2023 Nov 15;24(12):e57164. doi: 10.15252/embr.202357164 (PMC10702837; doi:10.15252/embr.202357164)
Supplement: Supplementary file 5 — PDF+ [file EMBR-24-e57164-s008.pdf]

Article

SOURCE DATA TRANSPARENT PROCESS OPEN ACCESS

EMBO reports

Trained immunity induced by high-salt diet impedes stroke recovery

Tze-Yen Lin[1], Danye Jiang[2], Wan-Ru Chen[1,3], Jhih Syuan Lin[1], Xin-Yu Zhang[1], Chih-Hung Chen[1], Chia-Lang Hsu[4], Liang-Chuan Lai[1], Ping-Hung Chen[5], Kai-Chien Yang[6], Lauren H Sansing[7] & Che-Feng Chang[1,*]

Abstract

A high-salt diet (HSD) elicits sustained sterile inflammation and worsens tissue injury. However, how this occurs after stroke, a leading cause of morbidity and mortality, remains unknown. Here, we report that HSD impairs long-term brain recovery after intracerebral hemorrhage, a severe form of stroke, despite salt withdrawal prior to the injury. Mechanistically, HSD induces innate immune priming and training in hematopoietic stem and progenitor cells (HSPCs) by downregulation of *NR4a* family and mitochondrial oxidative phosphorylation. This training compromises alternative activation of monocyte-derived macrophages (MDMs) without altering the initial inflammatory responses of the stroke brain. Healthy mice transplanted with bone marrow from HSD-fed mice retain signatures of reduced MDM reparative functions, further confirming a persistent form of innate immune memory that originates in the bone marrow. Loss of NR4a1 in macrophages recapitulates HSD-induced negative impacts on stroke outcomes while gain of NR4a1 enables stroke recovery in HSD animals. Together, we provide the first evidence that links HSD-induced innate immune memory to the acquisition of persistent dysregulated inflammatory responses and unveils NR4a1 as a potential therapeutic target.

Keywords high-salt diet; intracerebral hemorrhage; macrophages; nr4a1; trained immunity

Subject Categories Immunology; Metabolism; Stem Cells & Regenerative Medicine

DOI 10.15252/embr.202357164 | Received 10 March 2023 | Revised 22 October 2023 | Accepted 26 October 2023

EMBO Reports (2023) e57164

Introduction

Salt (NaCl) has been an integral part of human history for thousands of years and is interwoven into our daily diets. While a small amount of salt is necessary for survival, excessive salt intake has spiraled into a public health crisis worldwide. A high-salt diet (HSD) is currently a leading dietary risk factor for non-communicable diseases and mortality (GBDD Collaborators, 2019) and has long been connected to development of cardiovascular disease through angiopathy and hypertension (Rucker *et al*, 2018). However, recent studies suggest HSD negatively affects cardiovascular function by impacting the immune system prior to the onset of hypertension (Faraco *et al*, 2018, 2019; Muller *et al*, 2019). Furthermore, HSD can inappropriately activate myeloid and lymphoid cells, which subsequently aggravate autoimmune responses, promote inflammatory tissue injury, and blunt host defense responses (Wu *et al*, 2013; Zhang *et al*, 2015; Jobin *et al*, 2020). Intriguingly, while salt reduction decreases cardiovascular events and death, reports indicate that salt-induced adverse immune responses cause persistent hepatic and renal injury and neuropathic pain—even after salt reduction/withdrawal (Oguchi *et al*, 2014; Fan *et al*, 2020; Gao *et al*, 2022). These findings suggest that the impact of salt on the immune system, especially in the context of prolonged inflammatory tissue injury, is not fully understood.

How could HSD alter immune cell functions and persistently influence pathological outcomes in diseases? Emerging evidence suggests that trained immunity, that is, *de facto* immunological memory in innate immune cells, could be involved. Through metabolic reprogramming and/or epigenetic modifications, the trained progeny of hematopoietic stem and progenitor cells (HSPCs) and tissue-resident macrophages can elicit either beneficial or detrimental responses, depending on the pathological condition (Netea *et al*, 2020). Recent studies indicate that HSPCs, monocytes, and macrophages establish innate immune memory after exposure to infectious stimuli, vaccination, and environmental challenges

1 Department and Graduate Institute of Physiology, National Taiwan University College of Medicine, Taipei, Taiwan
2 Department of Neurology, McGovern Medical School at the University of Texas Health Science Center in Houston, Houston, TX, USA
3 School of Medicine, National Taiwan University College of Medicine, Taipei, Taiwan
4 Department of Medical Research, National Taiwan University Hospital, Taipei, Taiwan
5 Department and Graduate Institute of Biochemistry and Molecular Biology, National Taiwan University College of Medicine, Taipei, Taiwan
6 Department and Graduate Institute of Pharmacology, National Taiwan University College of Medicine, Taipei, Taiwan
7 Department of Neurology, Yale University School of Medicine, New Haven, CT, USA
*Corresponding author. Tel: +886 2 23123456 ext. 288238; E-mail: chefengchang@ntu.edu.tw

© 2023 The Authors. Published under the terms of the CC BY 4.0 license.

(Bekkering *et al*, 2018; Christ *et al*, 2018; Wendeln *et al*, 2018; Yao *et al*, 2018; Zhang *et al*, 2021). In addition, unhealthy dietary patterns can trigger innate immune memory in bone marrow (BM) cells and aggravate disease pathogenesis (Christ *et al*, 2018; Edgar *et al*, 2021). However, whether dietary salt leads to dysfunctional myeloid cell responses through innate immune memory and the implications on cardiovascular event outcomes remain to be determined.

Stroke, a cardiovascular disease heavily influenced by dietary salt, is the second-leading cause of death and disability worldwide (GBDS Collaborators, 2021). Intracerebral hemorrhage (ICH), the most devastating subtype of stroke, has the highest mortality rate and is initiated by the rupture of a blood vessel within the brain that results in intraparenchymal hematoma. While the connection between salt intake and stroke risk is well established (GBDD Collaborators, 2019; GBDS Collaborators, 2021), far less is known about the effects of HSD on long-term stroke recovery, especially in ICH. Given that myeloid cells, predominantly macrophages, mediate long-term hematoma resolution, neuroinflammation, and neurobehavioral recovery after ICH (Zhao *et al*, 2017; Askenase *et al*, 2021; Chang *et al*, 2021), HSD might impact long-term stroke outcomes through regulating macrophage functions. Furthermore, it remains unknown whether (and how) HSD continuously affects myeloid cell functions after salt reduction during the chronic stage of stroke.

Here, we used murine stroke models to investigate how HSD alters stroke-induced innate immune responses and long-term disease outcomes. HSD modulates monocyte-derived macrophages (MDMs) toward a less reparative phenotype while sparing the parenchymal macrophages—microglia—in the ICH brain. This phenomenon occurred concomitantly with impeded hematoma clearance, reduced neurobehavioral recovery, and exacerbated neuron loss and astrogliosis. HSD also diminished oxidative phosphorylation (OXPHOS) in BM cells and this metabolic shift was retained by macrophages differentiated *in vitro*. We further confirmed a reduced macrophage reparative phenotype and impaired brain recovery in chimeric mice reconstituted with BM from mice fed with HSD. While HSD-induced myelopoiesis and systemic inflammation subsided after reverting to a normal diet, the negative impact of HSD on the macrophage phenotype and long-term stroke outcomes persisted. Transcriptomic analysis revealed that HSD induced metabolic and transcriptional reprogramming in HSPCs. Specifically, we identified the orphan nuclear receptor 4A1 (NR4a1/Nur77) as a potential therapeutic target for alleviation of HSD-induced adverse effects on stroke recovery. Overall, we uncovered a previously unknown role of HSD in modulating macrophage function and long-term brain recovery after stroke through innate immune training in the BM. These findings highlight the role of innate immune memory as a novel mechanism in acquisition of a disease-unfavorable immune response after HSD.

Results

HSD delays brain recovery and reduces the reparative macrophage phenotype after ICH

To investigate the effect of chronic high-sodium intake on brain recovery after stroke, we employed an HSD regimen based on previously established protocols (Faraco *et al*, 2019). Six-week-old mice received HSD (8% NaCl) or normal diet (ND; 0.5% NaCl) for 8 weeks before undergoing ICH surgery (Fig 1A). To better mimic the clinical scenario, all mice were returned to the ND after ICH. The HSD had no effect on daily food intake and body weight (Fig EV1A). Consistent with previous studies (Faraco *et al*, 2018), the HSD did not increase heart rate, mean arterial pressure, or systolic and diastolic pressures (Fig EV1B). To investigate the effect of the HSD on ICH outcomes during the recovery phase, we used two models of experimental ICH. Hematoma volume is the major determinant of patient outcomes (Broderick *et al*, 1993). Thus, we first induced hematoma using autologous blood injection and evaluated hematoma resolution and neurologic deficits on day 7 post-surgery, which marks the recovery phase in this ICH model (Fig 1A) (Chang *et al*, 2018; Tsai *et al*, 2022). HSD mice exhibited worse locomotor asymmetry in the cylinder test and exhibited larger hematoma volumes (Fig 1B and C). We also employed the collagenase ICH model, in which enzymatic digestion of the vessel walls produces severe brain injury and persistent functional deficits (Fig 1D) (Chang *et al*, 2018, 2020). A battery of behavioral experiments were performed to assess postural symmetry, gait, circling behavior, motor impairments, and sensorimotor integration from days 3 to 21 after ICH induction (Schaar *et al*, 2010). HSD mice exhibited reduced functional recovery compared to the ND group across these functional tests (Fig 1D and E). HSD mice also exhibited significantly increased red blood cell (RBC) deposition, exacerbated neuron loss, and higher numbers of glial fibrillary acidic protein (GFAP)-positive cells in the brain on day 21 post-ICH (Fig 1F).

Microglia, the brain parenchymal macrophages, and monocyte-derived macrophages (MDMs) are the main myeloid populations that accumulate in hemorrhagic regions (Chang *et al*, 2017, 2018). We and others have shown that both cell types polarize to a reparative phenotype and contribute to brain repair during the recovery phase (Zhao *et al*, 2007; Chang *et al*, 2017, 2021; Xu *et al*, 2020). Since a delayed brain repair was observed in the HSD animals, we assessed whether HSD affected alternative activation of these cell populations (Appendix Fig S1). On day 7 post-ICH, the HSD hemorrhagic brain displayed a lower percentage of reparative MDMs, as evidenced by reduced CD36 and CD206 expression (Fig 2A). The percentage of CD36 and CD206 double-positive MDMs was also significantly lower in HSD animals (Fig 2A). In confirmation with previous studies showing MDM alternative activation is essential for efficient erythrophagocytosis and hematoma clearance (Chang *et al*, 2018, 2020), we observed impaired MDM phagocytosis of erythrocytes in the HSD hemorrhagic brains (Fig 2B). Unexpectedly, the HSD-induced changes in alternative activation were unique to MDMs; no alterations in the percentages of $CD36^+$, $CD206^+$, $ARG1^+$, and $MerTK^+$ microglia were detected (Fig 2C). Furthermore, no significant difference in expressions of the proinflammatory genes *Tnf*, *Tspo*, and *Tmem119*, reparative gene *Apoe*, and epigenetic regulation genes *Hdac1* and *Hdac2* were observed in the ICH brain-sorted microglia during the recovery phase (day 7) between ND and HSD groups (Fig 2D). To exclude the possibility that the observed deficits in the HSD group were due to a more aggravated initial injury and a more proinflammatory MDM/microglial phenotype during the acute phase, we measured hematoma volumes at day 3 post-ICH and found that the two groups did not differ

 © 2023 The Authors

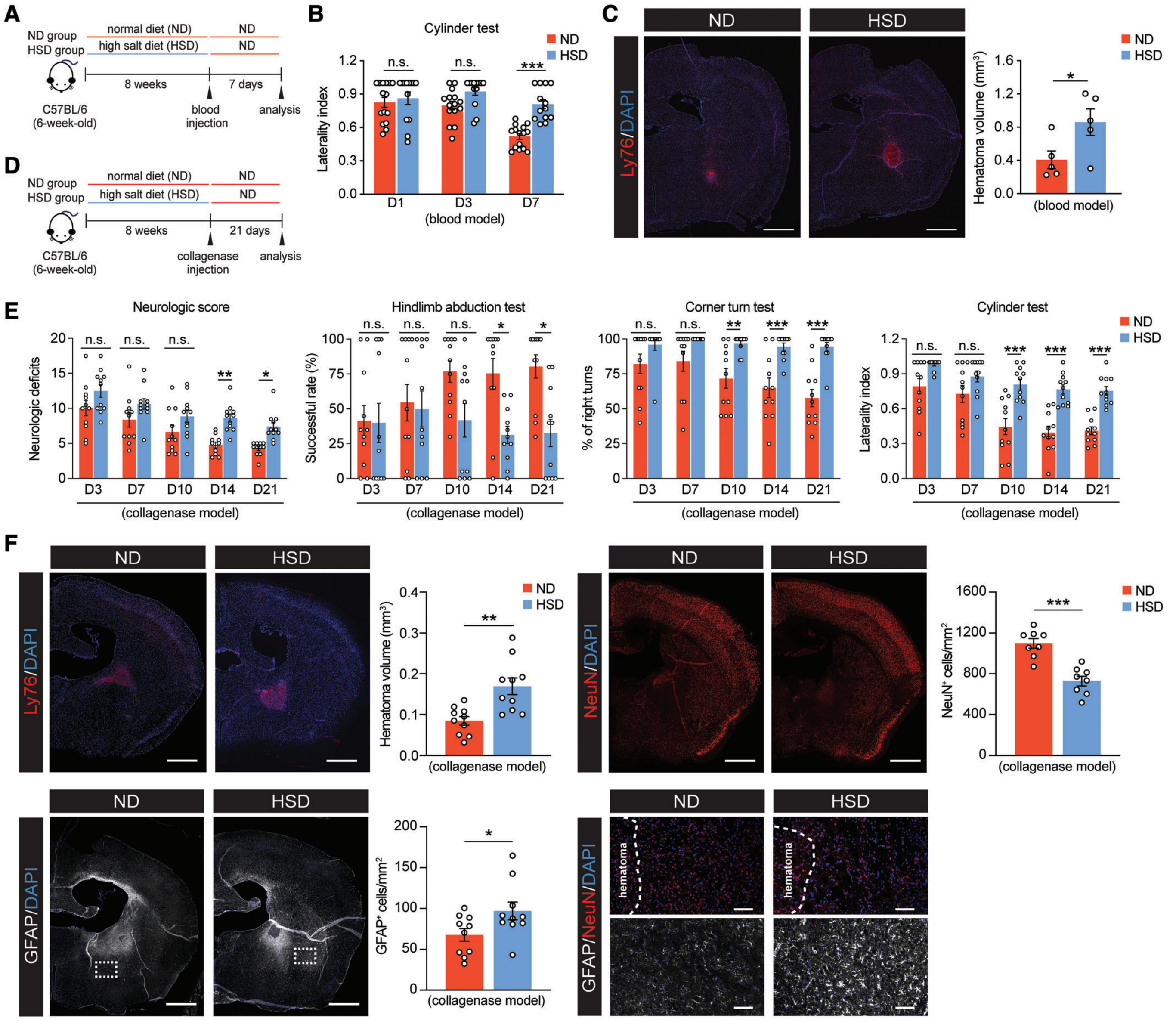

Figure 1. Effects of HSD on chronic recovery after ICH.

A Schematic of experimental design using a blood injection ICH model.

B Cylinder test of ND (n = 15) and HSD (n = 13) mice on days 1, 3, and 7 following blood injection, two-way ANOVA, and Bonferroni test. n: biological replicates.

C Representative images and quantification of Ly76$^+$ (red) hematoma co-labeled with DAPI in ND and HSD brains (n = 5/group) on day 7 post-ICH, Student's t-test. n: biological replicates. Scale bar: 1 mm.

D Schematic of experimental design using a collagenase injection ICH model.

E Neurologic score, hindlimb abduction test, corner turn test, and cylinder test for ND and HSD mice on days 3, 7, 10, 14, and 21 following ICH (n = 11/group), two-way ANOVA, and Bonferroni test. n: biological replicates.

F Representative images and quantification of Ly76$^+$ hematoma, NeuN$^+$ neurons, and GFAP$^+$ cells co-labeled with DAPI in ND and HSD mice on day 21 post-ICH. n = 10 for hematoma and GFAP quantification, n = 8 for NeuN quantification, Student's t-test. n: biological replicates. Scale bar: 1 mm, 100 µm for high-magnification insets.

Data information: All data are mean ± SEM, each symbol represents one mouse, *P < 0.05, **P < 0.01, and ***P < 0.001, n.s., not significant.
Source data are available online for this figure.

significantly (Fig 2E and F). Additionally, the frequencies of infiltrating MDMs and proinflammatory MDMs (MHCII$^+$, CD86$^+$, TNF$^+$, IL-1α$^+$, and IL-6$^+$) were also comparable between the two groups (Fig 2G and H). Moreover, similar to the recovery phase, no changes in the microglia population were detected during the acute phase (Fig 2I). Overall, these observations demonstrate that HSD delays brain recovery after ICH and specifically dampens the activation of reparative MDMs.

© 2023 The Authors

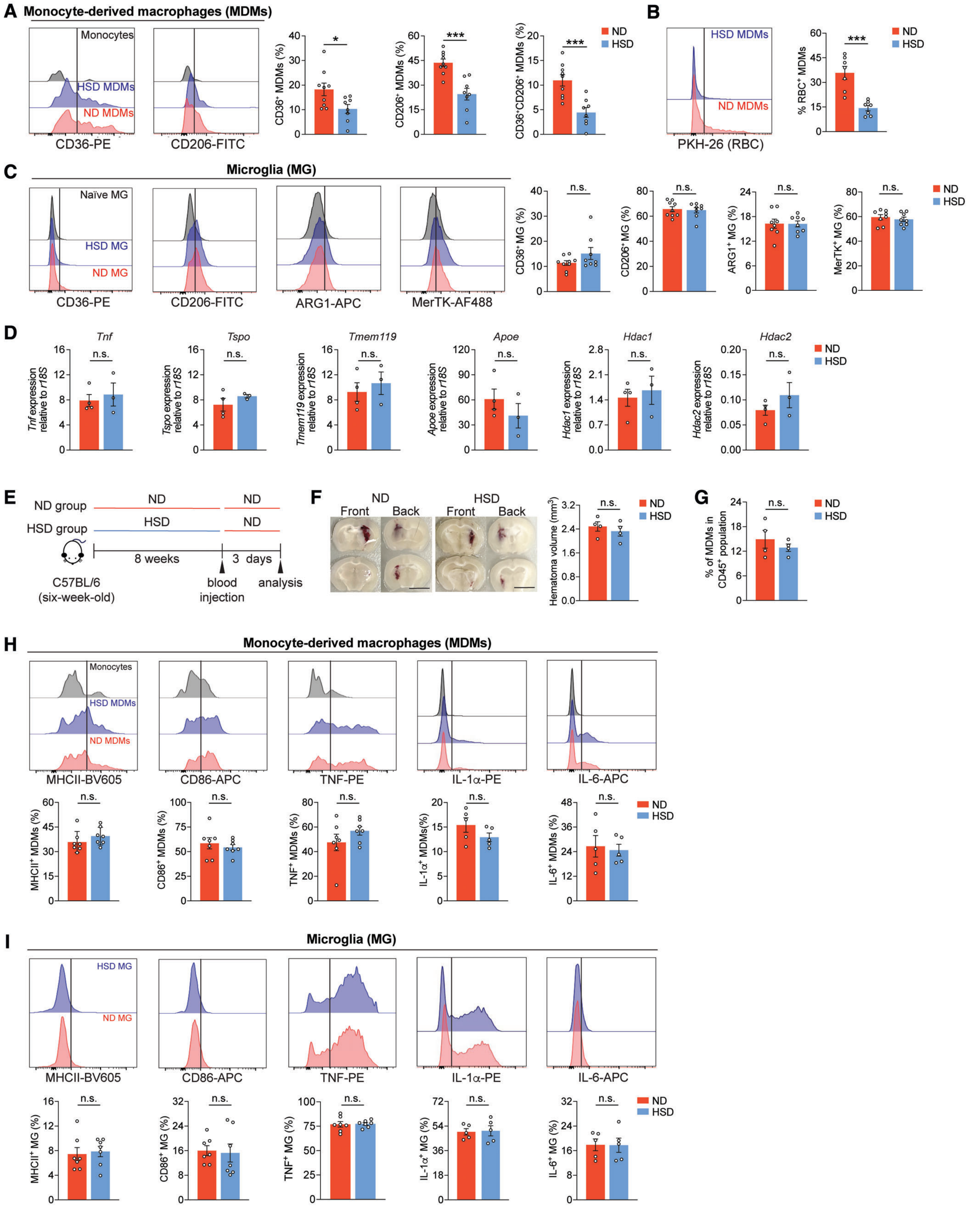

Figure 2.

© 2023 The Authors

Figure 2. HSD limits alternative activation of MDMs but not microglia.

A Left: representative histograms of CD36 and CD206 expression on monocytes (gray), HSD (blue), and ND (red) MDMs. Right: percentages of $CD36^+$, $CD206^+$, and $CD36^+CD206^+$ MDMs in ND (*n* = 9) and HSD (*n* = 8) mice, Student's *t*-test. *n*: biological replicates.

B Left: representative histogram shows intensity of fluorescently labeled erythrocytes in MDMs from ND and HSD mice at day 7 after ICH. Right: quantification of percentage of MDMs that contain labeled erythrocytes. n = 7/group, Student's *t*-test. *n*: biological replicates.

C Left: representative histograms of CD36, CD206, ARG1, and MerTK expression on naive (gray), HSD (blue), and ND (red) microglia. Right: percentages of $CD36^+$, $CD206^+$, $ARG1^+$, and $MerTK^+$ microglia in ND (*n* = 8–9) and HSD (*n* = 8) mice. Student's *t*-test. *n*: biological replicates.

D Gene expression of proinflammatory markers (*Tnf*, *Tspo*, and *Tmem119*), reparative phenotype (*Apoe*), and epigenetic regulation (*Hdac1* and *Hdac2*) in microglia from ND (*n* = 4) and HSD (*n* = 3) ICH brains. Student's *t*-test. *n*: biological replicates, each biological replicate pooled from three independent animals.

E Schematic of investigating initial ICH injury following 8-week ND or HSD. Histological assay and flow cytometry were performed 3 days after blood ICH induction.

F Left: representative coronal sections show visible hematoma in the ND and HSD mice on day 3 after blood injection. Right: quantification of hematoma volume. *n* = 4/group, Student's *t*-test. Scale bar: 5 mm. *n*: biological replicates.

G Percentages of brain-infiltrating MDMs from ND and HSD animals on day 3 post-ICH. *n* = 4/group, Student's *t*-test. *n*: biological replicates.

H Top: representative histograms showing MHCII, CD86, TNF, IL-1α, and IL-6 expressions on monocytes (gray), HSD MDMs (blue), and ND MDMs (red). Bottom: quantifications showing percentages of $MHCII^+$, $CD86^+$, TNF^+, IL-$1\alpha^+$, and IL-6^+ MDMs between ND and HSD groups. *n* = 5-7/group, Student's *t*-test. *n*: biological replicates.

I Top: representative histograms showing MHCII, CD86, TNF, IL-1α, and IL-6 expressions on HSD (blue) and ND microglia (red). Bottom: quantifications showing percentages of $MHCII^+$, $CD86^+$, TNF^+, IL-$1\alpha^+$, and IL-6^+ microglia (MG) between ND and HSD groups. *n* = 5-7/group, Student's *t*-test. *n*: biological replicates.

Data information: All data are mean ± SEM, each symbol represents one mouse unless stated otherwise, **P* < 0.05 and ****P* < 0.001; n.s., not significant. Source data are available online for this figure.

HSD reprograms bone marrow cells and their mature myeloid progeny

Both microglia and MDMs are alternatively activated during ICH recovery and exposed to a similar microenvironment surrounding the hematoma (Chang *et al*, 2018, 2021). Thus, the reduction in alternatively activated MDMs in HSD mice is unlikely to result entirely from "onsite education." To test whether macrophages in the peripheral organs also exhibit reduced alternative polarization during inflammatory tissue injury, we first employed a thioglycollate-elicited model of peritonitis and collected peritoneal macrophages (pMFs) at the proinflammatory and pro-resolving stages (Bosurgi *et al*, 2017) (Fig EV2A). The percentages of $CD36^+$ and $CD206^+$ pMFs increased from day 3 to 7 after thioglycollate injection, indicating transition of peritoneal macrophages into a reparative phenotype (Fig EV2B). As expected, thioglycollate-injected HSD mice exhibited a lower percentage of $CD36^+$ pMFs at day 7 than ND mice (Fig EV2B). We then performed an erythrophagocytosis assay by measuring the engulfment of fluorescently labeled heat-shocked erythrocytes by pMFs in the presence and absence of *in vitro* stimuli that replicate the complex ICH milieu *in vivo*. HSD pMFs exhibited reduced erythrocyte engulfment under both unstimulated control and thrombin-stimulated conditions, suggesting a general reduction in erythrophagocytosis in HSD pMFs (Fig EV2C). These results demonstrate a widespread reduction in reparative macrophages regardless of the type of inflamed tissue.

Diet-induced systemic immune activation can stimulate myeloid progenitors in the bone marrow (BM) through distinct metabolic mechanisms and give rise to maladaptive monocytes and macrophages in several diseases (Christ *et al*, 2018; Edgar *et al*, 2021). In addition, macrophages undergo mitochondrial oxidative phosphorylation (OXPHOS) to repolarize to and maintain a reparative phenotype (van den Bossche *et al*, 2016). Thus, we investigated whether HSD induces metabolic reprogramming in BM cells and their mature progeny. We first assessed mitochondrial oxidative activity and glycolysis by measuring the oxygen consumption rate (OCR) and extracellular acidification rate (ECAR), respectively. BM cells from both 4- and 8-week HSD mice displayed reduced OXPHOS and increased glycolytic capacity compared to those from ND mice, suggesting HSD induces a metabolic switch in BM cells (Fig EV3A–D). To investigate whether this HSD-induced metabolic switch is maintained in the progeny of BM cells, we differentiated BM cells into bone marrow-derived macrophages (BMDMs) *in vitro* (Fig 3A). BMDMs derived from 8-week HSD BM cells retained the same metabolic signatures, with significantly reduced basal respiration, ATP production, and maximal respiration compared to ND BMDMs (Fig 3B). After stimulation with the ICH mimic thrombin (Babu *et al*, 2012), HSD BMDMs exhibited lower expression of *Apoe*, *Arg1*, and *Trem2* (Fig 3C and D), suggesting the lower OCR of BMDMs differentiated from HSD BM cells correlated with a reduction in their reparative response to inflammatory milieu. Moreover, ND BMDMs differentiated in the presence of an additional 40 mM of NaCl (179 mM) exhibited lower expression of these same reparative genes after thrombin stimulation compared to BMDMs differentiated in normal media (139 mM NaCl) (Fig 3D).

To obtain further *in vivo* evidence that HSD alters the properties of BM cells, we created bone marrow chimeric mice using a 4-week dietary regimen. We first confirmed a similarly reduced neurobehavioral recovery and decreased MDM reparative marker expression after ICH induction in the 4-week HSD mice compared to ND mice (Fig EV4A–D). ND hosts were irradiated and reconstituted with BM from either ND donors (ND➔ND chimeras) or HSD donors (HSD➔ND chimeras) after 4-week dietary manipulation (Fig 3E). After an 8-week recovery following transplantation, we modeled ICH by blood injection. HSD➔ND chimeras exhibited worse locomotor asymmetry on the cylinder test than ND➔ND chimeras (Fig 3F). HSD➔ND chimeras also had larger hematoma volumes, exacerbated neuron loss, and more $GFAP^+$ cells on day 10 post-ICH (Fig 3G and H). A reduction in the numbers of $CD206^+IBA1^+$ and $MAC2^+IBA1^+$ cells was also detected in the HSD➔ND chimeras when compared to the ND➔ND group (Fig 3I), suggesting diminished reparative macrophages in the HSD➔ND chimeras. We also leveraged the more severe brain injury of the collagenase ICH model and observed a higher mortality rate and a trend toward poorer neurobehavioral outcomes in the HSD➔ND chimeras (Fig EV4E and F). To further confirm and extend our findings, we tested whether ND BM was

sufficient to rescue HSD-induced deficits. We transplanted either ND (ND➔HSD chimeras) or HSD (HSD➔HSD chimeras) BM cells into the irradiated HSD recipient mice (Fig EV4G). After ICH induction, HSD mice transplanted with ND BM cells exhibited better neurobehavioral recovery than those received HSD BM cell transplantation (Fig EV4H). In parallel, the ND➔HSD chimeras also had reduced hematoma volumes, lessened neuron loss and astrogliosis, and higher numbers of CD206$^+$IBA1$^+$ and MAC2$^+$IBA1$^+$ reparative macrophages on day 10 post-ICH (Fig EV4I–K). Taken together, these results suggest that HSD induces immunological memory in BM

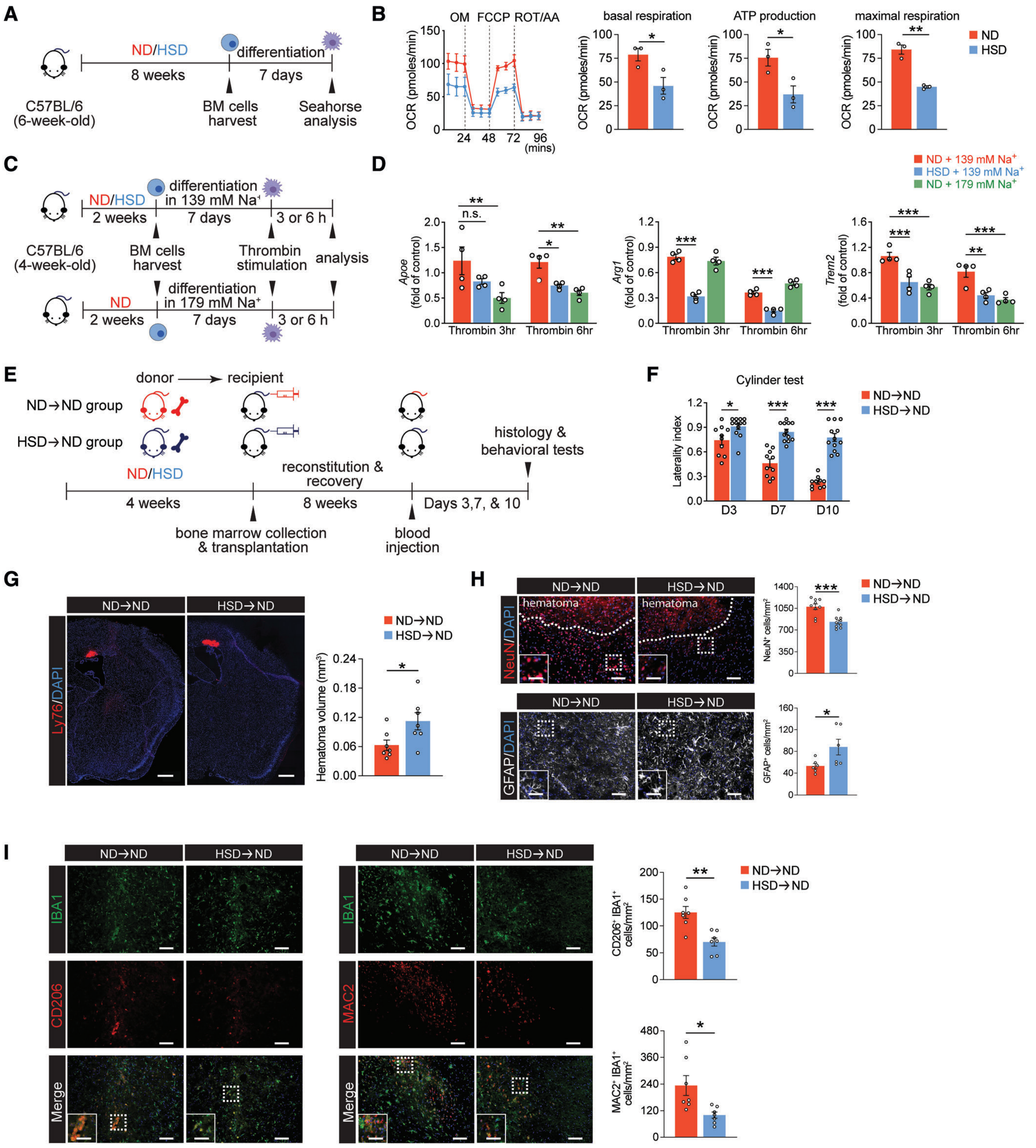

Figure 3.

 © 2023 The Authors

◀ **Figure 3. HSD-induced immunological memory in BM cells contributes to reduced macrophage reparative phenotype and can be transferred to ND mice.**

A Schematic of experimental design.
B OCR traces and quantifications of basal respiration, ATP production, and maximal respiration of BMDMs derived from ND (red) and HSD (blue) BM. $n = 3$/group, Student's *t*-test. *n*: biological replicates, each averaged from $n = 2$–3 technical replicates.
C Schematic of *in vitro* differentiation of ND and HSD BM cells into BMDMs under different sodium concentrations.
D Gene expression of reparative markers *Apoe*, *Arg1*, and *Trem2* in BMDMs derived from ND BM under normal sodium conditions (red), HSD BM under normal sodium conditions (blue), and ND BM under high-sodium conditions (green) upon thrombin stimulation. $n = 4$/group, two-way ANOVA and Bonferroni test. *n*: biological replicates.
E Schematic of experimental design for ND➔ND and HSD➔ND BM chimeras.
F Cylinder test of ND➔ND ($n = 10$) and HSD➔ND ($n = 12$) chimeras on days 3, 7, and 10 post-ICH. Two-way ANOVA and Bonferroni test. *n*: biological replicates.
G Representative images and quantification of Ly76$^+$ hematomas co-labeled with DAPI in ND➔ND and HSD➔ND chimeras. $n = 7$/group, Student's *t*-test. *n*: biological replicates. Scale bar: 500 μm.
H Representative images and quantifications of NeuN$^+$ neurons and GFAP$^+$ cells co-labeled with DAPI in ND➔ND and HSD➔ND chimeras. $n = 9$/group for NeuN, $n = 6$/group for GFAP, Student's *t*-test. *n*: biological replicates. Scale bar: 100 μm and 50 μm for high-magnification insets.
I Representative images and quantifications of CD206$^+$IBA1$^+$ and MAC2$^+$IBA1$^+$ cells in ND➔ND and HSD➔ND chimeras. $n = 7$/group, Student's *t*-test. *n*: biological replicates. Scale bar: 100 μm and 50 μm for high-magnification insets.

Data information: All data are mean ± SEM, each symbol represents one mouse or one biological replicate, $*P < 0.05$, $**P < 0.01$, and $***P < 0.001$, n.s., not significant. Source data are available online for this figure.

cells and that their myeloid progeny inherit similar cellular characteristics.

HSD-induced innate immune memory adversely impacts long-term recovery even after salt reduction prior to a stroke

High-sodium intake increases the number of circulating inflammatory monocytes and cytokine production (Wenstedt *et al*, 2019). Chronic systemic inflammation-elicited myelopoiesis and myeloid progenitor cell pool remodeling have been suggested to build immunological memory in BM progenitor cells (Mitroulis *et al*, 2018). Therefore, we ascertained to what extent the HSD-induced immunological memory persists in BM progenitor cells and how that impacts the response of MDMs in stroke recovery after salt reduction. Enumeration of BM progenitor cells revealed that 8-week HSD increased the percentage of granulocyte–monocyte progenitor (GMPs) (Fig 4A and B). HSD-induced GMP proliferation was concomitant with increased frequency of circulating CD11b$^+$Ly6C^{hi} proinflammatory monocytes (Fig 4C), suggesting a systemic inflammatory response. We then reverted the animals from HSD to ND for 1 week and evaluated inflammation in these animals (Fig 5A). No significant differences in the percentages of HSPCs, GMPs, or the CD11b$^+$Ly6C^{hi} population were observed between the ND-fed mice (ND-ND) and HSD-fed mice (HSD-ND) after returning to ND for 1 week (Fig 4D and E), suggesting the previously observed systemic inflammation subsided after salt reduction. Next, we modeled hemorrhagic stroke by autologous blood injection in these animals. On day 7 after ICH, we observed reduced functional recovery in HSD-ND mice compared to the ND-ND group (Fig 5B). The HSD-ND group also exhibited decreased hematoma clearance, higher neuron loss, and astrogliosis (Fig 5C), and reductions in the numbers of CD206$^+$IBA1$^+$ and MAC2$^+$IBA1$^+$ reparative MDMs in the ICH brains (Fig 5D). In parallel, we also observed reduced percentages of CD206$^+$ and CD36$^+$ reparative MDMs in HSD mice that rested on ND compared to the ND-ND group (Fig 5E). Notably, the frequencies of ICH brain-infiltrating MDMs and percentages of CD86$^+$ and MHCII$^+$ proinflammatory MDMs did not differ between ND-ND and HSD-ND mice on day 7 post-ICH (Fig 5F). These results suggest the reduction in reparative MDMs observed in HSD-ND mice was not due to a reduction in the total number of infiltrating MDMs or an increase in proinflammatory macrophages. Consistent with previous results (Figs 2 and EV4), expression of the proinflammatory and reparative markers in microglia did not differ significantly between ND-ND and HSD-ND mice after ICH induction (Fig 5G), suggesting the HSD-induced alterations were unique to MDMs.

To test whether HSD-induced innate immune memory also impedes brain recovery in other types of CNS injury, we validated our findings in ischemic stroke using the transient middle cerebral artery occlusion model (tMCAO). Five days after surgery, the ischemic brains of HSD-ND mice exhibited a larger infarct volume, as evidenced by reductions in both Nissl stained-positive and neuron-specific microtubule-associated protein 2 (MAP-2)-positive areas (Fig 5H–J). Hyperplastic astrocytes have been reported to be neurotoxic and can hamper brain recovery after cerebral ischemia (Ito *et al*, 2019). Similarly, we observed an increased number of GFAP$^+$ cells in the ischemic brains of HSD-ND mice (Fig 5K). Taken together, these data further confirm that HSD-induced innate immune memory negatively impacts brain recovery after stroke.

HSD evokes transcriptional reprogramming of HSPCs

To explore the molecular mechanisms through which HSD induces innate immune memory, we performed bulk RNA sequencing (RNA-seq) of HSPCs from 8-week ND and HSD mice with or without ICH induction. We identified 289 differentially expressed genes (DEGs) in HSD HSPCs (Fig 6A, and Dataset EV1). Principal component analysis (PCA) revealed the two groups were transcriptionally distinct from each other (Fig 6B). Ingenuity pathway analysis (IPA) revealed HSD mostly affected pathways involved in glycolysis, cholesterol biosynthesis, NFAT-mediated regulation of the immune response, and pathways relevant to inflammatory responses such as the inflammasome and toll-like receptor signaling (Fig 6C, and Appendix Fig S2). These pathways include genes implicated in myelopoiesis (*Sphk1*, *Atf3*, and *Mmp8*) (Massberg *et al*, 2007; Golan *et al*, 2012; Liu *et al*, 2020), monocyte/macrophage commitment (*Spp1*) (Ruberti *et al*, 2018), the macrophage Ly6C^{hi} to Ly6C^{low} transition (*NR4a1*, *Nr1h3*, *S100a9*, and *Dab2*) (Hanna *et al*, 2012; Marinkovic *et al*, 2020), and liver X receptor (LXR) expression (*Nr1h3*) (Fig 6D). Additionally, gene set enrichment analysis (GSEA) using the Molecular Signatures Database (MSigDB) hallmark gene set

© 2023 The Authors

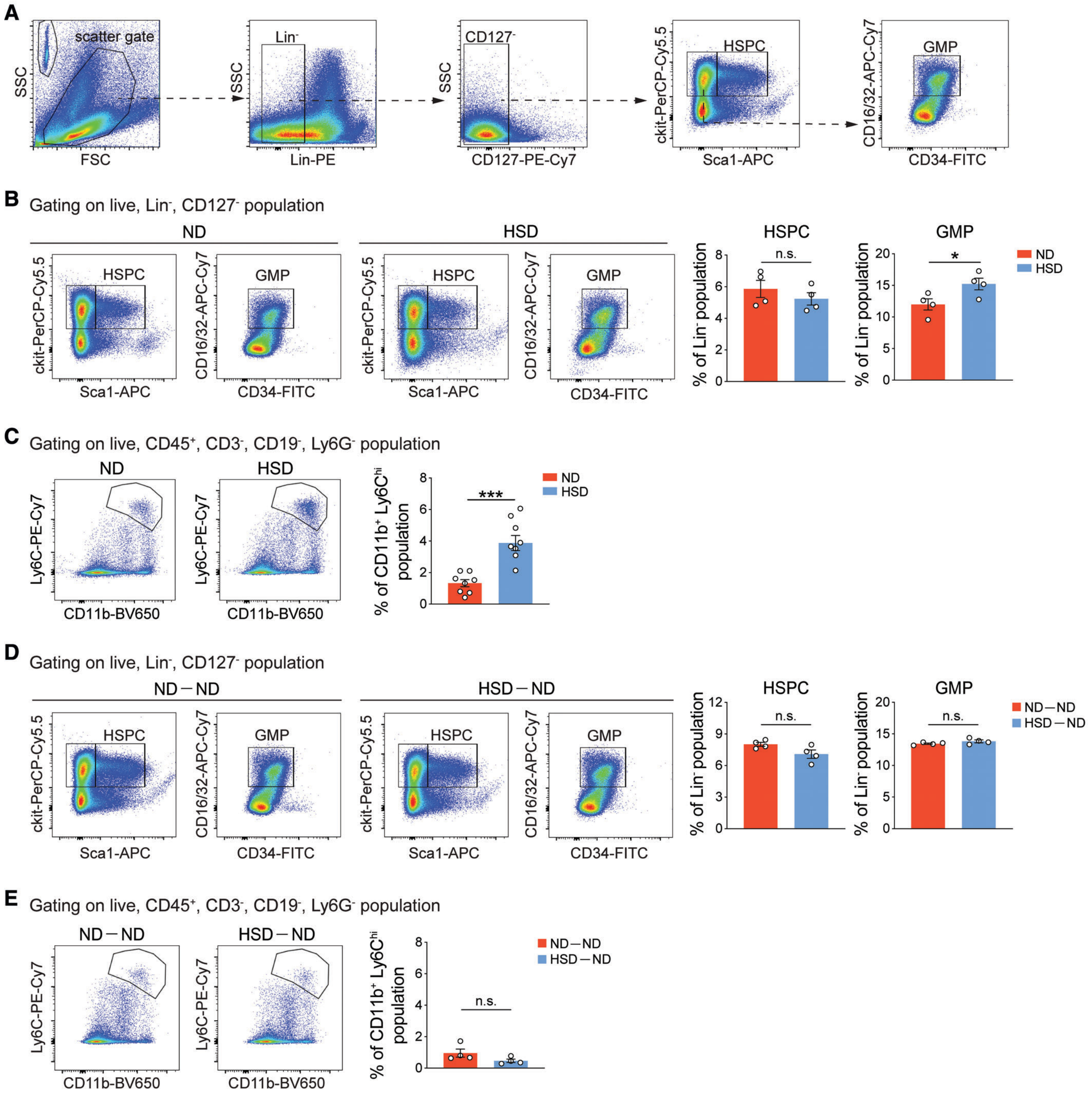

Figure 4. Gating strategies and quantifications of hematopoietic stem and progenitor cells (HSPCs) and granulocyte–monocyte progenitors (GMPs) after dietary manipulations.

- A Gating strategy of HSPCs and GMPs.
- B Left: representative pseudocolor plots of HSPCs and GMPs from ND and HSD mice. Right: quantifications showing percentages of GMPs and HSPCs in ND and HSD mice, respectively. $n = 4$/group, Student's *t*-test. *n*: biological replicates.
- C Left: representative pseudocolor plots of proinflammatory monocytes from blood samples of ND and HSD mice. Right: bar graph depicting percentages of proinflammatory monocytes in ND and HSD mice. $n = 8$/group, Student's *t*-test. *n*: biological replicates.
- D Left: representative pseudocolor plots of HSPCs and GMPs from ND-ND and HSD-ND mice. Right: quantifications depicting percentages of GMPs and HSPCs in these two groups. $n = 4$/group, Student's *t*-test. *n*: biological replicates.
- E Left: representative pseudocolor plots of proinflammatory monocytes from blood samples of ND-ND and HSD-ND mice. Right: bar graph depicting percentages of proinflammatory monocytes from these two groups. $n = 4$/group, Student's *t*-test. *n*: biological replicates.

Data information: All data are mean ± SEM, each symbol represents one mouse, *$P < 0.05$ and ***$P < 0.001$, n.s., not significant.
Source data are available online for this figure.

© 2023 The Authors

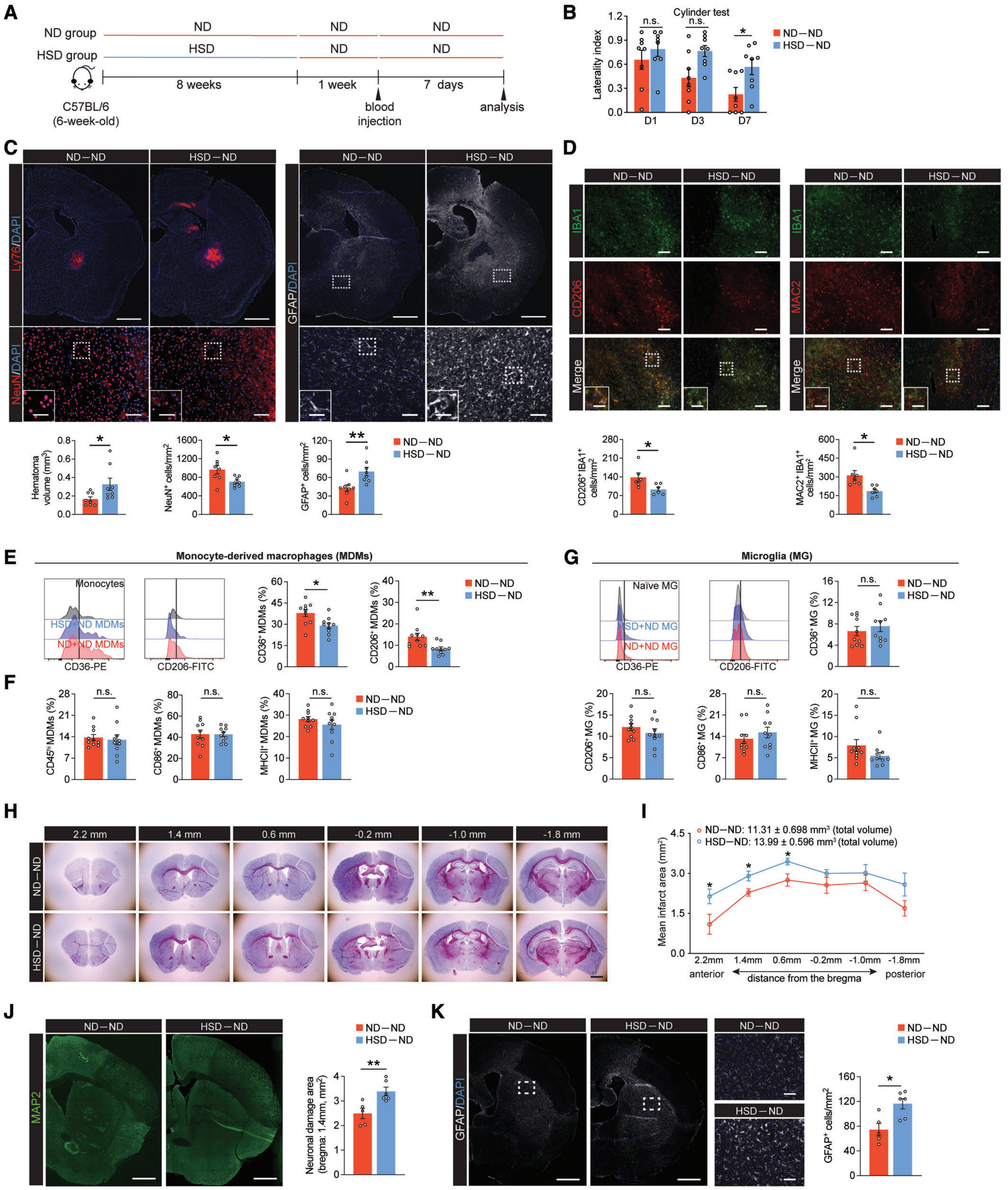
A
ND group
HSD group
ND
HSD
8 weeks
1 week
7 days
C57BL/6
(6-week-old)
blood
injection
analysis
B
Cylinder test
Laterality index
ND−ND
HSD−ND
D1
D3
D7
C
Ly76/DAPI
NeuN/DAPI
GFAP/DAPI
Hematoma volume (mm3)
NeuN+ cells/mm2
GFAP+ cells/mm2
D
IBA1
CD206
MAC2
Merge
CD206+ IBA1+ cells/mm2
MAC2+ IBA1+ cells/mm2
E
Monocyte-derived macrophages (MDMs)
Monocytes
HSD+ND MDMs
ND+ND MDMs
CD36-PE
CD206-FITC
CD36+ MDMs (%)
CD206+ MDMs (%)
F
CD45hi MDMs (%)
CD86+ MDMs (%)
MHCII+ MDMs (%)
G
Microglia (MG)
Naïve MG
HSD+ND MG
ND+ND MG
CD36+ MG (%)
CD206+ MG (%)
CD86+ MG (%)
MHCII+ MG (%)
H
2.2 mm
1.4 mm
0.6 mm
-0.2 mm
-1.0 mm
-1.8 mm
I
ND−ND: 11.31 ± 0.698 mm3 (total volume)
HSD−ND: 13.99 ± 0.596 mm3 (total volume)
Mean infarct area (mm2)
anterior
distance from the bregma
posterior
J
MAP2
Neuronal damage area (bregma: 1.4mm, mm2)
K
GFAP/DAPI
GFAP+ cells/mm2

Figure 5.

Figure 5. HSD-induced trained immunity adversely impacts hemorrhagic stroke and ischemic stroke recovery even if diet is normalized prior to stroke.

A Schematic of diet reversal from HSD to ND.
B Cylinder test of ND-ND and HSD-ND mice on days 1, 3, and 7 post-ICH. $n = 8$/group, two-way ANOVA and Bonferroni test. *n*: biological replicates.
C Representative images and quantification of Ly76$^+$ hematoma, NeuN$^+$ neurons, and GFAP$^+$ astrocytes co-labeled with DAPI in ND-ND and HSD-ND mice. $n = 8$/group, Student's *t*-test. *n*: biological replicates. Scale bar: 1 mm, 100 μm for high magnification, and 50 μm for high-magnification insets.
D Representative images and quantifications of CD206$^+$IBA1$^+$ ($n = 6$/group) and MAC2$^+$ IBA1$^+$cells ($n = 7$/group) in ND-ND and HSD-ND mice 7 days post-ICH. Student's *t*-test. *n*: biological replicates. Scale bar: 100 μm and 50 μm for high-magnification insets.
E Representative histograms of CD36 and CD206 expression on monocytes (gray), HSD-ND (blue), and ND-ND (red) MDMs at day 7 after ICH. Percentages of CD36$^+$ and CD206$^+$ MDMs in ND-ND and HSD-ND mice. $n = 10$/group, Student's *t*-test. *n*: biological replicates.
F Percentages of total infiltrating MDMs, CD86$^+$ MDMs, and MHCII$^+$ MDMs in ND-ND and HSD-ND mice. $n = 10$/group, Student's *t*-test. *n*: biological replicates.
G Representative histograms showing CD36 and CD206 expression on naive (gray), HSD-ND (blue), and ND-ND (red) microglia (MG). Percentages of CD36$^+$, CD206$^+$, CD86$^+$, and MHCII$^+$ MG in ND-ND and HSD-ND mice. $n = 10$/group, Student's *t*-test. *n*: biological replicates.
H Representative serial Nissl-stained coronal sections of ND-ND and HSD-ND brains using the transient middle cerebral artery occlusion (tMCAO) ischemic stroke model. Scale bar: 1 mm.
I Quantification of mean and total infarct volumes in ND-ND ($n = 5$) and HSD-ND ($n = 6$) mice. Student's *t*-test. *n*: biological replicates.
J Representative images of MAP2$^+$ staining and quantification of the neuronal damage area in ND-ND ($n = 5$) and HSD-ND ($n = 6$) brains. Student's *t*-test. *n*: biological replicates. Scale bar: 1 mm.
K Representative images and quantification of GFAP$^+$ cells in ND-ND ($n = 5$) and HSD-ND ($n = 6$) brains. Student's *t*-test. *n*: biological replicates. Scale bar: 1 mm and 100 μm for high-magnification insets.

Data information: All data are mean ± SEM, each symbol represents one mouse, $^*P < 0.05$ and $^{**}P < 0.01$, n.s., not significant.
Source data are available online for this figure.

collection revealed a correlation among HSD-induced HSPC transcriptional reprogramming, glycolysis, and fatty acid metabolism (Fig 6E). Transcription factors (TFs) responsible for epigenetic, metabolic, and inflammatory signaling integration (Zelcer & Tontonoz, 2006; Koenis *et al*, 2018; Liu *et al*, 2020; Zhang *et al*, 2022) (*Cecr2*, *NR4a3*, *Nr1h3*, *Atf3*, and *NR4a1; P*-values 5×10^{-2} to 1.5×10^{-6}) were also significantly differentially expressed in HSD HSPCs (Fig 6F). *NR4a1* and *NR4a3* were both downregulated in HSPCs from HSD-fed mice with or without ICH induction, suggesting these TFs consistently respond to HSD (Fig 6G, and Datasets EV1 and EV2). Collectively, these data corroborated our previous findings and provide evidence that HSD induces immunological training and metabolic alterations in HSPCs.

NR4a1 is a potential target to counter HSD-abrogated brain recovery after ICH

The transcriptomic analysis identified HSD downregulated *NR4a1* and *NR4a3* with or without ICH. These transcription factors belong to the NR4a family, which regulate the expansion of hematopoietic progenitors (Land *et al*, 2015; Freire & Conneely, 2018) and glucose metabolism (Corrocher *et al*, 2018; Deng *et al*, 2022) as well as inflammatory responses and mitochondrial metabolism in macrophages (Koenis *et al*, 2018). To assess whether the effects of HSD are mediated by the NR4a family, we generated an NR4a triple-knockout line (termed *NR4a* TKO) by crossing *NR4a3*$^{-/-}$ mice with mice expressing Cre recombinase under the myeloid-specific *LysM* promoter and carrying loxP-flanked alleles encoding NR4a1 and NR4a2 (Sekiya *et al*, 2013). At day 7 post-ICH, *NR4a* TKO mice exhibited more severe brain injury as evidenced by greater hematoma volumes and increased loss of neurons in the perihematomal region compared to littermate controls (*NR4a1*$^{fl/fl}$*NR4a2*$^{fl/fl}$*NR4a3*$^{+/+}$; Fig 7A). *NR4a* TKO mice also displayed lower numbers of MAC2$^+$IBA1$^+$ and ARG1$^+$IBA1$^+$ cells in the perihematomal region, suggesting diminished activation of reparative macrophages (Fig 7B). These histological deficits manifested in impaired neurobehavioral outcomes as *NR4a* TKO mice exhibited poorer performance in the cylinder test on days 3 and 7 post-ICH (Fig 7C). Together, deletion of the NR4a family receptors replicated the HSD-induced delay in brain recovery and reduced activation of reparative macrophages.

In particular, NR4a1 is involved in macrophage activation and differentiation. Mice deficient in NR4a1 lack Ly6C^{low} monocytes and the Ly6C^{hi}-to-Ly6C^{low} macrophage transition is impaired (Hanna *et al*, 2011, 2012; Carlin *et al*, 2013). To test whether NR4a1 alone can explain the phenomena observed after ICH, we selectively eliminated NR4a1 from myeloid cells by crossing conditional *NR4a1*$^{fl/fl}$ mice with a myeloid cell-specific Cre line *LysM*^{Cre}. We differentiated BM cells from these *NR4a1* cKO mice into BMDMs and assessed macrophage reparative phenotype upon stimulation with

Figure 6. HSD induces transcriptional reprogramming of HSPCs.

A Heatmap of differentially expressed genes (DEGs) determined by bulk sequencing of ND and HSD HSPCs ($n = 3$/group). Normalized *Z*-scores (high, red; low, blue) were calculated for each DEG (row). *n*: biological replicates, each biological replicate pooled from three independent animals.
B Principal component analysis (PCA) shows clear separation of the transcriptomes of HSD versus ND HSPCs.
C Ingenuity pathway analysis (IPA) revealed enrichment of several pathways in HSD HSPCs, including glycolysis, cholesterol biosynthesis, NFAT-mediated regulation of immune responses, inflammasome, and toll-like receptor signaling.
D Heatmap of DEGs involved in pathways related to inflammatory responses and cellular movement between ND HSPCs and HSD HSPCs.
E Gene set enrichment analysis (GSEA) of genes related to glycolysis and fatty acid metabolism. NES, normalized enrichment score; FDR, false discovery rate.
F Volcano plot showing the distribution of *P*-values ($-\log_{10}$ *P*-value) and their fold changes ($\log_2$ fold change). Significantly upregulated genes in HSD HSPCs are indicated in red and downregulated genes, in blue.
G Venn diagram showing overlap of DEGs between HSD HSPCs and ND HSPCs with or without ICH. *NR4a1* and *NR4a3* were both downregulated in HSPCs from HSD mice with or without ICH induction.

 © 2023 The Authors

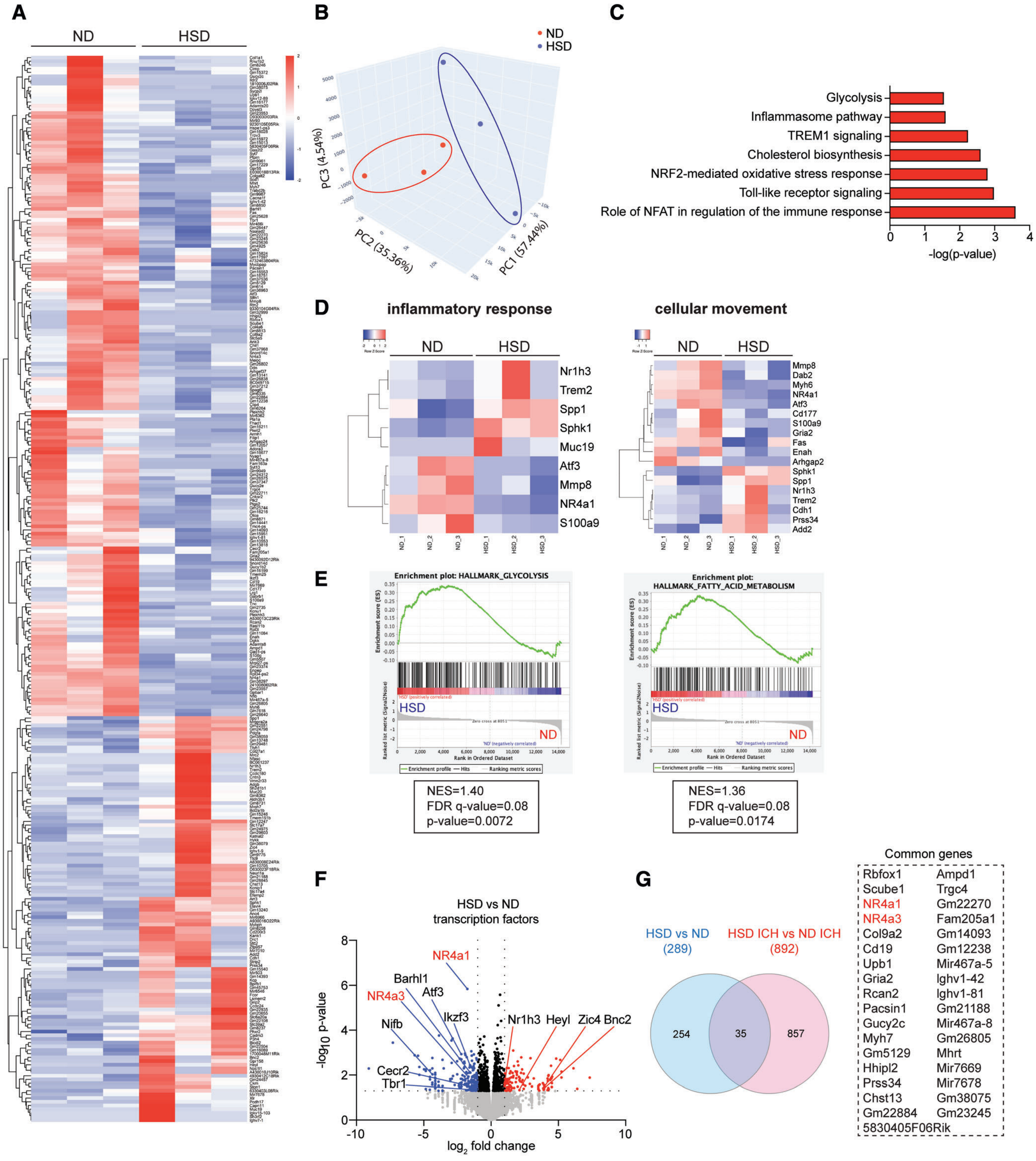

Figure 6.

interleukin-4 (IL-4), which induces alternative activation in macrophages (Bosurgi *et al*, 2017) and ICH tissue repair (Xu *et al*, 2020). *NR4a1* cKO BMDMs displayed decreased expression of reparative genes including *Trem2*, *Arg1*, and *Retnla* compared to BMDMs from control littermates (*NR4a1*$^{fl/fl}$), suggesting the reparative responses of BMDMs were impaired in the absence of NR4a1 (Fig 7D). To further assess the functional significance of NR4a1 *in vivo*, we analyzed percentages of reparative MDMs (CD36^{+}, ARG1^{+}, CD206^{+}, and HO-1^{+}) in the control and *NR4a1* cKO ICH brains. Loss of NR4a1 significantly reduced frequencies of CD36^{+}, ARG1^{+}, and CD206^{+} MDMs, while HO-1^{+} MDMs remained comparable between the two groups (Fig 7E). In line with this observation, *NR4a1* cKO mice showed

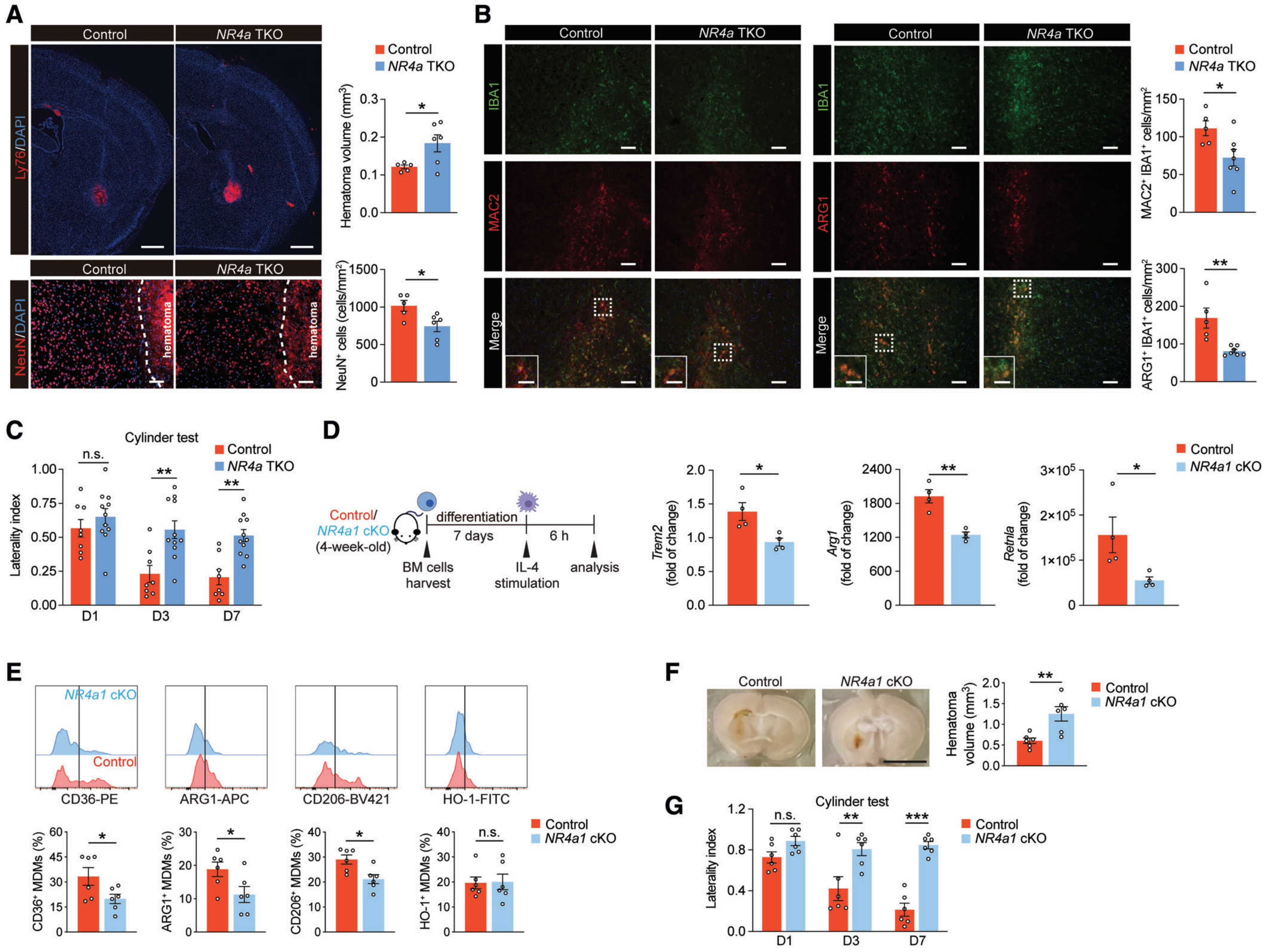

Figure 7. NR4a deletion in macrophages recapitulates the HSD-induced delay in ICH brain recovery and reduced alternative activation.

- A Representative images and quantification of Ly76$^+$ hematoma and NeuN$^+$ cells in control (n = 5) and NR4a triple conditional knockout (*NR4a* TKO, n = 6) brains on day 7 post-blood injection. Student's t-test. n: biological replicates. Scale bar: 1 mm for Ly76$^+$ hematoma and 100 μm for NeuN$^+$ cells.
- B Representative images and quantification of MAC2 and ARG1 expression in IBA1$^+$ cells in control (n = 5) and *NR4a* TKO (n = 7) mice. Student's t-test. n: biological replicates. Scale bar: 100 μm and 50 μm for high-magnification insets.
- C Cylinder test result of control (n = 8) and *NR4a* TKO mice (n = 11) on days 1, 3, and 7 post-ICH. Two-way ANOVA and Bonferroni test. n: biological replicates.
- D Left: schematic of *in vitro* differentiation of control and *NR4a1* conditional knockout (*NR4a1* cKO) BM cells into BMDMs. Right: quantification of gene expression of reparative markers *Trem2*, *Arg1*, and *Retnla* in BMDMs derived from control and *NR4a1* cKO BMs upon IL-4 stimulation. n = 4/group, Student's t-test. n: biological replicates.
- E Representative histograms and quantifications of CD36$^+$, ARG1$^+$, CD206$^+$, and HO-1$^+$ MDMs in control and *NR4a1* cKO ICH mice. n = 6/group, Student's t-test. n: biological replicates.
- F Representative images and quantification of hematoma in control and *NR4a1* cKO ICH mice on day 7 post-ICH. n = 6/group, Student's t-test. n: biological replicates. Scale bar: 5 mm.
- G Cylinder test of control and *NR4a1* cKO mice on days 1, 3, and 7 post-ICH. n = 6/group, two-way ANOVA and Bonferroni test. n: biological replicates.

Data information: All data are mean ± SEM, each symbol represents one mouse, *$P < 0.05$, **$P < 0.01$, and ***$P < 0.001$, n.s., not significant.
Source data are available online for this figure.

larger hematoma and delayed neurobehavioral recovery after ICH (Fig 7F and G). We next investigated whether overexpression of NR4a1 can rescue the reduced reparative phenotype in BMDMs differentiated under high-sodium conditions. We infected linage-negative BM cells from wild-type mice with lentiviruses encoding EGFP-NR4a1 or EGFP only (control) for 24 h before differentiating these cells into BMDMs under normal or high-sodium conditions (Fig 8A). Lentiviral overexpression of EGFP-NR4a1 and EGFP resulted in ~62% and ~74% transduction efficiency, respectively (Appendix Fig S3A). *NR4a1* was also significantly upregulated in NR4a1-overexpressing BMDMs (Appendix Fig S3B). BMDMs differentiated under a high-sodium condition from BM cells transduced with control virus showed a dampened reparative phenotype compared to cells differentiated under normal sodium conditions, as evidenced by reduced expression of *Trem2* and *Apoe*. Overexpression of NR4a1 in BMDMs differentiated under high-sodium

 © 2023 The Authors

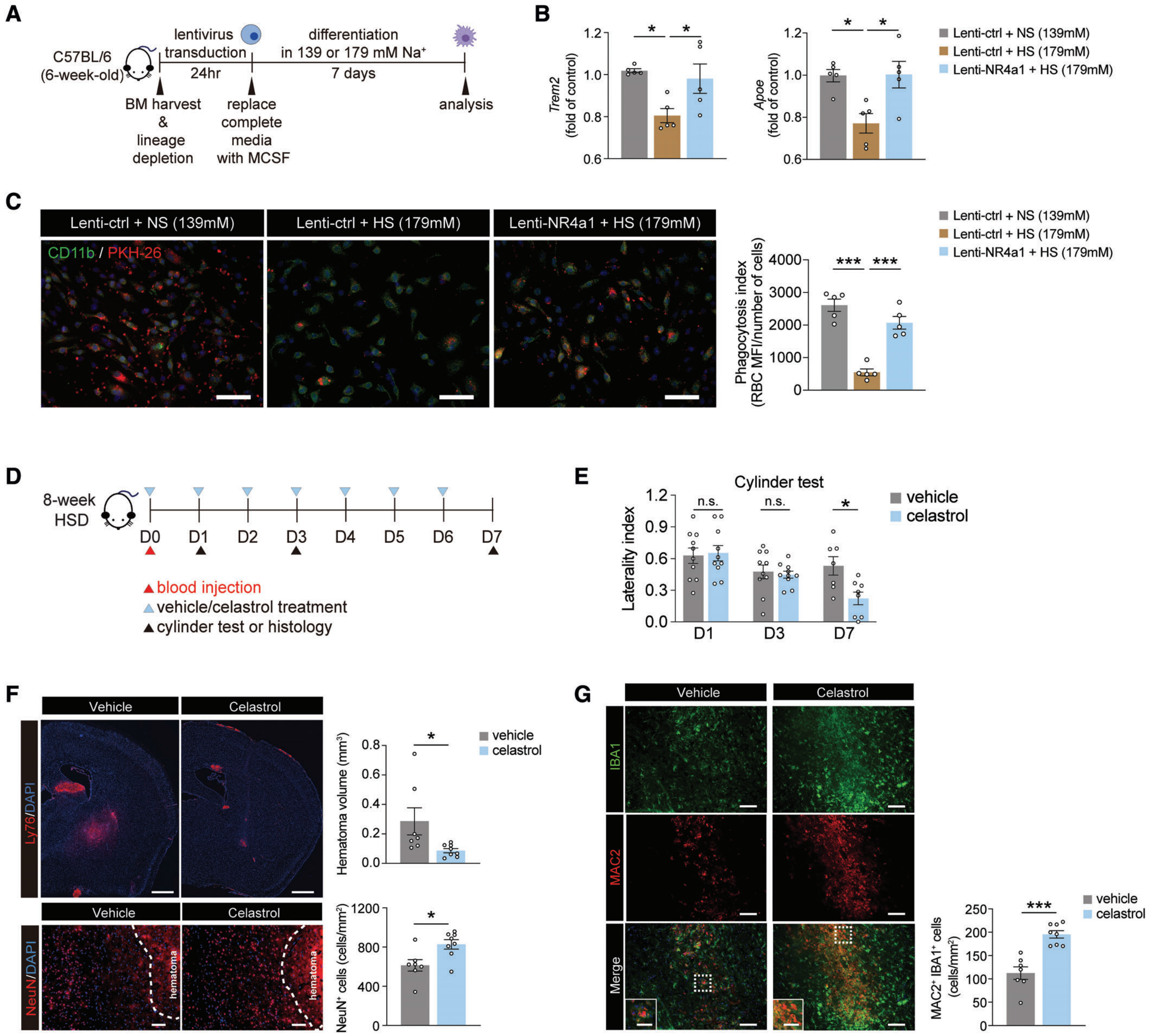

Figure 8. Manipulating NR4a1 modulates macrophage polarization and enables ICH recovery.

A Schematic of viral overexpression of NR4a1 in wild-type BM cells and subsequent *in vitro* differentiation into BMDMs under different sodium conditions.

B Gene expression of reparative markers *Trem2* and *Apoe* in BMDMs transduced with control lentivirus and differentiated under normal sodium (lenti-ctrl + NS), control lentivirus under high sodium (lenti-ctrl + HS), and NR4a1-overexpressing lentivirus under high sodium (lenti-NR4a1 + HS). $n = 5$/group, one-way ANOVA and Bonferroni test. *n*: biological replicates, each averaged from $n = 2$–3 technical replicates.

C Left: representative images showing engulfment of heat-shocked PKH26-labeled erythrocytes (red) by $CD11b^+$ (green) BMDMs transduced with control lentivirus and differentiated under normal sodium (lenti-ctrl + NS), control lentivirus under high sodium (lenti-ctrl + HS), and NR4a1-overexpressing lentivirus under high sodium (lenti-NR4a1 + HS). Scale bar: 50 μm. Right: quantifications of BMDM erythrophagocytosis. $n = 5$/group, one-way ANOVA and Bonferroni test. *n*: biological replicates.

D Schematic of celastrol treatment in HSD mice after a blood injection ICH.

E Cylinder test of HSD mice that received either vehicle or celastrol treatment $n = 10$/group, two-way ANOVA and Bonferroni. *n*: biological replicates.

F Representative images and quantification of $Ly76^+$ hematoma and $NeuN^+$ cells co-labeled with DAPI in mice receiving vehicle ($n = 7$) or celastrol ($n = 8$) treatment on day 7 post-ICH. Student's *t*-test. *n*: biological replicates. Scale bar: 1 mm for $Ly76^+$ hematoma and 100 μm for $NeuN^+$ cells.

G Representative images and quantification of MAC2 expression in $IBA1^+$ cells on day 7 post-ICH in the perihematomal brain regions of mice receiving vehicle ($n = 7$) or celastrol ($n = 8$). Student's *t*-test. *n*: biological replicates. Scale bar: 100 μm and 50 μm for high-magnification insets.

Data information: All data are mean ± SEM, each symbol represents one mouse or one biological replicate, $^*P < 0.05$ and $^{***}P < 0.001$, n.s., not significant.
Source data are available online for this figure.

conditions successfully restored *Trem2* and *Apoe* expression (Fig 8B). In line with this observation, BMDMs differentiated under high-sodium conditions exhibited decreased erythrophagocytosis while overexpression of NR4a1 restored their phagocytic activity (Fig 8C).

To further test the hypothesis that targeting NR4a1 mitigates the effects of HSD on macrophage reparative functions *in vivo*, we pharmacologically activated NR4a1 in HSD mice using celastrol, a triterpenoid quinine methide extracted from the plant *Tripterygium wilfordii* (Fig 8D). Administration of celastrol increased Ly6C^{low} monocytes and decreased the Ly6C^{hi} proinflammatory monocytes in the blood samples of HSD mice at day 4 post-ICH (Fig EV5A). During the recovery phase, celastrol improved neurobehavioral performance in HSD mice on day 7 post-ICH (Fig 8E). Celastrol also significantly reduced the hematoma volume and increased the number of NeuN^{+} neurons in the perihematomal region in HSD mice (Fig 8F). These changes are likely to be mediated through enhanced reparative macrophage function, as higher numbers of MAC2^{+}IBA1^{+} macrophages were observed in HSD mice receiving celastrol (Fig 8G). To confirm that the observed effects with celastrol treatment were indeed mediated through NR4a1, we treated *NR4a1* cKO mice with celastrol after ICH induction and evaluated hematoma clearance and alternative activation of MDMs in the hemorrhagic brains (Fig EV5B). At day 7 post-ICH, no difference in hematoma volume and the percentage of reparative MDMs (ARG1^{+}, CD36^{+}, CD206^{+}, and HO-1^{+}) was observed between vehicle- and celastrol-treated *NR4a1* cKO ICH mice (Fig EV5C and D). This suggests that celastrol acts through NR4a1 to exert its beneficial effects in the HSD ICH brains. Collectively, these results identify NR4a1 as a key mediator of the HSD-induced reduction in the reparative macrophage phenotype and suggest targeting NR4a1 may effectively mitigate the HSD-induced delay in ICH recovery.

Discussion

Beyond its well-established association with hypertension, salt is increasingly recognized as a modulator of sterile inflammatory diseases (Kleinewietfeld *et al*, 2013; Wilck *et al*, 2019). This work provides the first demonstration that HSD delays recovery from both ICH and cerebral ischemia. Mechanistically, HSD induces innate immune memory in HSPCs and macrophages, which adversely affects long-term brain repair after stroke, even after withdrawal or reduction in salt prior to the injury. This HSD-initiated immunological priming and training of BM progenitor cells, which is likely achieved via metabolic reprogramming (Divangahi *et al*, 2021), is retained in the mature progeny of BM progenitor cells and leads to reduced alternative activation of macrophages in inflamed tissues. This further impedes tissue and neurobehavioral recovery and exacerbates neuronal loss and astrogliosis during the chronic stage of stroke. Although withdrawal from HSD restored systemic inflammation and myelopoiesis to homeostatic levels, it failed to "erase" the memory as macrophages continued to exhibit diminished reparative functions. Likewise, BMDMs retained reduced reparative activation despite being removed from their original *in vivo* niche for 7 days, a rest period that was suggested to demonstrate innate immune memory in a culture system (Divangahi *et al*, 2021). Notably, the same immunological memory could be "transferred" to previously healthy mice and persisted for up to 8 weeks post-bone marrow transplantation. The mature myeloid progeny of transplanted HSD BM cells exhibited reduced reparative functions, which in turn negatively affected brain recovery. Finally, loss of NR4a1 alone in macrophages recapitulated the phenotype induced by HSD and could be rescued by pharmacological activation of NR4a1 using celastrol. Together, these results establish a mechanistic link between HSD and adverse long-term stroke recovery and unveil a potential therapeutic target to alleviate HSD-induced noxious innate immune responses and promote resolution of inflammation after sterile brain injuries.

Innate immune memory improves protection against viral and fungal re-infections. However, innate immune reprogramming also has deleterious effects on several chronic inflammatory and autoimmune diseases (Netea *et al*, 2020). The contribution of innate immune memory after vaccination and microbial stimuli is well studied (Bekkering *et al*, 2018; Kaufmann *et al*, 2018; Mitroulis *et al*, 2018; Wendeln *et al*, 2018); however, few studies have examined how dietary patterns precipitate innate immune memory or how maladaptive innate immune responses affect CNS disease outcomes. The typical Western diet induces systemic inflammation, which triggers formation of innate immune memory in hematopoietic precursor cells (Christ *et al*, 2018). Similarly, we observed systemic inflammation in our HSD mice, as indicated by increased inflammatory monocytes (CD45^{hi}CD11b^{+}Ly6C^{hi}), as well as transcriptional and metabolic property changes in BM. Furthermore, we observed reduced OXPHOS in BM cells from HSD mice, consistent with clinical observations that a high-salt intake affects mitochondrial energetics and reduces OXPHOS and ATP production in human PBMCs (Geisberger *et al*, 2021). In fact, HSPCs from HSD mice exhibited enrichment of pathways associated with glycolysis, the inflammasome pathway, cholesterol biosynthesis, NRF2-mediated oxidative stress responses, and toll-like receptor signaling, all of which constitute the cardinal features of innate immune memory (Zhang *et al*, 2021). Our *in vivo* findings from HSD BM chimeric mice further corroborate our hypothesis that HSD triggers innate immune memory in the BM niche. Interestingly, while both Western and high-salt diets induce systemic inflammation and ultimately immune memory in HSPCs (Christ *et al*, 2018), these processes occur by distinct mechanistic routes. Future studies are needed to understand how different environmental factors, including diets, program innate immune memory.

Enhanced proinflammatory responses upon encountering secondary insults are a core signature of innate immune memory in monocytes and macrophages (Dominguez-Andres *et al*, 2022). Interestingly, HSD-induced immunological memory did not affect the initial proinflammatory responses of brain-infiltrating MDMs during the acute phase of ICH. Instead, the MDM transition to an alternatively activated phenotype was suppressed—and their reparative function diminished—during the chronic recovery phase. Recent studies suggest innate immune memory has impacts beyond classical proinflammatory responses, as it can also alter macrophage alternative activation and profibrotic properties as well as triggering type 2 immune responses (Jeljeli *et al*, 2019; Cunningham *et al*, 2021; Hartung & Esser-von Bieren, 2022). Collectively, findings from our study and others offer an exciting direction to investigate the heterogeneity of innate immune memory on disease pathogenesis and outcomes.

Our findings that the NR4a family mediates HSD-induced innate immune memory are complemented by recent studies

© 2023 The Authors

demonstrating the necessity of NR4a for maintenance of HSPC quiescence. For example, deletion of NR4a1 and NR4a3 leads to chronic hyperproliferation of HSPCs (Land *et al*, 2015; Freire & Conneely, 2018). NR4a1 is also required for the development of Ly6C^{low} monocytes in BM and the transition from Ly6C^{hi} to Ly6C^{low} in inflamed tissues, as NR4a1-deficient mice lack Ly6C^{low} surveillance monocytes in the peripheral blood and Ly6C^{low} macrophages in the infarcted myocardium (Hanna *et al*, 2011; Carlin *et al*, 2013; Hilgendorf *et al*, 2014). Similarly, HSD induced downregulation of NR4a1 and NR4a3 in HSPCs and increased myelopoiesis in BM and circulating Ly6C^{hi} monocytes. Our results also agree with previous findings that NR4a1 is a regulator of inflammatory disease and that knockdown or deletion of NR4a1 in macrophages results in lower OXPHOS and reduced alternatively activated properties (Hanna *et al*, 2012; Koenis *et al*, 2018). Our results unveil a previously unexplored role for NR4As in regulation of macrophage function after ICH (and possibly other HSD-related cardiovascular diseases) and improve our understanding of NR4a-mediated regulatory pathways under sterile inflammatory conditions. However, since endogenous ligands for NR4As have not been identified, the mechanisms by which HSD alters NR4a1 expression await further elucidation.

Macrophages—including microglia and MDMs—are the most abundant myeloid population in the stroke brain and play key roles in brain tissue repair (Chang *et al*, 2018, 2021). Viral and bacterial components can trigger immune memory in the brain and alveolar tissue-resident macrophages (Wendeln *et al*, 2018; Yao *et al*, 2018). In the CNS, LPS stimulation induces immune memory in microglia and leads to persistent modifications of stroke pathology (Wendeln *et al*, 2018). Microglia are long-lived brain-resident macrophages, thus represent ideal candidates for building immune memory of chronic dietary-related stimulators. To our surprise, we did not detect phenotypic changes in microglia after HSD. While we cannot exclude the possibility that our experimental strategies were insufficient to characterize the complete responses of microglia to HSD, no study has yet reported that either sterile inflammatory ligands or diet-induced systemic inflammation induces immunological memory in tissue-resident macrophages (Dominguez-Andres *et al*, 2022). Given that the effects of HSD on microglia and macrophage polarization remain debatable (Amara *et al*, 2016; Heras-Garvin *et al*, 2020; Zhang *et al*, 2020), future work evaluating microglial responses through high-throughput sequencing could be informative. Investigation of whether innate immune memory can be triggered in different tissue-resident macrophage populations after HSD and the contribution of these populations to disease progression represents a fascinating area of research.

HSD is strongly linked to poorer health outcomes and increased risk of chronic cardiovascular diseases; therefore, understanding the mechanisms that mediate HSD-induced unfavorable immune responses in disease is imperative to develop effective therapeutics. Previous studies have implied a link between HSD and reduced macrophage alternative activation (Binger *et al*, 2015; Zhang *et al*, 2015), although these experiments focused on macrophage responses to acute sodium changes *in vitro*. Our work further supports the idea that high salt primes and impacts macrophage function, as differentiation of healthy BM cells in a high-sodium environment hampered BMDM-reparative gene expression after thrombin stimulation. Our evidence of how HSD induces immunological memory in BM progenitors and persistently modifies the macrophage phenotype elucidates a previously unknown mechanism that underlies the persistent detrimental effects of HSD in inflammatory tissue injury. However, despite the continuing efforts to reduce salt intake at the population level, daily sodium intakes per capita still far exceed the recommended amounts (He *et al*, 2021), which has prompted investigation into new therapeutic strategies for high-salt-related cardiovascular diseases. We found high dietary salt consistently downregulated *NR4a1* and *NR4a3* in HSPCs, regardless of ICH induction. Moreover, HSD-induced transcriptional reprogramming enriched pathways associated with NFAT-regulated immune responses, which further supports the idea that downregulation of NR4a is salt specific (He *et al*, 2020). Although the endogenous ligands for NR4As are unknown, several compounds can activate NR4a1—including celastrol, a natural compound found in the roots of the thunder god vine (Safe *et al*, 2021). As expected, HSD mice treated with celastrol exhibited reduced Ly6C^{hi} and increased Ly6C^{lo} monocyte populations in peripheral blood after ICH, in parallel with increased reparative macrophages and improved neurobehavioral outcomes. As celastrol exerts pluripotent effects in several diseases (Safe *et al*, 2021), we cannot entirely exclude the possibility of an off-target effect on other immune populations. However, the anti-inflammatory and metabolic modulatory properties of celastrol make it an ideal candidate to counteract HSD-induced maladaptive innate immune memory and highlight the therapeutic potential of targeting NR4As in HSD-associated diseases. The efficacy of NR4a manipulation and celastrol to modulate innate immune memory in HSPCs especially warrants further investigation. Collectively, targeting maladaptive innate immune memory may represent a practical and druggable approach to improve HSD-associated neurological symptoms, such as stroke.

Nevertheless, a few caveats and questions should be kept in mind. First, the enrichment of sodium-related pathways, such as NFAT, in HSPCs suggests an HSD-specific response. However, the sodium-sensing receptors and intracellular signaling pathways activated by HSD in BM progenitor cells remain elusive. Additionally, the precise mechanisms underlying downregulation of NR4As and metabolic rewiring in HSPCs remain to be explored. Investigating whether NR4As-mediated trained immunity can be found following other microbial and non-microbial stimuli will also be interesting. Second, previous studies have shown that accumulation of metabolites such as fumarate, succinate, and mevalonate leads to epigenetic modifications at loci responsible for innate immune memory (Dominguez-Andres *et al*, 2022). Future studies can benefit from identifying the crucial metabolites and epigenetic changes that mediate HSD-induced immunological memory. Third, future work is needed to determine how rapidly HSD induces systemic changes and innate immune memory as well as how long these changes persist after salt reduction. Fourth, while we specifically focused on MDMs in this study, but of course, the contributions from other myeloid populations to stroke outcomes await further investigation as high salt is reported to also impact the functions of neutrophils, myeloid-derived suppressor cells, and dendritic cells in several other diseases (Ferguson *et al*, 2019; He *et al*, 2020; Jobin *et al*, 2020). Fifth, *in vitro* stimuli such as thrombin and high-sodium media used in this study may not be able to fully reflect the complexity of the *in vivo* environment. Finally, the translational potential of targeting NR4As to treat high salt-associated cardio- and cerebrovascular diseases needs to be confirmed.

© 2023 The Authors

Materials and Methods

Animals

NR4a1$^{fl/fl}$; *NR4a2*$^{fl/fl}$; *NR4a3*$^{-/-}$ mice were kindly provided by Dr. Akihiko Yoshimura, Keio University School of Medicine (Sekiya *et al*, 2013). *LysM*$^{Cre/+}$ mice (Jax no. 004781) were obtained from the Jackson Laboratory and backcrossed for > 9 generations to a C57BL/6J (Jax no. 000664) background. To broadly delete NR4a family members from macrophages, *NR4a1*$^{fl/fl}$; *NR4a2*$^{fl/fl}$; *NR4a3*$^{-/-}$ mice were crossed with *LysM*$^{Cre/+}$ mice (*NR4a* TKO); *NR4a1*$^{fl/fl}$; *NR4a2*$^{fl/fl}$; *NR4a3*$^{+/+}$ littermates were used as controls. To delete NR4a1 in macrophages, *NR4a1*$^{fl/fl}$; *NR4a2*$^{fl/fl}$; *NR4a3*$^{-/-}$ mice were crossed with C57BL/6J mice to generate *NR4a1*$^{fl/fl}$ mice, which were subsequently crossed with *LysM*$^{Cre/+}$ mice (*NR4a1* cKO); *NR4a1*$^{fl/fl}$ mice were used as controls. Mice were used at the ages specified below; only males were used. All experiments were performed in accordance with the *Guide for the Care and Use of Laboratory Animals* from the National Institutes of Health and Animal Research: Reporting *In Vivo* Experiments guidelines, and approved by the Institutional Animal Care and Use Committee of the National Taiwan University College of Medicine (approval number: 20190326). All effort was made to minimize animal suffering and the number of animals used. Mice were housed in a temperature and humidity-controlled facility under a 12 h light/dark cycle. A total of 472 mice were used. Five collagenase ICH mice died after surgery due to inherent preclinical ICH mortality. Six chimeric mice and one *NR4a1* cKO mice were excluded from statistical analysis due to either failed surgery (two ND chimeras and one *NR4a1* cKO mice; blood ICH model) or ethical considerations (one ND chimera and three HSD chimeras; weight loss > 15% of pre-surgery weight). Animals were randomly allocated to experimental groups using a coin-toss randomization approach. Behavioral tests (J.S. Lin) and all other analyses (T.-Y. Lin, D.J., W.-R. Chen, X.-Y. Zhang, and C.-H. Chen) were performed by investigators blinded to the experimental groups and treatment assignments.

ICH models

Blood injection model

ICH was induced by autologous blood injection, as previously described (Chang *et al*, 2018; Tsai *et al*, 2022). Briefly, 25 μl autologous blood collected from the submandibular vein was injected 0.2 mm anteriorly, 2.3 mm laterally, and 3.0 mm deep relative to the bregma. The craniotomy was sealed with bone wax and the scalp was closed with tissue adhesive (3M Vetbond). Rectal temperature was monitored and maintained at 37.0 ± 0.5°C (Stoelting Rodent Warmer X2) throughout the procedure. After surgery, body temperature was maintained using a heat lamp until the animals recovered. All ICH surgeries were performed by blood injection, unless noted otherwise.

Collagenase injection model

Larger ICH and more severe functional deficits were modeled using collagenase injection, as previously described (Chang *et al*, 2018; Tsai *et al*, 2022). Mice underwent the same pre-operative and post-operative procedures described above; however, ICH was induced by injecting 0.5 μl of 0.4 U type VII-S collagenase (from *Clostridium histolyticum*, Sigma-Aldrich) at 0.1 μl/min into the right striatum.

tMCAO model

A transient middle cerebral artery occlusion (tMCAO) was performed to induce focal cerebral ischemia/reperfusion, as previously described (Wu *et al*, 2016). Briefly, the right middle cerebral artery was ligated reversibly with 10-0 nylon suture, and the right common carotid artery was also occluded reversibly with aneurysm clips. The cerebral blood supply was restored after 40 min of MCAO. Rectal temperature was monitored and maintained at 37.0 ± 0.5°C (Stoelting Rodent Warmer X2) throughout the procedure and during recovery.

Dietary regimens

Mice (4- to 6-weeks-old) received either normal chow (ND, 0.5% NaCl; D10001, Research Diet Inc.) and tap water *ad libitum* or high-sodium chow (HSD, 8% NaCl; D09022503, Research Diet Inc.) and tap water containing 1% NaCl *ad libitum* for 2–8 weeks. To study the effects of HSD after diet reversal, 6-week-old mice received either ND or HSD for 8 weeks before switching to ND for 1 week.

Celastrol treatment

ICH-operated mice were randomly assigned to the vehicle or celastrol groups. The celastrol group was *i.p.* injected with 1 mg/kg celastrol (Sigma-Aldrich) dissolved in 50% dimethylsulfoxide (DMSO) immediately after ICH and for 3 or 6 consecutive days following ICH. The vehicle group was *i.p.* injected with 50% DMSO.

Physiological measurements

Body weight, food intake, water intake, sodium intake (calculated from food and water intake), blood pressure, and heart rate measurements were taken weekly for 8 weeks. Food, water, and sodium intakes were monitored for 1 week after ICH induction. Blood pressure and heart rate were measured using a tail-cuff Blood Pressure Monitor (Muromachi Kikai, MK-2000ST).

Tissue collection and histology

Samples for immunofluorescence and immunohistochemistry were prepared as previously described (Chang *et al*, 2020; Tsai *et al*, 2022). Briefly, mice were anesthetized with isoflurane, euthanized by transcardial perfusion with ice-cold 0.1 M PBS followed by 4% paraformaldehyde (PFA), and the brain was removed and kept in 4% PFA overnight. Following cryoprotection in 30% sucrose/PBS, samples were embedded in OCT, coronally sectioned at 10 μm on a cryostat (Leica), and incubated with blocking solution (8% normal goat serum [NGS], 0.1% Tween 20, and 0.1% Triton X-100 in PBS) for 1 h at room temperature (RT), followed by primary antibodies [anti-NeuN (Millipore), anti-GFAP (Thermo), anti-Iba1(Wako), anti-MAC2 (Biolegend), anti-CD206 (Bio-rad), anti-MAP2 (Millipore), anti-ARG1 (Abcam), and anti-Ly76 (Abcam)] in dilution buffer (5% NGS, 0.1% Tween 20, and 0.1% Triton X-100 in PBS) overnight at 4°C. After three 5 min washes with PBS, the corresponding secondary antibodies in dilution buffer were applied for 2 h at RT, then the brain sections were washed again and mounted in VECTASHIELD mounting medium (Vector Laboratories). For cresyl violet histology,

© 2023 The Authors

coronal brain sections were incubated in 0.1% cresyl violet acetate at RT for 3 h, rinsed twice with distilled water, dehydrated in graded alcohols, and mounted in dibutylphthalate polystyrene xylene (DPX). Images of sections were acquired using a Zeiss Axio Imager M1 with a 20×/0.8 NA objective or an Olympus IX81 fluorescence microscope with 1.25×/0.04 NA, 20×/0.8 NA, and 40×/0.75 NA objectives at a resolution of 1,360 × 1,024 pixels and analyzed using ImageJ.

Preparation of cell suspensions and cell sorting

Microglia were isolated as previously described (Chang *et al*, 2020, 2021). Briefly, mice were euthanized and transcardially perfused with 40 ml of cold PBS, and 4 mm fresh coronal perihematomal brain sections were mechanistically and enzymatically digested with 10 mg/ml DNase I (Roche) and 1 mg/ml collagenase/dispase (Roche) at 37°C for 45 min. Cell suspensions were passed through a 40 μm strainer and underlaid with a 30%/70% Percoll gradient (GE healthcare) to enrich leukocytes and remove myelin debris. Leukocytes were washed, incubated with antibodies for cell surface markers (CD45 [30-F11], CD11b [M1/70], CD3 [17A2], CD19 [6D5], Ly6G [1A8], and TMEM119 [106-6]) for 20 min at 4°C, washed with HBSS buffer, stained with a viability dye prepared in HBSS buffer (LIVE/DEAD fixable Dead Cell Stain Kit; Invitrogen) for 20 min, and washed with FACS buffer. Microglia were identified as LIVE/DEAD$^-$CD3$^-$CD19$^-$Ly6G$^-$CD11b$^+$CD45^{int}TMEM119$^+$ cells and sorted on a FACSAria (BD biosciences) for further RNA isolation and gene expression analysis. To isolate hematopoietic stem and progenitor cells (HSPCs), bone marrow was flushed from femurs and tibias with cold PBS. Red blood cells were lysed with RBC Lysing Buffer Hybri-Max (Sigma), followed by staining with antibodies against lineage-committed cells (CD3e [145-2C11], CD8a [53–6.7], CD19 [6D5], Gr1 [RB6-8C5], B220 [RA3-6B2], CD11b [M1/70], I-A/I-E [M5/114.15.2], TER-119 [TER-119], and NK1.1 [PK136]) and cell surface markers (CD127 [A7R34], c-kit (CD117) [2B8], Sca-1 [E13-161.7], CD34 [RAM34], and CD16/32 [93]). GMPs were identified as Lin$^-$CD127$^-$c-kit$^+$ Sca-1$^-$CD16/32$^+$CD34^{int} cells. HSPCs were identified as Lin$^-$CD127$^-$c-kit$^+$Sca-1$^+$ cells and sorted on a FACSAria (BD biosciences) for further RNA isolation and RNA sequencing.

Flow cytometry

Leukocytes from brain tissues were prepared as described above and stained with antibodies for cell surface markers (CD45 [30-F11], CD11b [M1/70], Ly6C [HK1/4], Ly6G [1A8], CD36 [CRF D-2712], MHCII [M5/114.15.2], CD86 [GL-1], CD206 [C068C2], MerTK [DS5MMER], CD3e [17A3], and CD19 [6D5]) at 4°C for 20 min, washed with HBSS buffer, stained with a viability dye as described above for 20 min, and washed with FACS buffer. For intracellular staining, leukocytes were incubated with brefeldin A for 4 h prior to staining. After LIVE/DEAD staining, cells were then fixed and permeabilized with BD CytoFix/CytoPerm solution (BD Biosciences) for 20 min at 4°C, washed, and incubated with TNF [MP6-XT22], IL-1α [ALF-161], IL-6 [MP5-20F3], HO-1 [HO-1-2], or Arginase 1 [A1exF5] antibody for 30 min at 4°C. Blood samples were collected via cardia puncture into heparin-coated tubes, immediately placed on ice, red blood cells were lysed using RBC Lysing Buffer Hybri-Max (Sigma), followed by antibody staining with surface markers (CD45 [30-F11], CD11b [M1/70], Ly6C [HK1/4], Ly6G [1A8], CD19 [6D5], and CD3 [145-2C11]) for 20 min at 4°C, washed with HBSS buffer, stained with a viability dye for 20 min, and washed with FACS buffer as described above. Control samples were taken from naive brains matching the 4 mm coronal sections at the perihematomal region and peripheral blood of intact mice. Gates were set based on fluorescence-minus-one (FMO) controls. Data were collected on a BD LSRFortessa and analyzed using the FlowJo software (v10.5.4).

RT-qPCR

Total RNA was isolated using the miRNeasy Micro Kit (Qiagen) according to the manufacturer's instructions. First-strand cDNA was synthesized from 100 ng RNA with SuperScript VILO cDNA Synthesis Kit (Invitrogen) and qRT-PCR was performed using the StepOnePlus Real-Time PCR System (Applied Biosystems). Primers were obtained from Thermo Fisher Scientific: *Tnf* (Mm00443258_m1), *Tspo* (Mm00437828_m1), *Trem2* (Mm04209424_g1), *Retnla* (Mm00 44 5109_m1), *Mrc1* (Mm01329362_m1), *Arg1* (Mm00443258_m1), *Hdac1* (Mm02745760_g1), *Hdac2* (Mm00515108_m1), *Tmem119* (Mm00525305_m1) and *Apoe* (Mm01307193_g1). *Gapdh* (Mm0 99999915_g1), or *r18s* (Mm03928990_g1) was used as the endogenous control. The cycle time of target genes was normalized to that of *Gapdh* or *r18s* in the same sample and mRNA expression levels were calculated using the delta delta CT (ΔΔCT) method, unless noted otherwise.

RNA-seq

HSPCs were sorted from the bone marrow of ND and HSD mice with or without ICH induction, as described above. Total RNA was isolated using the miRNeasy Micro Kit (QIAGEN) and RNA quality was assessed with Qsep100 (BiOptic Inc.) and Qubit 3 fluorometer (Thermo Fisher Scientific). RNA sequencing libraries were generated using the SMART-Seq Stranded Kit (Takara Bio) according to the manufacturer's instructions. Sequencing was performed on an Illumina NovaSeq 6,000 sequencer (Illumina), resulting in 15 to 50 million 150-bp pair-end sequencing reads per sample. Library construction and sequencing were conducted by the sequencing core at the National Taiwan University Hospital.

Analysis of RNA-seq data

Sequencing reads were aligned to the mouse reference (mm10) using Rsubread aligner (v.1.32.4) and mapped reads were quantified at the gene level using featureCounts (Liao *et al*, 2014). Only genes with counts above 10 counts per million (CPM) in at least three samples were analyzed. The trimmed mean of M-values (TMM) method was performed for normalization using edgeR (v3.24.3) (Robinson *et al*, 2010); 289 differentially expressed genes (DEGs; P-value < 0.05; $\log_2 FC \geq 1$ or ≤ -1) were identified in HSD HSPCs versus ND HSPCs. The DEG heatmap was generated using Heatmapper software (Babicki *et al*, 2016). Principal component analysis (PCA) was performed using scikit-learn 1.1.2 to generate a 3D PCA plot. Transcription factors were presented using a volcano plot generated by Prism 9. Gene set enrichment analysis (GSEA; v4.1.0) was conducted using the GSEA software (Broad Institute) and the hallmark gene sets (v7.5) of the Molecular Signatures Database

© 2023 The Authors

(MsigDB) using pre-ranked permutation numbers of 1,000. The sum of the weighted enrichment score was applied to generate the enrichment score curve. Pathway analysis was performed using Ingenuity Pathway Analysis (vQ4 2020, QIAGEN).

Thioglycollate-induced peritoneal inflammation

Mice (6- to 8-weeks-old) were i.p. injected with 4% *wt/vol* thioglycollate (in ddH_2O) to induce peritonitis, as previously described (Bosurgi *et al*, 2017). Three or seven days later, animals were euthanized, the peritoneal cavity was washed with 5 ml of cold PBS, and peritoneal cells were seeded into a 10 cm culture dish in complete RPMI medium (10% HI-FBS, 1% MEM NEAA, 1% sodium pyruvate, 1% penicillin/streptomycin, 1% L-glutamine, and 0.1% 2-ME, all from Gibco) and incubated at 37°C for 4 h. Non-adherent cells were removed by gently washing twice with pre-warmed PBS. Peritoneal macrophages were collected and processed for flow cytometry analysis. For the *in vitro* erythrophagocytosis assay detailed below, the peritoneal cells were isolated 7 days after injection.

Bone marrow-derived macrophages (BMDMs)

BMDMs were generated from 4- to 6-week-old wild-type C57BL/6 and *NR4a1* cKO mice, as previously described (Chang *et al*, 2018, 2020). Briefly, bone marrow was isolated from the femurs and tibias, and adherent stromal cells were depleted by culturing the harvested bone marrow at 37°C overnight in α-minimal essential medium (α-MEM, Bio Whittaker Lonza) supplemented with 10% FBS, 100 U/ml penicillin plus 100 mg/ml streptomycin, and 2 mM L-glutamine (all Gibco). Non-adherent mononuclear cells were collected, red blood cells were lysed with RBC Lysing Buffer Hybri-Max (Sigma), and 3×10^5 cells were cultured in a 6-well plate for 7 days in α-MEM with 50 ng/mL recombinant murine M-CSF (R&D Systems), yielding BMDMs. BMDMs were stimulated with 10 U/ml thrombin (Sigma Aldrich) or 20 ng/ml IL-4 under serum-free conditions for 3 or 6 h. For the *in vitro* erythrophagocytosis assay, BMDMs were differentiated from enriched bone marrow progenitor cells for 7 days under normal isotonic conditions (139 mM) or in the presence of an additional 40 mM of sodium chloride (179 mM) after transducing with either control virus or NR4a1-overexpressing virus, as detailed in the lentiviral transduction section.

In vitro erythrophagocytosis assay

Peritoneal cells (2×10^6) were collected as described above, cultured on round 12 mm glass coverslips in a 24-well plate for 24 h, and non-adherent cells were removed by gently washing twice with warm PBS. Adherent peritoneal macrophages were maintained for 24 h in complete RPMI medium before the erythrophagocytosis assay (Chang *et al*, 2018, 2020). Briefly, red blood cells (RBCs) were isolated from whole blood of donor mice, heat-shocked at 56°C for 6 min to induce phosphatidylserine expression, and labeled using the PKH26 Red Fluorescent Cell Linker Kit (Sigma-Aldrich). Peritoneal macrophages were incubated with thrombin (10 U/ml) under serum-free conditions for 3 h, subsequently fed with PKH26-labeled RBCs (1:20) for 30 min at 37°C, and unengulfed erythrocytes were removed by washing with PBS. For BMDMs, the cells were treated following the same procedure as described above without thrombin stimulation. Macrophages were fixed in 4% paraformaldehyde (PFA), stained with FITC-labeled rat anti-mouse CD11b (M1/70) antibody, and mounted on a glass slide. Engulfed erythrocytes were observed using an Olympus IX81 Inverted Fluorescence Microscope; images were captured from three independent fields of view. The phagocytosis index was calculated as the mean fluorescence intensity of engulfed erythrocytes divided by the total number of macrophages in each image.

In vivo erythrophagocytosis assay

The PKH-26-labeled RBCs were suspended in autologous plasma at a 1:4 ratio of RBCs and plasma (20% hematocrit). Twenty-five microliters of labeled RBCs in plasma were injected into the brains of ND and HSD mice as in the blood injection ICH model. Mice were then sacrificed at 7 days after surgery and samples were prepared and analyzed by flow cytometry as described above. The LIVE/DEAD$^-$CD45^{hi}CD11b$^+$Ly6G$^-$CD3e$^-$CD19$^-$Ly6C$^+$PKH-RBC$^+$population was defined as MDMs that had engulfed RBCs.

Metabolic assays of BM cells and BMDMs

Oxygen consumption rate, basal respiration, maximal respiration, ATP production, extracellular acidification rate, and glycolytic capacity in BM cells or BMDMs were measured using the Seahorse XFe24 Analyzer (Agilent), as previously described (Koenis *et al*, 2018). Briefly, BM cells were plated at 2×10^5 cells/well, and BMDMs were plated at 2.5×10^4 cells per well into XFe 24-well culture microplates coated with Cell-Tak (BD Biosciences) and incubated for 1 h in a 37°C non-CO_2 incubator, then assayed using the Seahorse XF Cell Mito Street Test kit (OCR; oxygen consumption rate) and the Seahorse XF Glycolysis Stress Test kit (ECAR; extracellular acidification rate) according to the manufacturer's protocol (Agilent). The oxygen consumption rate was measured under basal conditions and in response to sequential titration to 1 μM oligomycin, 2 μM carbonyl cyanide-4-(trifluoromethoxy) phenylhydrazone (FCCP), and 0.5 μM rotenone/actinomycin A dissolved in Seahorse XF medium (Agilent). The glycolytic rate was assessed by sequential injections with glucose (100 mM), oligomycin (10 μM), and 2-deoxy-D-glucose (2-DG; 100 mM). Data were generated using Seahorse Wave Controller Software v2.2 (Agilent).

Bone marrow chimeras

Six- to eight-week-old wild-type C57BL/6 recipient mice and 8-week HSD-fed recipient mice were lethally irradiated with 900 cGy twice at an interval of 4 h, followed by randomly assigned retro-orbital injection of 10^6 donor BM cells (in 100 μl PBS) from ND or HSD donor mice. Chimeras were maintained on sulfamethoxazole/trimethoprim antibiotics in drinking water beginning 2 days before and continuing for 1 week following irradiation and pair housed as previously described (Chang *et al*, 2018). Chimeras were allowed to recover for 8 weeks before ICH surgery.

Neurobehavioral tests

Investigators blinded to the group assignments evaluated functional and neurological deficits using the cylinder test, neurological deficit scoring system, hindlimb abduction test, and corner turn test were

© 2023 The Authors

performed as previously described (Clark *et al*, 1998; Schaar *et al*, 2010; Chang *et al*, 2017, 2018, 2020).

Cylinder test
Mice were placed in a clear glass cylinder and allowed to move freely. The first forelimb used, defined as placement of the entire palm on the cylinder wall while rearing, was scored for a total of 20 contacts for each animal. The laterality index was calculated as (right − left)/(right + left + both); a higher positive value indicates more severe hemiparesis and a score of 0 represents equal use of the forelimbs.

Neurologic deficit scoring system
A range of parameters were evaluated, including body symmetry, forelimb symmetry, climbing, gait, circling behavior, and compulsory circling. Each test was scored from 0 to 4, with a maximum deficit score of 24.

Hindlimb abduction test
Mice were placed on the edge of a platform and the hind paw was gently pulled down away from the edge. Intact animals will rapidly retrieve and replace their hindlimb on the benchtop. A total of 20 trials per day was recorded and the percentage of successful placing responses was quantified.

Corner turn test
Mice were allowed to proceed into a 30° corner and freely turn either left or right to exit the corner. The choice of direction was recorded during 10 trials. The percentage of right turns was calculated. Mice that have suffered a stroke preferentially leave toward the non-impaired (right) body side.

Lentivirus construction and production

Monomeric GFP (mGFP) or mNR4a1-mEGFP-coding DNA synthesized by Protech Technology Enterprise were subcloned into the EcoRI and XbaI sites of the pLVX-IRES-Puro backbone to generate pLVX-mGFP-IRES-Puro or pLVX-mNR4a1-mEGFP-IRES-Puro, respectively. HEK293T cells were plated at 8×10^6 cells per 15 cm dish 1 day before transfection and transfected with 11.76 μg of packaging vectors (5.88 μg of pMD2.G [Addgene #12259] and 5.88 μg psPAX2 [Addgene #12260]) and 5.88 μg of vector plasmids (pLVX-mGFP-IRES-Puro or pLVX-mNr4a1-mEGFP-IRES-Puro). Viral vector-containing supernatants were harvested over 72 h, filtered through a 0.45 μm filter (Millipore), concentrated by centrifugation at 32,000 *g* for 2 h at 4°C, and stored at −80°C until experiments. Vector stocks were titrated on HEK293T cells by FACS; vector titers are expressed as EGFP transduction units per milliliter.

Lentiviral transduction

Bone marrow progenitor cells were enriched using the Direct Lineage Cell Depletion Kit (Miltenyi Biotec), seeded into a 12-well plate at 5×10^5 cells/well, incubated with 800 μl RPMI medium (Gibco) for 30 min at 37°C, and transduced with either 10 μl control virus (9×10^9 TU/ml) or 20 μl NR4a1 virus (1×10^9 TU/ml) for 24 h at 37°C. The following day, transduced cells were resuspended in fresh RPMI medium supplemented with 50 ng/ml M-CSF and allowed to differentiate under normal isotonic conditions (139 mM) or in the presence of an additional 40 mM of sodium chloride (179 mM).

Histological quantification

Hematoma volume, neuron number, and astrogliosis
For cryostat sections, hematoma areas were defined as the Ly76$^+$ area and measured using ImageJ, as previously described (Tsai *et al*, 2022). Hematoma volume (mm^3) was calculated as the sum of the hematoma area multiplied by the interslice distance (210 μm). For fresh coronal brain sections, 1 mm coronal sections were collected using a brain slicer matrix (ASI Instruments) and sharp razor blades based on our previous protocol (Chang *et al*, 2020). Images of brain slices were digitalized by a scanner (600 dpi) and analyzed with ImageJ (NIH) by experimenters blinded to the experimental group. The volume of hematoma was calculated as the summation of the hematoma areas multiplied by the interslice distance (1 mm) as previously described (Chang *et al*, 2018). Three independent fields of view (696.3 μm × 524.3 μm) of the perihematomal region were captured for each animal to quantify neuron number and astrogliosis. NeuN$^+$DAPI$^+$ and GFAP$^+$DAPI$^+$ cells were counted in each image and expressed as cell counts per mm^2.

Tissue loss and neuronal damage
Infarct area, infarct volume, and neuron damage area were measured as previously described. Briefly, 10-μm-thick coronal brain sections were stained with 0.1% cresyl violet acetate solution for 3 h at room temperature, washed twice with distilled water, dehydrated in an ethanol gradient, mounted in dibutylphthalate polystyrene xylene (DPX), and imaged using an Olympus IX81 microscope (Olympus) with a 1.25× objective. The infarct area was determined as the unstained area and infarct volume was calculated by multiplying the infarct area by the interslice distance (800 μm). Neuronal damage was defined as the loss of MAP-2 immunofluorescence and the area of neuronal damage was measured in brain sections of the hematoma core +1.4 mm from the bregma.

Reparative macrophage number
Three independent fields of view (696.3 μm × 524.3 μm) were captured in the perihematomal region of each animal. The numbers of cells co-expressing IBA1, DAPI, and reparative markers of interest (CD206, MAC2, and Arg1) were counted in each field, divided by the total number of reparative macrophages in each view by the image area, and expressed as cell counts per mm^2.

Statistical analysis

No statistical methods were used to predetermine the sample size; however, the sample sizes in this study are similar to previous publications (Bosurgi *et al*, 2017; Shi *et al*, 2021). Data are expressed as mean ± SEM. Individual mouse or biological replicates are represented as circles in figures. GraphPad Prism software (v9.4.1) was used for statistical analyses. The unpaired Student's *t*-test was used to compare two groups; one-way or two-way ANOVA with *post hoc* Bonferroni test was employed for multiple-group comparisons.

© 2023 The Authors

Details of statistical analyses including sample numbers (n) are included in the respective figure legends. $P < 0.05$ was considered statistically significant.

Study approval

All murine procedures were approved by the National Taiwan University College of Medicine Animal Care and Use Committee (approval number: 20190326) and were performed in strict compliance with the National Institutes of Health (NIH) Guide for the Care and Use of Laboratory Animals.

Data availability

The datasets produced in this study are available in the following databases:

- RNA-Seq data: Gene Expression Omnibus GSE214728 (https://www.ncbi.nlm.nih.gov/geo/query/acc.cgi?acc=GSE214728).
- Image data: Biostudies S-BIAD829 (https://www.ebi.ac.uk/biostudies/bioimages/studies/S-BIAD829).

Expanded View for this article is available online.

Acknowledgements
We thank Dr. Song-Kun Shyue and Dr. Teng-Nan Lin for providing laboratory resources; Dr. Ping-Chih Ho for providing insightful comments; Ms. Zi-Ting Kuo for technical support; the flow cytometric analyzing and sorting core and the imaging core at the First Core Labs, National Taiwan University College of Medicine, for technical support with cell sorting and image acquisition and analysis; and the Second Core Lab, Department of Medical Research, National Taiwan University Hospital, for technical support with RNA sequencing. This work was supported by the National Science and Technology Council of Taiwan (NSTC 112-2628-B-002-027, MOST 111-2628-B-002-035, MOST 110-2628-B-002-030, and MOST 109-2628-B-002-024 to CFC) and National Taiwan University (112 L7846 to CFC).

Author contributions
Che-Feng Chang: Conceptualization; supervision; funding acquisition; investigation; writing – original draft; writing – review and editing. **Tze-Yen Lin:** Conceptualization; data curation; formal analysis; validation; investigation; visualization; writing – original draft; writing – review and editing. **Danye Jiang:** Conceptualization; data curation; formal analysis; validation; investigation; visualization; writing – original draft; writing – review and editing. **Wan-Ru Chen:** Data curation; formal analysis; investigation. **Xin-Yu Zhang:** Data curation; formal analysis; investigation. **Jhih Syuan Lin:** Data curation; formal analysis; investigation. **Chih-Hung Chen:** Data curation; formal analysis; investigation. **Chia-Lang Hsu:** Resources; data curation; formal analysis; methodology. **Liang-Chuan Lai:** Resources; methodology. **Ping-Hung Chen:** Resources; methodology. **Kai-Chien Yang:** Resources; methodology. **Lauren H Sansing:** Writing – original draft; writing – review and editing.

Disclosure and competing interests statement
The authors declare that they have no conflict of interest.

References

Amara S, Whalen M, Tiriveedhi V (2016) High salt induces anti-inflammatory MPhi2-like phenotype in peripheral macrophages. *Biochem Biophys Rep* 7: 1 – 9

Askenase MH, Goods BA, Beatty HE, Steinschneider AF, Velazquez SE, Osherov A, Landreneau MJ, Carroll SL, Tran TB, Avram VS *et al* (2021) Longitudinal transcriptomics define the stages of myeloid activation in the living human brain after intracerebral hemorrhage. *Sci Immunol* 6: eabd6279

Babicki S, Arndt D, Marcu A, Liang Y, Grant JR, Maciejewski A, Wishart DS (2016) Heatmapper: web-enabled heat mapping for all. *Nucleic Acids Res* 44: W147 – W153

Babu R, Bagley JH, Di C, Friedman AH, Adamson C (2012) Thrombin and hemin as central factors in the mechanisms of intracerebral hemorrhage-induced secondary brain injury and as potential targets for intervention. *Neurosurg Focus* 32: E8

Bekkering S, Arts RJW, Novakovic B, Kourtzelis I, van der Heijden C, Li Y, Popa CD, Ter Horst R, van Tuijl J, Netea-Maier RT *et al* (2018) Metabolic induction of trained immunity through the mevalonate pathway. *Cell* 172: 135 – 146

Binger KJ, Gebhardt M, Heinig M, Rintisch C, Schroeder A, Neuhofer W, Hilgers K, Manzel A, Schwartz C, Kleinewietfeld M *et al* (2015) High salt reduces the activation of IL-4- and IL-13-stimulated macrophages. *J Clin Invest* 125: 4223 – 4238

van den Bossche J, Baardman J, Otto NA, van der Velden S, Neele AE, van den Berg SM, Luque-Martin R, Chen HJ, Boshuizen MC, Ahmed M *et al* (2016) Mitochondrial dysfunction prevents repolarization of inflammatory macrophages. *Cell Rep* 17: 684 – 696

Bosurgi L, Cao YG, Cabeza-Cabrerizo M, Tucci A, Hughes LD, Kong Y, Weinstein JS, Licona-Limon P, Schmid ET, Pelorosso F *et al* (2017) Macrophage function in tissue repair and remodeling requires IL-4 or IL-13 with apoptotic cells. *Science* 356: 1072 – 1076

Broderick JP, Brott TG, Duldner JE, Tomsick T, Huster G (1993) Volume of intracerebral hemorrhage. A powerful and easy-to-use predictor of 30-day mortality. *Stroke* 24: 987 – 993

Carlin LM, Stamatiades EG, Auffray C, Hanna RN, Glover L, Vizcay-Barrena G, Hedrick CC, Cook HT, Diebold S, Geissmann F (2013) Nr4a1-dependent Ly6C(low) monocytes monitor endothelial cells and orchestrate their disposal. *Cell* 153: 362 – 375

Chang CF, Wan J, Li Q, Renfroe SC, Heller NM, Wang J (2017) Alternative activation-skewed microglia/macrophages promote hematoma resolution in experimental intracerebral hemorrhage. *Neurobiol Dis* 103: 54 – 69

Chang CF, Goods BA, Askenase MH, Hammond MD, Renfroe SC, Steinschneider AF, Landreneau MJ, Ai Y, Beatty HE, da Costa LHA *et al* (2018) Erythrocyte efferocytosis modulates macrophages towards recovery after intracerebral hemorrhage. *J Clin Invest* 128: 607 – 624

Chang CF, Massey J, Osherov A, Angenendt da Costa LH, Sansing LH (2020) Bexarotene enhances macrophage erythrophagocytosis and hematoma clearance in experimental intracerebral hemorrhage. *Stroke* 51: 612 – 618

Chang CF, Goods BA, Askenase MH, Beatty HE, Osherov A, DeLong JH, Hammond MD, Massey J, Landreneau M, Love JC *et al* (2021) Divergent functions of tissue-resident and blood-derived macrophages in the hemorrhagic brain. *Stroke* 52: 1798 – 1808

Christ A, Gunther P, Lauterbach MAR, Duewell P, Biswas D, Pelka K, Scholz CJ, Oosting M, Haendler K, Bassler K *et al* (2018) Western diet triggers NLRP3-dependent innate immune reprogramming. *Cell* 172: 162 – 175

Clark W, Gunion-Rinker L, Lessov N, Hazel K (1998) Citicoline treatment for experimental intracerebral hemorrhage in mice. *Stroke* 29: 2136 – 2140

© 2023 The Authors

Corrocher FA, Bueno de Paiva L, Duarte ASS, Ferro KP, Silveira LDR, de Lima TI, Olalla Saad ST, Lazarini M (2018) Reduced expression of NR4A1 activates glycolytic pathway in acute promyelocytic leukemia cells. *Leuk Lymphoma* 59: 1501 – 1504

Cunningham KT, Finlay CM, Mills KHG (2021) Helminth imprinting of hematopoietic stem cells sustains anti-inflammatory trained innate immunity that attenuates autoimmune disease. *J Immunol* 206: 1618 – 1630

Deng S, Chen B, Huo J, Liu X (2022) Therapeutic potential of NR4A1 in cancer: focus on metabolism. *Front Oncol* 12: 972984

Divangahi M, Aaby P, Khader SA, Barreiro LB, Bekkering S, Chavakis T, van Crevel R, Curtis N, DiNardo AR, Dominguez-Andres J *et al* (2021) Trained immunity, tolerance, priming and differentiation: distinct immunological processes. *Nat Immunol* 22: 2 – 6

Dominguez-Andres J, Dos Santos JC, Bekkering S, Mulder WJM, van der Meer JW, Riksen NP, Joosten LAB, Netea MG (2022) Trained immunity: adaptation within innate immune mechanisms. *Physiol Rev* 103: 313 – 346

Edgar L, Akbar N, Braithwaite AT, Krausgruber T, Gallart-Ayala H, Bailey J, Corbin AL, Khoyratty TE, Chai JT, Alkhalil M *et al* (2021) Hyperglycemia induces trained immunity in macrophages and their precursors and promotes atherosclerosis. *Circulation* 144: 961 – 982

Fan A, Oladiran O, Shi XQ, Zhang J (2020) High-salt diet decreases mechanical thresholds in mice that is mediated by a CCR2-dependent mechanism. *J Neuroinflammation* 17: 179

Faraco G, Brea D, Garcia-Bonilla L, Wang G, Racchumi G, Chang H, Buendia I, Santisteban MM, Segarra SG, Koizumi K *et al* (2018) Dietary salt promotes neurovascular and cognitive dysfunction through a gut-initiated TH17 response. *Nat Neurosci* 21: 240 – 249

Faraco G, Hochrainer K, Segarra SG, Schaeffer S, Santisteban MM, Menon A, Jiang H, Holtzman DM, Anrather J, Iadecola C (2019) Dietary salt promotes cognitive impairment through tau phosphorylation. *Nature* 574: 686 – 690

Ferguson JF, Aden LA, Barbaro NR, Van Beusecum JP, Xiao L, Simmons AJ, Warden C, Pasic L, Himmel LE, Washington MK *et al* (2019) High dietary salt-induced dendritic cell activation underlies microbial dysbiosis-associated hypertension. *JCI Insight* 5: e126241

Freire PR, Conneely OM (2018) NR4A1 and NR4A3 restrict HSC proliferation via reciprocal regulation of C/EBPalpha and inflammatory signaling. *Blood* 131: 1081 – 1093

Gao P, You M, Li L, Zhang Q, Fang X, Wei X, Zhou Q, Zhang H, Wang M, Lu Z *et al* (2022) Salt-induced hepatic inflammatory memory contributes to cardiovascular damage through epigenetic modulation of SIRT3. *Circulation* 145: 375 – 391

GBDD Collaborators (2019) Health effects of dietary risks in 195 countries, 1990–2017: a systematic analysis for the Global Burden of Disease Study 2017. *Lancet* 393: 1958 – 1972

GBDS Collaborators (2021) Global, regional, and national burden of stroke and its risk factors, 1990–2019: a systematic analysis for the Global Burden of Disease Study 2019. *Lancet Neurol* 20: 795 – 820

Geisberger S, Bartolomaeus H, Neubert P, Willebrand R, Zasada C, Bartolomaeus T, McParland V, Swinnen D, Geuzens A, Maifeld A *et al* (2021) Salt transiently inhibits mitochondrial energetics in mononuclear phagocytes. *Circulation* 144: 144 – 158

Golan K, Vagima Y, Ludin A, Itkin T, Cohen-Gur S, Kalinkovich A, Kollet O, Kim C, Schajnovitz A, Ovadya Y *et al* (2012) S1P promotes murine progenitor cell egress and mobilization via S1P1-mediated ROS signaling and SDF-1 release. *Blood* 119: 2478 – 2488

Hanna RN, Carlin LM, Hubbeling HG, Nackiewicz D, Green AM, Punt JA, Geissmann F, Hedrick CC (2011) The transcription factor NR4A1 (Nur77) controls bone marrow differentiation and the survival of Ly6C- monocytes. *Nat Immunol* 12: 778 – 785

Hanna RN, Shaked I, Hubbeling HG, Punt JA, Wu R, Herrley E, Zaugg C, Pei H, Geissmann F, Ley K *et al* (2012) NR4A1 (Nur77) deletion polarizes macrophages toward an inflammatory phenotype and increases atherosclerosis. *Circ Res* 110: 416 – 427

Hartung F, Esser-von Bieren J (2022) Trained immunity in type 2 immune responses. *Mucosal Immunol* 15: 1447

He W, Xu J, Mu R, Li Q, Lv DL, Huang Z, Zhang J, Wang C, Dong L (2020) High-salt diet inhibits tumour growth in mice via regulating myeloid-derived suppressor cell differentiation. *Nat Commun* 11: 1732

He FJ, Campbell NRC, Woodward M, MacGregor GA (2021) Salt reduction to prevent hypertension: the reasons of the controversy. *Eur Heart J* 42: 2501 – 2505

Heras-Garvin A, Refolo V, Reindl M, Wenning GK, Stefanova N (2020) High-salt diet does not boost neuroinflammation and neurodegeneration in a model of alpha-synucleinopathy. *J Neuroinflammation* 17: 35

Hilgendorf I, Gerhardt LM, Tan TC, Winter C, Holderried TA, Chousterman BG, Iwamoto Y, Liao R, Zirlik A, Scherer-Crosbie M *et al* (2014) Ly-6Chigh monocytes depend on Nr4a1 to balance both inflammatory and reparative phases in the infarcted myocardium. *Circ Res* 114: 1611 – 1622

Ito M, Komai K, Mise-Omata S, Iizuka-Koga M, Noguchi Y, Kondo T, Sakai R, Matsuo K, Nakayama T, Yoshie O *et al* (2019) Brain regulatory T cells suppress astrogliosis and potentiate neurological recovery. *Nature* 565: 246 – 250

Jeljeli M, Riccio LGC, Doridot L, Chene C, Nicco C, Chouzenoux S, Deletang Q, Allanore Y, Kavian N, Batteux F (2019) Trained immunity modulates inflammation-induced fibrosis. *Nat Commun* 10: 5670

Jobin K, Stumpf NE, Schwab S, Eichler M, Neubert P, Rauh M, Adamowski M, Babyak O, Hinze D, Sivalingam S *et al* (2020) A high-salt diet compromises antibacterial neutrophil responses through hormonal perturbation. *Sci Transl Med* 12: eaay3850

Kaufmann E, Sanz J, Dunn JL, Khan N, Mendonca LE, Pacis A, Tzelepis F, Pernet E, Dumaine A, Grenier JC *et al* (2018) BCG educates hematopoietic stem cells to generate protective innate immunity against tuberculosis. *Cell* 172: 176 – 190

Kleinewietfeld M, Manzel A, Titze J, Kvakan H, Yosef N, Linker RA, Muller DN, Hafler DA (2013) Sodium chloride drives autoimmune disease by the induction of pathogenic TH17 cells. *Nature* 496: 518 – 522

Koenis DS, Medzikovic L, van Loenen PB, van Weeghel M, Huveneers S, Vos M, Evers-van Gogh IJ, van den Bossche J, Speijer D, Kim Y *et al* (2018) Nuclear receptor Nur77 limits the macrophage inflammatory response through transcriptional reprogramming of mitochondrial metabolism. *Cell Rep* 24: 2127 – 2140

Land RH, Rayne AK, Vanderbeck AN, Barlowe TS, Manjunath S, Gross M, Eiger S, Klein PS, Cunningham NR, Huang J *et al* (2015) The orphan nuclear receptor NR4A1 specifies a distinct subpopulation of quiescent myeloid-biased long-term HSCs. *Stem Cells* 33: 278 – 288

Liao Y, Smyth GK, Shi W (2014) featureCounts: an efficient general purpose program for assigning sequence reads to genomic features. *Bioinformatics* 30: 923 – 930

Liu Y, Chen Y, Deng X, Zhou J (2020) ATF3 prevents stress-induced hematopoietic stem cell exhaustion. *Front Cell Dev Biol* 8: 585771

Marinkovic G, Koenis DS, de Camp L, Jablonowski R, Graber N, de Waard V, de Vries CJ, Goncalves I, Nilsson J, Jovinge S *et al* (2020) S100A9 links inflammation and repair in myocardial infarction. *Circ Res* 127: 664 – 676

Massberg S, Schaerli P, Knezevic-Maramica I, Kollnberger M, Tubo N, Moseman EA, Huff IV, Junt T, Wagers AJ, Mazo IB *et al* (2007)

Immunosurveillance by hematopoietic progenitor cells trafficking through blood, lymph, and peripheral tissues. *Cell* 131: 994 – 1008

Mitroulis I, Ruppova K, Wang B, Chen LS, Grzybek M, Grinenko T, Eugster A, Troullinaki M, Palladini A, Kourtzelis I *et al* (2018) Modulation of myelopoiesis progenitors is an integral component of trained immunity. *Cell* 172: 147 – 161

Muller DN, Wilck N, Haase S, Kleinewietfeld M, Linker RA (2019) Sodium in the microenvironment regulates immune responses and tissue homeostasis. *Nat Rev Immunol* 19: 243 – 254

Netea MG, Dominguez-Andres J, Barreiro LB, Chavakis T, Divangahi M, Fuchs E, Joosten LAB, van der Meer JWM, Mhlanga MM, Mulder WJM *et al* (2020) Defining trained immunity and its role in health and disease. *Nat Rev Immunol* 20: 375 – 388

Oguchi H, Sasamura H, Shinoda K, Morita S, Kono H, Nakagawa K, Ishiguro K, Hayashi K, Nakamura M, Azegami T *et al* (2014) Renal arteriolar injury by salt intake contributes to salt memory for the development of hypertension. *Hypertension* 64: 784 – 791

Robinson MD, McCarthy DJ, Smyth GK (2010) edgeR: a Bioconductor package for differential expression analysis of digital gene expression data. *Bioinformatics* 26: 139 – 140

Ruberti S, Bianchi E, Guglielmelli P, Rontauroli S, Barbieri G, Tavernari L, Fanelli T, Norfo R, Pennucci V, Fattori GC *et al* (2018) Involvement of MAF/SPP1 axis in the development of bone marrow fibrosis in PMF patients. *Leukemia* 32: 438 – 449

Rucker AJ, Rudemiller NP, Crowley SD (2018) Salt, hypertension, and immunity. *Annu Rev Physiol* 80: 283 – 307

Safe S, Shrestha R, Mohankumar K (2021) Orphan nuclear receptor 4A1 (NR4A1) and novel ligands. *Essays Biochem* 65: 877 – 886

Schaar KL, Brenneman MM, Savitz SI (2010) Functional assessments in the rodent stroke model. *Exp Transl Stroke Med* 2: 13

Sekiya T, Kashiwagi I, Yoshida R, Fukaya T, Morita R, Kimura A, Ichinose H, Metzger D, Chambon P, Yoshimura A (2013) Nr4a receptors are essential for thymic regulatory T cell development and immune homeostasis. *Nat Immunol* 14: 230 – 237

Shi L, Sun Z, Su W, Xu F, Xie D, Zhang Q, Dai X, Iyer K, Hitchens TK, Foley LM *et al* (2021) Treg cell-derived osteopontin promotes microglia-mediated white matter repair after ischemic stroke. *Immunity* 54: 1527 – 1542

Tsai HH, Hsieh YC, Lin JS, Kuo ZT, Ho CY, Chen CH, Chang CF (2022) Functional investigation of meningeal lymphatic system in experimental intracerebral hemorrhage. *Stroke* 53: 987 – 998

Wendeln AC, Degenhardt K, Kaurani L, Gertig M, Ulas T, Jain G, Wagner J, Hasler LM, Wild K, Skodras A *et al* (2018) Innate immune memory in the brain shapes neurological disease hallmarks. *Nature* 556: 332 – 338

Wenstedt EF, Verberk SG, Kroon J, Neele AE, Baardman J, Claessen N, Pasaoglu OT, Rademaker E, Schrooten EM, Wouda RD *et al* (2019) Salt increases monocyte CCR2 expression and inflammatory responses in humans. *JCI Insight* 4: e130508

Wilck N, Balogh A, Marko L, Bartolomaeus H, Muller DN (2019) The role of sodium in modulating immune cell function. *Nat Rev Nephrol* 15: 546 – 558

Wu C, Yosef N, Thalhamer T, Zhu C, Xiao S, Kishi Y, Regev A, Kuchroo VK (2013) Induction of pathogenic TH17 cells by inducible salt-sensing kinase SGK1. *Nature* 496: 513 – 517

Wu JS, Tsai HD, Cheung WM, Hsu CY, Lin TN (2016) PPAR-gamma ameliorates neuronal apoptosis and ischemic brain injury via suppressing NF-kappaB-driven p22phox transcription. *Mol Neurobiol* 53: 3626 – 3645

Xu J, Chen Z, Yu F, Liu H, Ma C, Xie D, Hu X, Leak RK, Chou SHY, Stetler RA *et al* (2020) IL-4/STAT6 signaling facilitates innate hematoma resolution and neurological recovery after hemorrhagic stroke in mice. *Proc Natl Acad Sci U S A* 117: 32679 – 32690

Yao Y, Jeyanathan M, Haddadi S, Barra NG, Vaseghi-Shanjani M, Damjanovic D, Lai R, Afkhami S, Chen Y, Dvorkin-Gheva A *et al* (2018) Induction of autonomous memory alveolar macrophages requires T cell help and is critical to trained immunity. *Cell* 175: 1634 – 1650

Zelcer N, Tontonoz P (2006) Liver X receptors as integrators of metabolic and inflammatory signaling. *J Clin Invest* 116: 607 – 614

Zhang WC, Zheng XJ, Du LJ, Sun JY, Shen ZX, Shi C, Sun S, Zhang Z, Chen XQ, Qin M *et al* (2015) High salt primes a specific activation state of macrophages, M(Na). *Cell Res* 25: 893 – 910

Zhang T, Wang D, Li X, Jiang Y, Wang C, Zhang Y, Kong Q, Tian C, Dai Y, Zhao W *et al* (2020) Excess salt intake promotes M1 microglia polarization via a p38/MAPK/AR-dependent pathway after cerebral ischemia in mice. *Int Immunopharmacol* 81: 106176

Zhang H, Chen T, Ren J, Xia Y, Onuma A, Wang Y, He J, Wu J, Wang H, Hamad A *et al* (2021) Pre-operative exercise therapy triggers anti-inflammatory trained immunity of Kupffer cells through metabolic reprogramming. *Nat Metab* 3: 843 – 858

Zhang M, Liu ZZ, Aoshima K, Cai WL, Sun H, Xu T, Zhang Y, An Y, Chen JF, Chan LH *et al* (2022) CECR2 drives breast cancer metastasis by promoting NF-kappaB signaling and macrophage-mediated immune suppression. *Sci Transl Med* 14: eabf5473

Zhao X, Sun G, Zhang J, Strong R, Song W, Gonzales N, Grotta JC, Aronowski J (2007) Hematoma resolution as a target for intracerebral hemorrhage treatment: role for peroxisome proliferator-activated receptor gamma in microglia/macrophages. *Ann Neurol* 61: 352 – 362

Zhao X, Ting SM, Liu CH, Sun G, Kruzel M, Roy-O'Reilly M, Aronowski J (2017) Neutrophil polarization by IL-27 as a therapeutic target for intracerebral hemorrhage. *Nat Commun* 8: 602

License: This is an open access article under the terms of the Creative Commons Attribution License, which permits use, distribution and reproduction in any medium, provided the original work is properly cited.

 © 2023 The Authors

Expanded View Figures

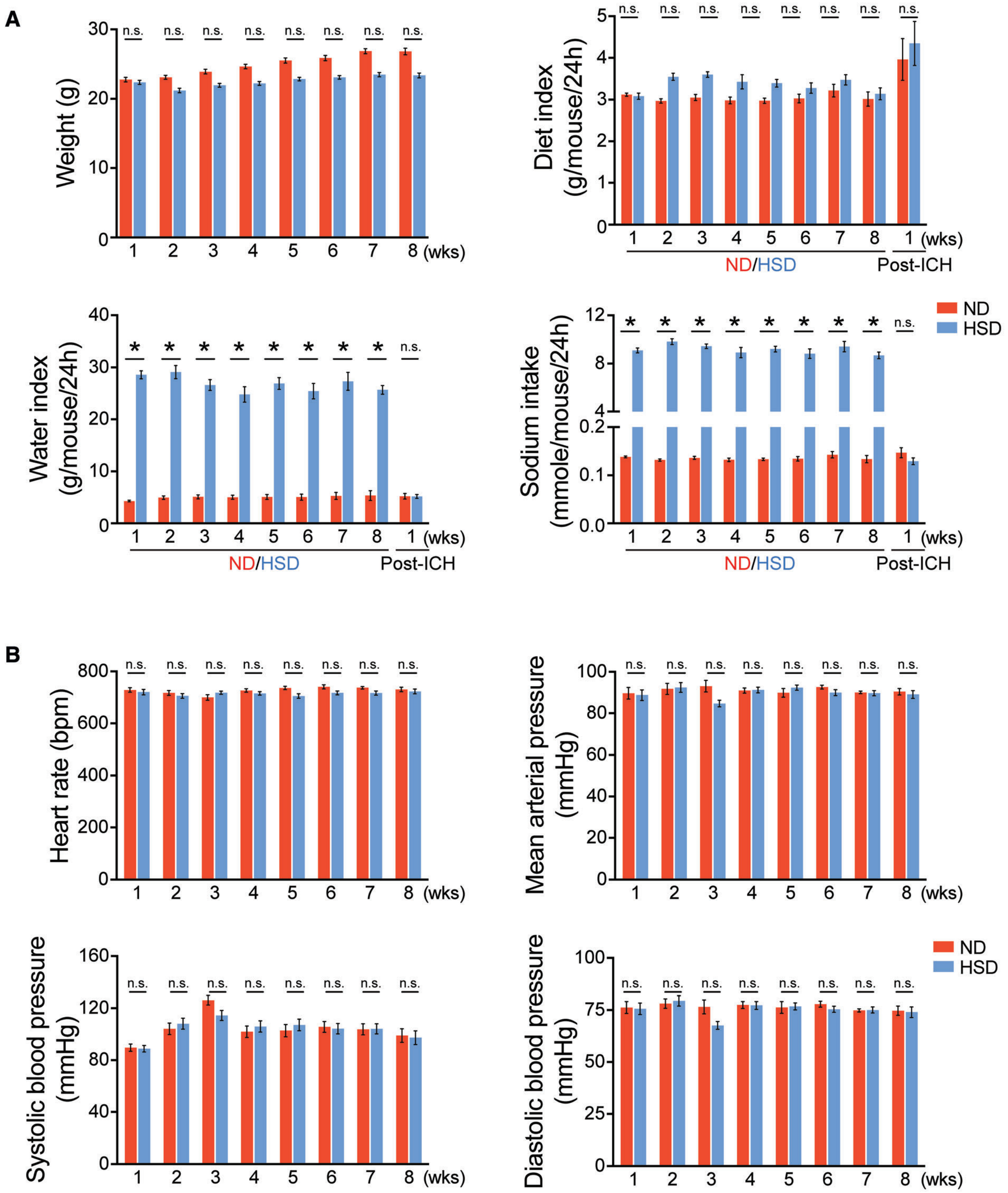

Figure EV1.

Figure EV1. Physiological measurements of ND and HSD mice.

A Graphs depicting body weight, food intake, water intake, and sodium intake in ND (n = 22–33) and HSD (n = 24–35) mice over 8 weeks of diet manipulation. Two-way ANOVA and Bonferroni test. n: biological replicates.

B Graphs depicting heart rate, mean arterial pressure, systolic blood pressure, and diastolic blood pressure in ND and HSD animals over 8 weeks of diet manipulation. n = 7–11/group, two-way ANOVA and Bonferroni test. n: biological replicates.

Data information: All data are mean ± SEM; $^*P < 0.05$, n.s., not significant.
Source data are available online for this figure.

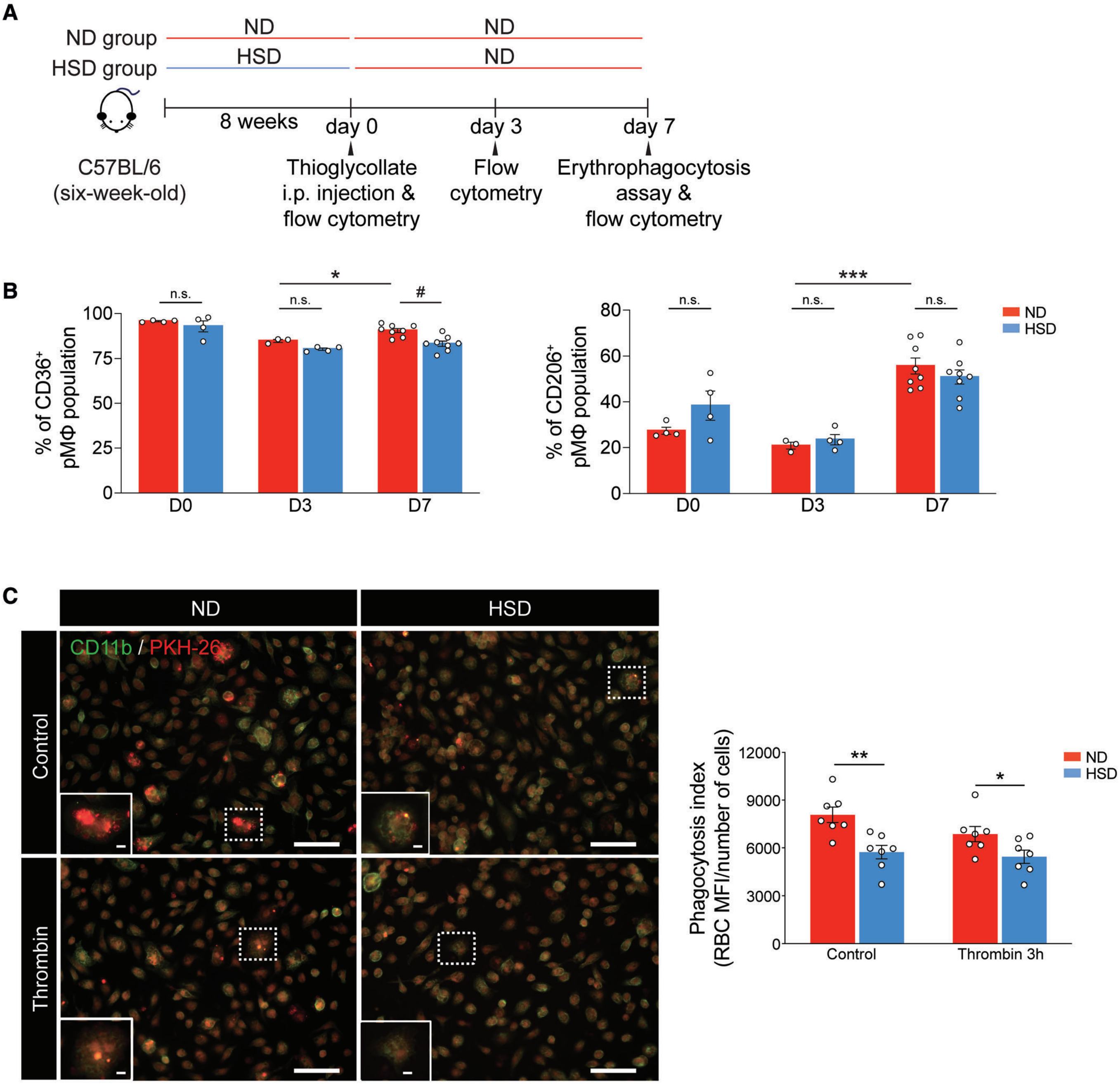

Figure EV2.

 © 2023 The Authors

Figure EV2. HSD reduces alternative activation of peritoneal macrophages in a thioglycollate-elicited peritoneal model.

A Schematic of experimental design.
B Bar graphs depicting the percentages of $CD36^+$ and $CD206^+$ peritoneal macrophages from ND and HSD mice on days 0, 3, and 7 following thioglycollate injection. n = 4/group on day 0, n = 3 ND, 4 HSD on day 3, and n = 8/group on day 7, two-way ANOVA and Bonferroni test. n: biological replicates.
C Representative images showing engulfment of heat-shocked PKH26-labeled erythrocytes (red) in $CD11b^+$ (green) ND and HSD peritoneal macrophages with or without thrombin stimulation. Quantifications of erythrophagocytosis in ND and HSD peritoneal macrophages with or without thrombin stimulation. n = 7/group, Student's t-test. n: biological replicates. Scale bar: 50 μm and 5 μm high-magnification insets.

Data information: All data are mean ± SEM, each symbol represents one mouse or one biological replicate, $^*P < 0.05$, $^{**}P < 0.01$, $^{***}P < 0.001$, and $^{\#}P < 0.001$, n.s., not significant.
Source data are available online for this figure.

Figure EV3. HSD impairs mitochondrial oxidative phosphorylation and promotes glycolysis in bone marrow (BM) cells.

A OCR traces and maximum respiratory capacity of BM cells after 4-week ND and HSD. n = 9 ND, 6 HSD, Student's t-test. n: biological replicates.
B OCR traces and maximum respiratory capacity of BM cells after 8-week ND and HSD. n = 9 ND, 6 HSD, Student's t-test. n: biological replicates.
C ECAR traces and glycolytic capacity of BM cells after 4-week ND and HSD. n = 5/group, Student's t-test. n: biological replicates.
D ECAR traces and glycolytic capacity of BM cells after 8-week ND and HSD. n = 8/group, Student's t-test. n: biological replicates.

Data information: All data are mean ± SEM, each symbol represents one mouse, $^*P < 0.05$, $^{**}P < 0.01$, and $^{***}P < 0.001$; OCR, oxygen consumption rate, OM, oligomycin, FCCP, carbonyl cyanide-4 (trifluoromethoxy) phenylhydrazone, ROT/AA, rotenone and antimycin A, ECAR, extracellular acidification rate, and 2-DG, 2-deoxy-D-glucose.
Source data are available online for this figure.

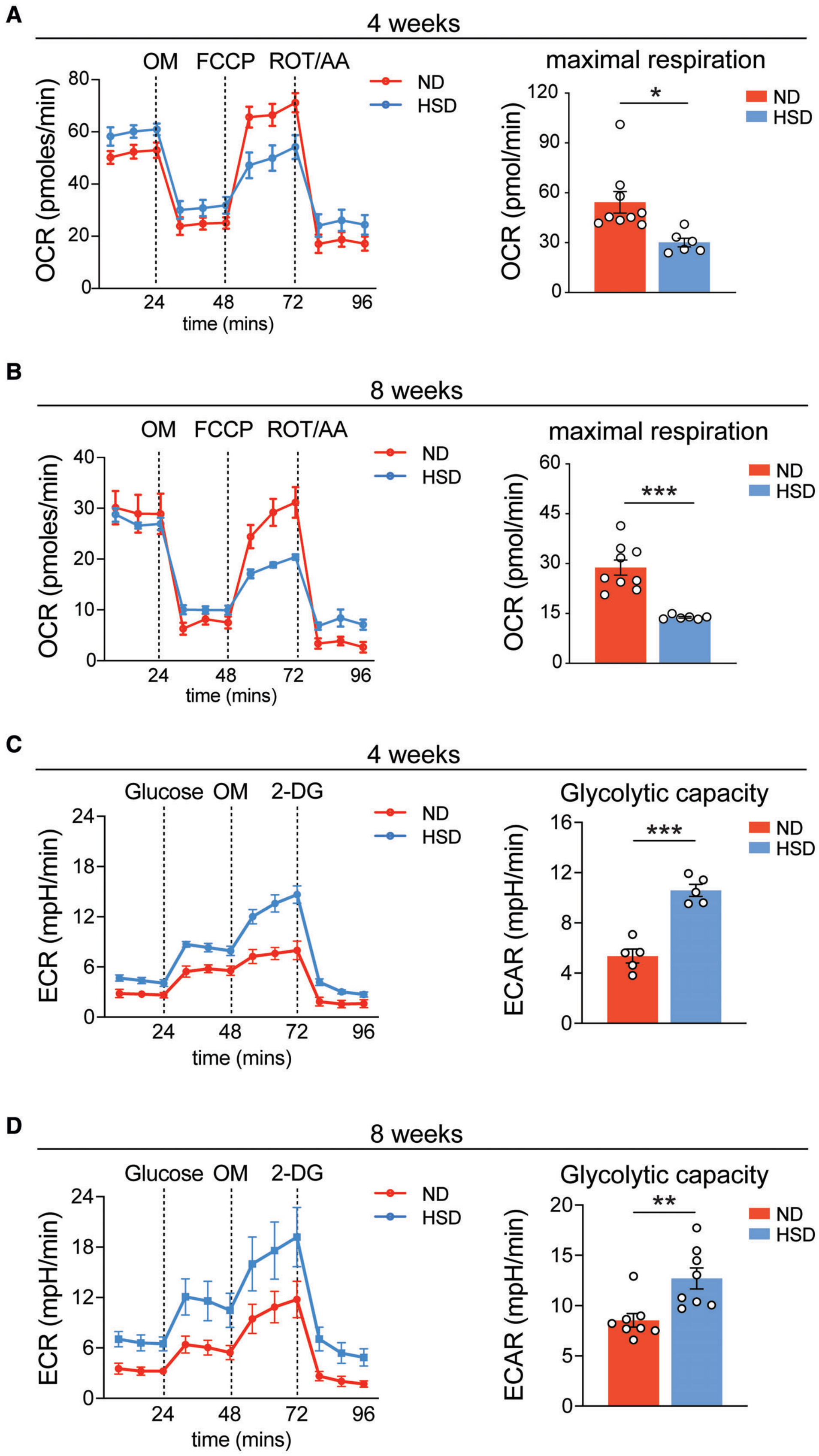

Figure EV3.

© 2023 The Authors

Figure EV4. Four-week HSD reduces ICH recovery, HSD➔ND chimeric mice have worse functional outcomes after collagenase ICH, and ND BM cell transplantation to HSD mice rescues HSD-aggravated ICH injuries.

A Schematic of the 4-week diet regimen. Six-week-old mice were fed with ND or HSD for 4 weeks before being subjected to ICH surgery. Behavioral tests and flow cytometry were performed 1 week after blood ICH induction.
B Cylinder test results of mice after 4-week ND and HSD on days 1, 3, and 7 after ICH. n = 9 ND, 11 HSD, two-way ANOVA and Bonferroni test. n: biological replicates.
C Percentages of $CD36^+$, $CD206^+$, and $CD36^+CD206^+$ MDMs in 4-week ND and HSD mice after ICH. n = 5/group, Student's t-test. n: biological replicates.
D Percentages of $CD36^+$ or $CD206^+$ microglia (MG) after 4-week ND and HSD. n = 5/group, Student's t-test. n: biological replicates.
E Survival rates of ND and HSD chimeras that were subjected to a collagenase ICH model.
F Cylinder test and forelimb placement test results of ND➔ND and HSD➔ND chimeras. n = 6 ND chimeras and 3 HSD chimeras on day 3, n = 4 ND chimeras and 2 HSD chimeras on day 10.
G Schematic of experimental design. ND and HSD BM were transplanted into HSD recipients.
H Cylinder test of ND➔HSD (n = 7) and HSD➔HSD (n = 8) chimeric mice on days 3, 7, and 10 post-ICH. Two-way ANOVA and Bonferroni test. n: biological replicates.
I Representative images and quantification of $Ly76^+$ hematomas co-labeled with DAPI in ND➔HSD (n = 7) and HSD➔HSD (n = 8) chimeras. Student's t-test. n: biological replicates. Scale bar: 500 μm.
J Representative images and quantifications of $NeuN^+$ neurons and $GFAP^+$ cells co-labeled with DAPI in ND➔HSD (n = 7) and HSD➔HSD (n = 8) chimeras. Student's t-test. n: biological replicates. Scale bar: 100 μm.
K Representative images and quantifications of $CD206^+IBA1^+$ and $MAC2^+IBA1^+$ cells in ND➔HSD (n = 8) and HSD➔HSD (n = 8) chimeras. Student's t-test. n: biological replicates. Scale bar: 100 μm.

Data information: All data are mean ± SEM, each symbol represents one mouse or one biological replicate, *$P < 0.05$, **$P < 0.01$, and ***$P < 0.001$, n.s., not significant. Source data are available online for this figure.

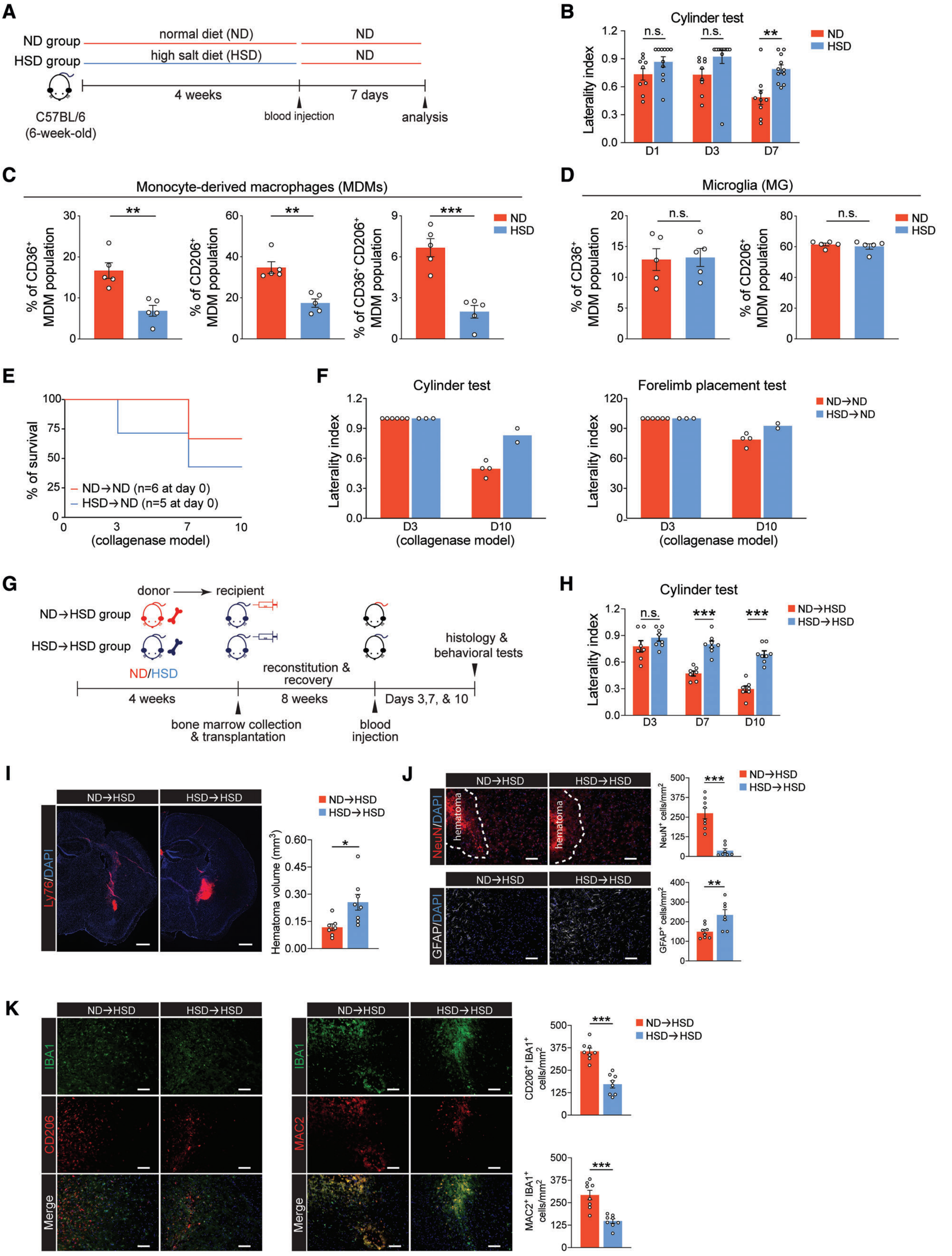

Figure EV4.

 © 2023 The Authors

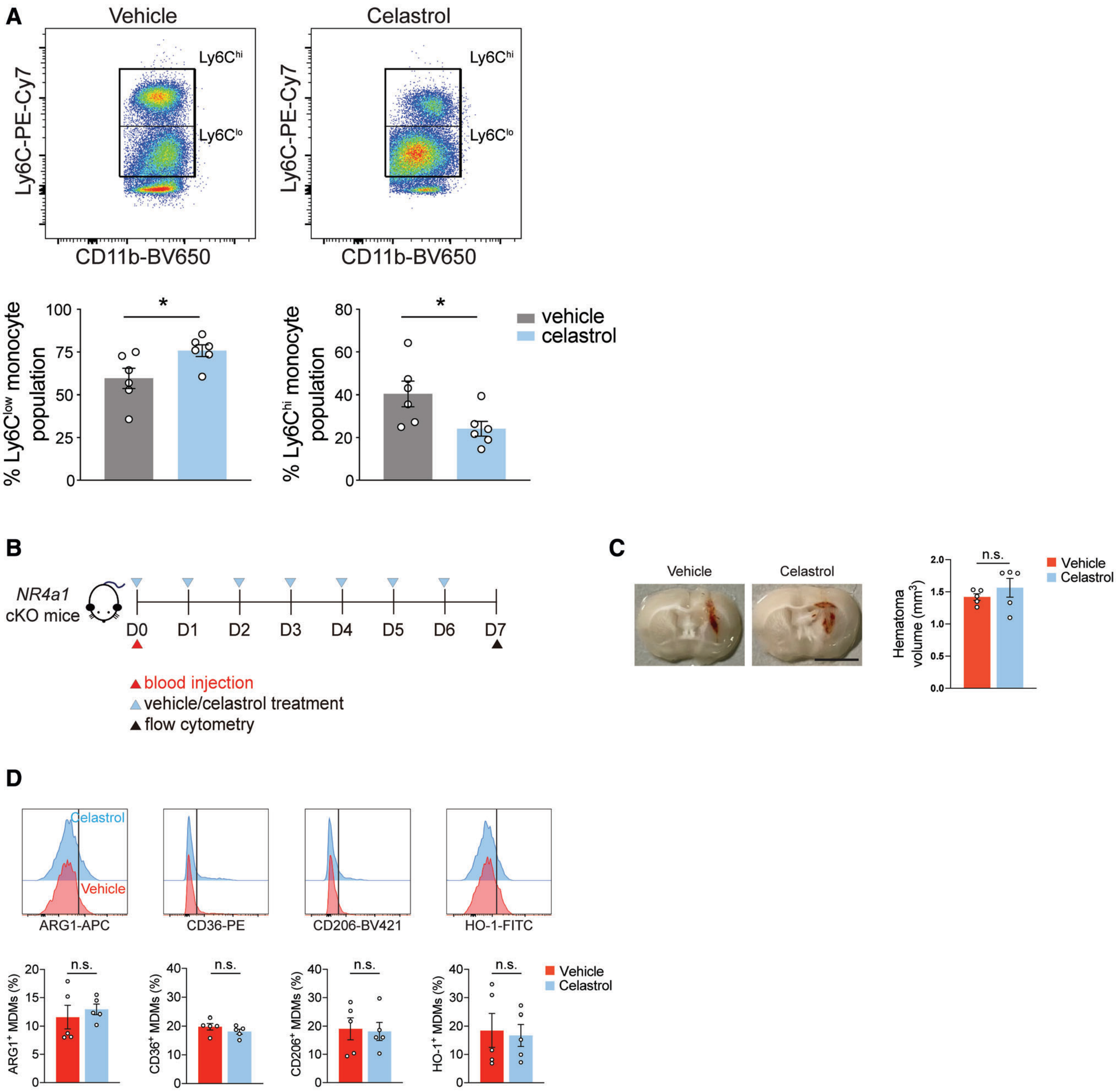

Figure EV5. Celastrol treatment increases Ly6C^{low} monocytes and decreases Ly6C^{hi} proinflammatory monocytes, and deletion of NR4a family in macrophages abolishes the protective effects of celastrol in the ICH brain.

A Top: representative pseudocolor plots showing Ly6C^{hi} and Ly6C^{low} monocytes in HSD mice that received vehicle or celastrol treatments. Bottom: quantifications showing percentages of Ly6C^{low} and Ly6C^{hi} monocytes in vehicle and celastrol groups. n = 6/group, Student's t-test. n: biological replicates.

B Schematic of celastrol treatment in *NR4a1* cKO mice and experimental design.

C Representative images and quantification of brain hematoma in vehicle- and celastrol-treated *NR4a1* cKO mice on day 7 post-ICH. n = 5/group, Student's t-test. n: biological replicates. Scale bar: 5 mm.

D Representative histograms and quantifications of ARG1$^+$, CD36$^+$, CD206$^+$, and HO-1$^+$ MDMs in vehicle- and celastrol-treated *NR4a1* cKO ICH mice. n = 5/group, Student's t-test. n: biological replicates.

Data information: All data are mean ± SEM, each symbol represents one mouse, *$P < 0.05$, n.s., not significant.
Source data are available online for this figure.

Trained immunity induced by high salt diet impedes stroke recovery

Tze-Yen Lin, Danye Jiang, Wan-Ru Chen, Jhih Syuan Lin, Xin-Yu Zhang, Chih-Hung Chen, Chia-Lang Hsu, Liang-Chuan Lai, Ping-Hung Chen, Kai-Chien Yang, Lauren H Sansing, Che-Feng Chang*

*Correspondence to:

Che-Feng Chang, Department and Graduate Institute of Physiology, National Taiwan University College of Medicine, No. 1, Sec. 1, Jen-Ai Rd., Room 1038, Taipei 100, Taiwan.

Phone: +886-2-23123456 ext. 288238; E-mail: chefengchang@ntu.edu.tw

Contents

Appendix Figure S1. Gating strategies to distinguish monocyte-derived macrophages (MDMs) and microglia in the ICH brain……………………………………………………………………2

Appendix Figure S2. Significantly enriched canonical pathways identified in HSD HSPCs…...3

Appendix Figure S3. The efficiency of NR4a1-expressing lentivirus and NR4a1 overexpression ………………………………………………………………………………….………....5

Appendix Figure S1

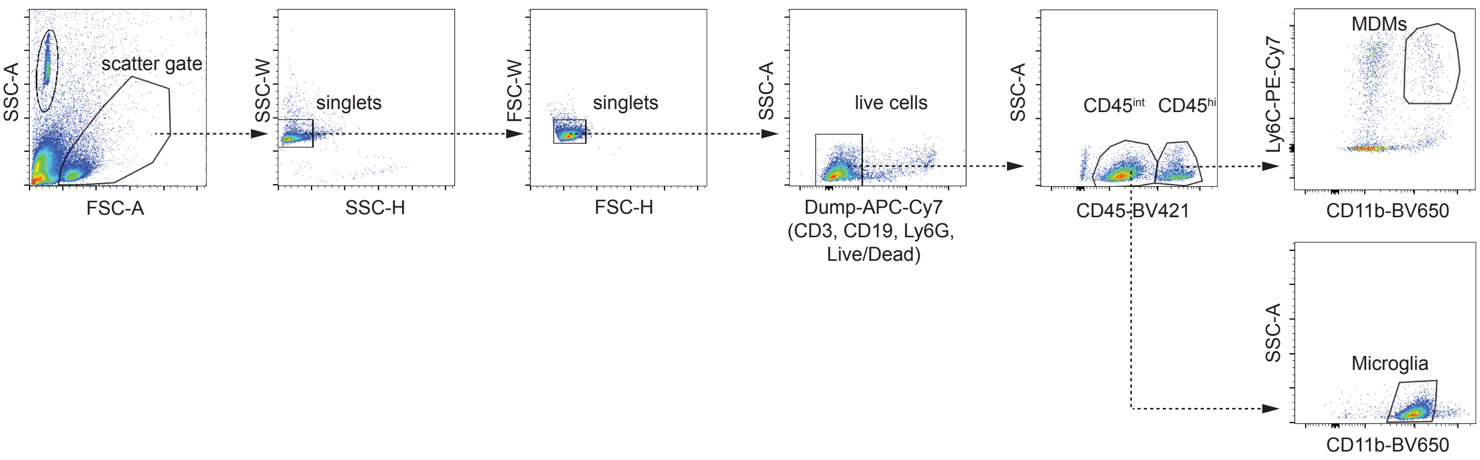

Appendix Figure S1. Gating strategies to distinguish monocyte-derived macrophages (MDMs) and microglia in the ICH brain. MDMs are identified as the $CD45^{hi}CD11b^{+}Ly6C^{+}$ population and microglia as the $CD45^{int}CD11b^{+}$ population.

Appendix Figure S2

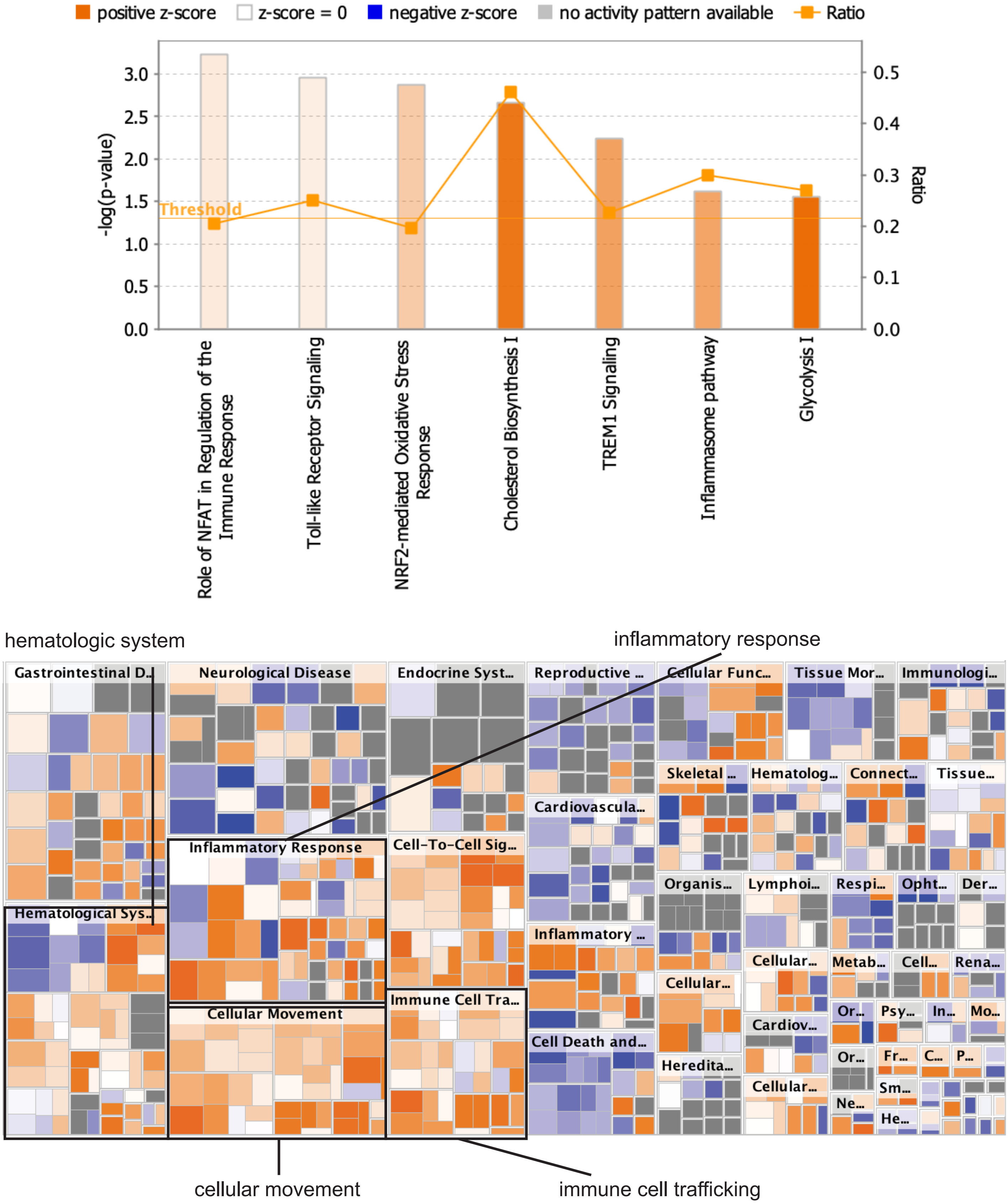

Appendix Figure S2. Ingenuity pathway analysis (IPA) of significantly enriched canonical pathways identified in HSD HSPCs. Top: The classical pathway enrichment analysis indicates pathway activation (orange, z-score >0). Bottom: Heat maps of disease and functional categories show the relationships between changes in differentially expressed gene levels and the activation and inhibition of diseases and functions. Orange indicates that the disease or functional status is activated (z-score >0), blue indicates that the disease or functional status is suppressed (z-score <0), and gray indicates that the disease or functional status is undetermined (z-score cannot be calculated).

Appendix Figure S3

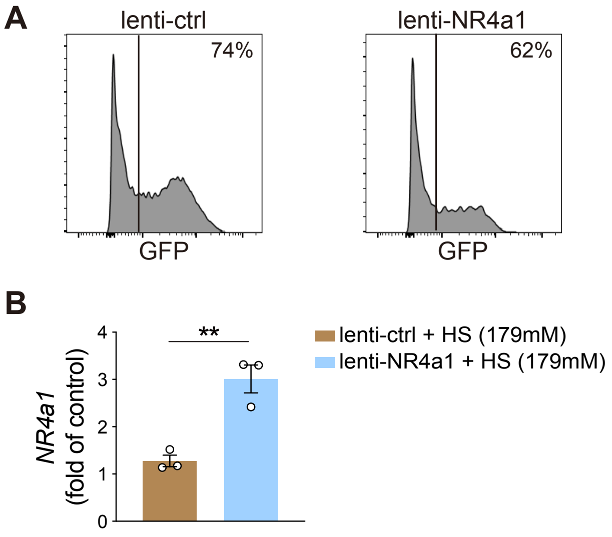

Appendix Figure S3. The efficiency of NR4a1-expressing lentivirus and NR4a1 overexpression. (**A**) Representative histograms showing transduction efficiency of GFP-tagged control lentiviral vector (lenti-ctrl) and NR4a1-expressing vector (lenti-NR4a1). The transduction efficiency in bone marrow cells was assessed by measuring the percentage of GFP+ cells. (**B**) Expression of NR4a1 in BMDMs after NR4a1-expressing lentivirus transduction and high-salt differentiation (lenti-NR4a1+HS) compared to those with control vector transduction and high-salt differentiation (lenti-ctrl+HS). Data are mean ± SEM, *n*=3/group, ***P* < 0.01 (Student's *t*-test), *n*: biological replicates.
