# Supplementary material for: Trained immunity induced by high‐salt diet impedes stroke recovery
Source: EMBO Rep. 2023 Nov 15;24(12):e57164. doi: 10.15252/embr.202357164 (PMC10702837; doi:10.15252/embr.202357164)
Supplement: Supplementary file 14 [file EMBR-24-e57164-s010.pdf]

EMBO reports

Trained immunity induced by high salt diet impedes stroke recovery

Tze-Yen Lin, Danye Jiang, Wan-Ru Chen, Jhih Syuan Lin, Xin-Yu Zhang, Chih-Hung Chen, Chia-Lang Hsu, Liang-Chuan Lai, Ping-Hung Chen, Kai-Chien Yang, Lauren H Sansing, Che-Feng Chang
DOI: 10.15252/embr.202357164

Corresponding author(s): Che-Feng Chang (chefengchang@ntu.edu.tw)

Review Timeline:

Submission Date:	10th Mar 23
Editorial Decision:	19th Apr 23
Revision Received:	23rd Aug 23
Editorial Decision:	17th Oct 23
Revision Received:	22nd Oct 23
Accepted:	26th Oct 23

Editor: Achim Breiling

Transaction Report:

(Note: With the exception of the correction of typographical or spelling errors that could be a source of ambiguity, letters and reports are not edited. Depending on transfer agreements, referee reports obtained elsewhere may or may not be included in this compilation. Referee reports are anonymous unless the Referee chooses to sign their reports.)

1st Editorial Decision **19th Apr 2023**

Dear Dr. Chang,

Thank you for the transfer of your manuscript to EMBO reports. I have now received the reports from the three referees that were asked to evaluate your study, which can be found at the end of this message.

As you will see, the referees state that these findings are of interest. However, they have several comments, concerns, and suggestions, indicating that a major revision of the manuscript is necessary to allow publication of the study in EMBO reports. As the reports are below, and all the referee concerns need to be addressed, I will not detail them here.

Given the constructive referee comments, I would like to invite you to revise your manuscript with the understanding that all referee concerns must be addressed in the revised manuscript and in a detailed point-by-point response. Acceptance of your manuscript will depend on a positive outcome of a second round of review. It is EMBO reports policy to allow a single round of revision only and acceptance of the manuscript will therefore depend on the completeness of your responses included in the next, final version of the manuscript.

Revised manuscripts should be submitted within three months of a request for revision. Please contact me to discuss the revision (also by video chat) if you have questions or comments regarding the revision, or should you need additional time.

When submitting your revised manuscript, please also carefully review the instructions that follow below.

PLEASE NOTE THAT upon resubmission revised manuscripts are subjected to an initial quality control prior to exposition to re-review. Upon failure in the initial quality control, the manuscripts are sent back to the authors, which may lead to delays. Frequent reasons for such a failure are the lack of the data availability section (please see below) and the presence of statistics based on n=2 (the authors are then asked to present scatter plots or provide more data points).

When submitting your revised manuscript, we will require:

1) a .docx formatted version of the final manuscript text (including legends for main figures, EV figures and tables), but without the figures included. Figure legends should be compiled at the end of the manuscript text.

2) individual production quality figure files as .eps, .tif, .jpg (one file per figure), of main figures (up to 8) and EV figures. Please upload these as separate, individual files upon re-submission.

The Expanded View format, which will be displayed in the main HTML of the paper in a collapsible format, has replaced the Supplementary information. You can submit up to 5 images as Expanded View. Please follow the nomenclature Figure EV1, Figure EV2 etc. The figure legend for these should be included in the main manuscript document file in a section called Expanded View Figure Legends after the main Figure Legends section. Additional Supplementary material should be supplied as a single pdf file labeled Appendix. The Appendix should have page numbers and needs to include a table of content on the first page (with page numbers) and legends for all content. Please follow the nomenclature Appendix Figure Sx, Appendix Table Sx etc. throughout the text, and also label the figures and tables according to this nomenclature.

For more details, please refer to our guide to authors:
http://www.embopress.org/page/journal/14693178/authorguide#manuscriptpreparation

Please consult our guide for figure preparation:
http://wol-prod-cdn.literatumonline.com/pb-assets/embo-site/EMBOPress_Figure_Guidelines_061115-1561436025777.pdf

See also the guidelines for figure legend preparation:
https://www.embopress.org/page/journal/14693178/authorguide#figureformat

3) a .docx formatted letter INCLUDING the reviewers' reports and your detailed point-by-point responses to their comments. As part of the EMBO Press transparent editorial process, the point-by-point response is part of the Review Process File (RPF), which will be published alongside your paper.

4) a complete author checklist, which you can download from our author guidelines (https://www.embopress.org/page/journal/14693178/authorguide). Please insert page numbers in the checklist to indicate where the requested information can be found in the manuscript. The completed author checklist will also be part of the RPF.

Please also follow our guidelines for the use of living organisms, and the respective reporting guidelines:
http://www.embopress.org/page/journal/14693178/authorguide#livingorganisms

5) that primary datasets produced in this study (e.g. RNA-seq, ChIP-seq, structural and array data) are deposited in an

appropriate public database. If no primary datasets have been deposited, please also state this in a dedicated section (e.g. 'No primary datasets have been generated and deposited'), see below.

See also: http://embor.embopress.org/authorguide#datadeposition

Please remember to provide a reviewer password if the datasets are not yet public.

The accession numbers and database should be listed in a formal "Data Availability" section (placed after Materials & Methods) that follows the model below. This is now mandatory (like the COI statement). Please note that the Data Availability Section is restricted to new primary data that are part of this study. This section is mandatory. As indicated above, if no primary datasets have been deposited, please state this in this section

Data availability

The datasets produced in this study are available in the following databases:

- RNA-Seq data: Gene Expression Omnibus GSE46843 (https://www.ncbi.nlm.nih.gov/geo/query/acc.cgi?acc=GSE46843)
- [data type]: [name of the resource] [accession number/identifier/doi] ([URL or identifiers.org/DATABASE:ACCESSION])

*** Note - All links should resolve to a page where the data can be accessed. ***

Moreover, I have these editorial requests:

6) We now request the publication of original source data with the aim of making primary data more accessible and transparent to the reader. Our source data coordinator will contact you to discuss which figure panels we would need source data for and will also provide you with helpful tips on how to upload and organize the files.

7) Our journal encourages inclusion of *data citations in the reference list* to directly cite datasets that were re-used and obtained from public databases. Data citations in the article text are distinct from normal bibliographical citations and should directly link to the database records from which the data can be accessed. In the main text, data citations are formatted as follows: "Data ref: Smith et al, 2001" or "Data ref: NCBI Sequence Read Archive PRJNA342805, 2017". In the Reference list, data citations must be labeled with "[DATASET]". A data reference must provide the database name, accession number/identifiers and a resolvable link to the landing page from which the data can be accessed at the end of the reference. Further instructions are available at: http://www.embopress.org/page/journal/14693178/authorguide#referencesformat

8) Regarding data quantification and statistics, please make sure that the number "n" for how many independent experiments were performed, their nature (biological versus technical replicates), the bars and error bars (e.g. SEM, SD) and the test used to calculate p-values is indicated in the respective figure legends (also for potential EV figures and all those in the final Appendix). Please also check that all the p-values are explained in the legend, and that these fit to those shown in the figure. Please provide statistical testing where applicable. Please avoid the phrase 'independent experiment', but clearly state if these were biological or technical replicates. Please also indicate (e.g. with n.s.) if testing was performed, but the differences are not significant. In case n=2, please show the data as separate datapoints without error bars and statistics. See also: http://www.embopress.org/page/journal/14693178/authorguide#statisticalanalysis

If n<5, please show single datapoints for diagrams.

9) Please add scale bars of similar style and thickness to microscopic images, using clearly visible black or white bars (depending on the background). Please place these in the lower right corner of the images themselves. Please do not write on or near the bars in the image but define the size in the respective figure legend.

10) Please also note our reference format:
http://www.embopress.org/page/journal/14693178/authorguide#referencesformat

11) We updated our journal's competing interests policy in January 2022 and request authors to consider both actual and perceived competing interests. Please review the policy https://www.embopress.org/competing-interests and update your competing interests if necessary. Please name this section 'Disclosure and Competing Interests Statement' and put it after the Acknowledgements section.

12) We now use CRediT to specify the contributions of each author in the journal submission system. CRediT replaces the author contribution section. Please use the free text box to provide more detailed descriptions and do not provide your final manuscript text file with an author contributions section. See also guide to authors:
https://www.embopress.org/page/journal/14693178/authorguide#authorshipguidelines

Finally, please add up to 5 keywords to the manuscript text below the abstract and order the manuscript sections like this, using

these names:
Title page - Abstract - Keywords - Introduction - Results - Discussion - Materials and Methods - Data availability section - Acknowledgements - Disclosure and Competing Interests Statement - References - Figure legends - Expanded View Figure legends

I look forward to seeing a revised version of your manuscript when it is ready. Please let me know if you have questions or comments regarding the revision.

Yours sincerely,

Achim Breiling
Senior Editor
EMBO Reports

Referee #1:

This is an interesting study that investigates the original hypothesis that a high-slat diet (HSD) induces trained immunity through long-term changes in hematopoietic stem cell progenitors, and this could hamper recovery from stroke. The authors investigate an experimental model of stroke and show that HSD induces innate immune memory in the mice despite the withdrawal of salt from the diet prior to induction of the stroke. Moreover, the authors were able to demonstrate that this effect can be reproduced by transplantation of bone marrow from HSD-fed mice. The data presented are novel and demonstrate a new effect of HSD. The experiments are well performed and the study is clearly written.

Comments:

1. If I understand correctly the ICH model, the authors inject a predetermined amount of autologous blood and assess hematoma volume. The larger hematoma in the HSD mice reflect a poorer function of the reparative MDMs or a smaller number of cells accumulated at the site of the hematoma (Fig.2A)? These would be in principle two possible distinct mechanisms.
2. The authors show reduced oxphos, but what about glycolysis, was that influenced by HSD?
3. The authors performed transcriptional studies in the HPSCs. Can the authors speculate whether these changes will be present in CMPs and GMPs as well?
4. Do the authors believe that NR4a1 has a role in trained immunity induced by HSD alone, or other stimuli as well (e.g. BCG vaccination)?

Referee #2:

High salt diet (HSD) has profound effects on a variety of systems, including the immune system. This paper shows that HSD (8% NaCl) in male mice exacerbates pathological and functional outcomes in models of intracerebral hemorrhage (ICH) (autologous blood or collagenase injection) and focal ischemic injury (middle cerebral artery occlusion). Examination of myeloid cells within the brain indicated a reduction of CD36 and CD206, interpreted as a loss of reparative phenotype of these cells. In vitro studies provided evidence of reduced CD206 and CD36 expression and erythrocyte engulfment in peritoneal macrophages, as well as reduced energy metabolism markers (OXPHOS) in bone marrow (BM) derived cells. BM chimera experiments indicated that BM from HSD mice transplanted to mice fed a normal diet (ND) conferred worse ICH outcome. BM progenitor cell studies suggested increased myelopoiesis and inflammation in HSD, which subsided after HSD was discontinued. However, BM chimera experiments showed that HSD BM transplant was still able to worsen ICH outcome after inflammation subsided. Bulk RNAseq of HSD hematopoietic stem and progenitor cells (HSPG) revealed a large number of transcriptomic changes including suppression of NR4A transcription factors. In mice fed a ND deletion of NR4A in myeloid cells phenocopied the worse ICH outcome of mice fed HSD. Lentiviral overexpression of NR4A in BM derived cells exposed to high salt in vitro restored TREM2 and apoe expression, suggesting restoration of a reparative phenotype. In vivo, administration of the NR4A activation celastrol ameliorated ICH outcome in HSD mice. It is concluded that HSD-induces an innate immune memory leading to persistent dysregulated inflammatory responses involving NR4a1, which could also be a therapeutic target.

This study is of interest because ICH-mediated injury is less well understood than ischemic injury and a better understanding of risk factors and novel therapeutic opportunities would be welcome advances in the field. The use of two models of ICH as well as focal cerebral ischemia broadens the potential impact of the findings. However, other aspects of the study raise concerns.

1. The assessment of the ICH injury is confusing. In some studies, it was done at 7 days, in which case HSD increased hematoma volume. This finding is interpreted to indicate reduced clearance, but the evidence supporting this statement is unclear. The fact that there is reduced CD206 and CD36 expression does not demonstrate reduced clearance by these cells in vivo. More conclusive in vivo evidence of reduced clearance attributable to these cells needs to be provided.

2. In other mice the hematoma volume was assessed at 3 days and it is stated that no difference in volumes were observed, but no quantification is provided. More quantitative assessment of hematoma volume is needed at all time points.

3. CD206 is a marker of border associated macrophages and not microglia (Masuda et al, Nature 2022). Therefore, the molecular changes cannot be attributed to microglia and data pertaining to this specific cell type need to be provided.

4. The in vitro experiments in which thrombin is defined as ICH mimic are problematic and add little to the in vivo data.

5. The in vitro experiments in which BM cells are incubated in a high salt environment are also problematic. What is the evidence that the BM is exposed to high salt and that the observed changes are attributable to it?

6. The experiments with celastrol are difficult to interpret. First, as noted in the discussion, this is multifunctional drug with a wide variety of antioxidant, anti-inflammatory and neuroprotective properties which cannot be specifically ascribed to NR4A activation. Second, the drug was given only to mice fed HSD and to rule out HSD-independent effects the drug should be administered also to ND mice.

7. The chimera experiments are of potential interest. However, experiments in which the BM of ND mice is transplanted into HSD mice are needed to substantiate the contention that BM reprogramming is the critical factor in the ICH phenotype.

8. In the description of the focal ischemia experiments it is stated that "...the right middle cerebral artery was ligated with 10-0 nylon suture and the right common carotid artery was occluded with aneurysm clips. The cerebral blood supply was restored after 40 min of MCAO". How can the blood supply be restored to an occluded middle cerebral artery?

9. HSD for administered for 2 weeks, 4 weeks, 8 weeks. What was the rationale for the differences in HSD exposure?

10. Mechanistically, the question remains how HSD reprograms the BM and the molecular bases of such reprogramming.

Referee #3:

After reviewing the manuscript entitled "Trained immunity induced by high salt diet impedes stroke recovery" I appreciate the research you have conducted. However, I have some major concerns that need to be addressed. Please find my feedback below.

1. In Figure 1F, the image presented for numbers of NeuN+ cells in the HSD group appears to be blurry. I suggest that the authors consider using a different representative figure to present it.

2. Authors should provide a more detailed description of the importance of the relevant indicators, such as the Neurologic score, hindlimb abduction test, and Corner turn test, which show significant differences in Figure 1E during the recovery phase, in the Results section. Additionally, the experimental methods used for these tests should be described in greater detail as well.

3. Authors should address two issues related to Figure EV2. Firstly, the gating strategies presented in Figure EV2 did not rule out the lineage markers (CD3/CD19/Ly6G), which is however mentioned in the Materials and Methods section. Secondly, the authors only gated on Ly6C+CD11b+ double-positive cells, which may overlook changes in some macrophage subtypes since not all macrophages express CD11b, and the proportion of CD11b+ cells that are macrophages can vary depending on the infection status. Thus, the authors should consider gating on additional macrophage markers or providing a clear rationale for their gating strategy.

4. The authors' observation of a lower percentage of HSD-induced reparative phenotypes in MDMs, rather than microglia, are noteworthy. However, in Figure 2c, while investigating the expression of proinflammatory genes, such as Tnf, Tspo, and epigenetic regulator genes, Hdac1 and Hdac2, in brain-sorted microglia, the authors did not perform the same analyses on MDMs, which showed significant changes in reparative phenotypes. Given the crucial role of epigenetic regulation in trained immunity, it would be valuable for the authors to conduct additional experiments on MDMs to investigate epigenetic regulation, as well as cytokine expression (such as IL-1b and IL-1Ra), which are crucial for promoting training effects in response to factors like BCG.

5. In Figure 3C, the authors suggest that the exclusion of more proinflammatory MDM/microglial phenotypes during the acute phase could be based on the non-difference of MDM in CD45+ population between the ND and HSD groups and the absence of aggravated acute brain hemorrhage seen on day 3. However, in this figure, the authors only analyzed membrane-bound form of TNF without examining soluble TNF and other cytokines closely related to trained immunity, such as IL-1beta, IL-1Ra, IL-6, and IL-32, which is somewhat premature. Besides, in Figure 3D, the non-significant increase in membrane-bound form of TNF in the HSD group may be due to the small sample size. Therefore, I recommend that the authors include analyses of other cytokines in

their study and perform additional experiments with a larger sample size to validate their findings.

6. To strengthen their findings, the authors should present more compelling evidence for the trend of increased CD36+ pMFs from day 0 to day 3 and day 7 after thioglycollate injection in Figure EV3B. Without a Day 0 control, it is difficult to draw definitive conclusions about the observed trend. Furthermore, the authors should discuss the interrelationship between CD36 and CD206 markers. Given that both markers are important for reparative processes, it would be expected that their expression patterns are similar. Therefore, the authors should provide more insights and explanations about the potential correlation between these two markers.

7. Based on the data presented in Fig EV3, which demonstrated a widespread reduction in reparative macrophages regardless of the type of inflamed tissue, the authors thus proposed that the HSD-induced phenotypic alterations in macrophages may originate from bone marrow progenitors. However, it may be too early to suggest that the training effects originate from bone marrow progenitors based on the data by far shown in Fig EV3 as peripheral blood monocytes or other peritoneal resident macrophages (PRMs) residing in the peritoneal cavity could also be trained by HSD. Therefore, the authors should consider other potential sources of trained macrophages and acknowledge these possibilities in this context.

8. Based on the data presented in Fig 4 A to D, the authors suggest that feeding HSD to mice can lead to a decrease in the ability of their BM cells to perform OXPHOS, as evidenced by reduced basal respiration, ATP production, and maximal respiration compared to ND BMDMs. This decrease in OXPHOS is accompanied by a downregulation of some reparative genes. However, I have the following questions regarding these findings:

a) Have the authors considered the temporal relationship between the decrease in OXPHOS and the downregulation of reparative phenotype genes? It would be helpful to determine which one is the primary cause and how they are linked.
b) I am confused about the relationship with NR4A1. Can the authors clarify this further, as no direct link has been found between NR4A1 and OXPHOS?
c) Investigating the relationship between the decrease in OXPHOS capacity and key TCA cycle metabolites would enhance the study. Can the authors test some of these metabolites? The TCA cycle and OXPHOS are tightly coordinated, and understanding their relationship would provide valuable insights.
d) Have the authors considered which metabolic pathway plays a main role in producing energy in reparative macrophages or M2-like macrophages?
e) From Fig 4C, it appears that feeding HSD to mice for only two weeks and then differentiating their BM cells into BMDMs in vitro for 7 days is sufficient to observe changes in reparative phenotypes. Can the authors clarify how long it actually takes to achieve these training effects caused by HSD?

9. Regarding the bone marrow-chimeric mice model presented in Fig 4E to I, where ND hosts were reconstituted with bone marrow from mice fed with HSD for 4 weeks, I have a question for the authors. This experiment is critical as it represents the training effects occurring at the level of BM cells. However, I would like to clarify the following: During the 8-week recovery following transplantation, if I am not mistaken, the mice were fed with ND. Nevertheless, the data indicated that even though the mice returned to a normal salt diet for 8 weeks, they were still unable to reverse the training effects. According to my understanding, the effects of trained immunity have a certain time limit and are reversible, such as those caused by BCG. Could the authors provide further clarification on this phenomenon?

10. It would be better if the authors could label the hematoma region in the other immunofluorescence images in the same way as Figure 4.

11. The initial hypothesis made by the authors in Figure 1A to E suggests that the effect of HSD primarily impacts long-term stroke recovery rather than exacerbating acute brain hemorrhage. This hypothesis was based on the observation that the main effects were seen on Day 7 rather than Day 3 after intracerebral hemorrhage. However, the cylinder test in Figure 4F, 5B, and 7C showed significant functional differences as early as Day 3. Please provide further clarification on this discrepancy.

12. In Figure 6A, the authors identified 289 differentially expressed genes (DEGs) in HSD HSPCs and used IPA analysis to link the training effects induced by HSD to glycolysis and cholesterol biosynthesis. This finding is consistent with the earlier observation that HSD reduces OXPHOS capacity. However, the authors did not provide a comprehensive discussion or organization of the genes related to glycolysis. Instead, they focused on the NR4A family. If the authors insist on pursuing this direction, they should provide more literature support for the importance of the NR4A family in glycolysis and perform additional experiments to further verify their findings.

13. it would be beneficial to perform a Luciferase Reporter Assay to confirm the effect of HSD on NR4a1's transcriptional activity.

14. If the authors consider NR4A1 to be a key factor in the HSD-induced training effects, it would be appropriate to analyze the promoter methylation status of NR4A1.

15. The authors have used lentiviral infection to overexpress Nr4a1 in BM cells and have observed successful restoration of Trem2 and Apoe expression in BMDMs differentiated under high-sodium conditions. However, changes in surface markers

alone may not fully represent functional changes. Therefore, it is recommended that the authors consider performing additional tests such as erythrophagocytosis, or creating bone marrow-chimeric mice through overexpression of NR4a1 in BM cells, to investigate whether high levels of NR4a1 can mitigate the harmful effects of HSD.

August 23, 2023

Dear Dr. Breiling,

Thank you for your thoughtful consideration of our manuscript "Trained immunity induced by high salt diet impedes stroke recovery" (EMBOR-2023-57164V2). We appreciate the valuable comments of three referees and the opportunity to address the key points raised by the referees. We believe the revised manuscript that accompanies this letter satisfactorily addresses the referees' concerns. Furthermore, we have made significant modifications to the manuscript and figures, which are outlined below:

- First, we have revised the language and more explicitly stated our results. We also revised and reorganized the main figures and EV figures to better present our work. All raw data and datasets that were used to generate the results have been deposited as an online resource which will allow readers access to additional details from the experiments.

- Second, we have added new experiments to further strengthen our findings, which include the following: 1) We performed additional flow cytometry analysis by adding more animals and phenotype markers (IL-1α, IL-6, MerTK, and ARG1) to support that HSD does not aggravate proinflammatory MDM/microglial phenotype during the acute phase of ICH, but specifically dampens the reparative activation and function of MDMs at the recovery phase; 2) we confirmed that HSD induces the core signature of trained immunity – increased glycolytic capacity in the bone marrow (BM) cells; 3) we performed rescue experiments by transplanting BM of ND donors into HSD mice which further substantiate our conclusion that BM reprograming is the critical factor in the HSD-induced ICH phenotype; and 4) we treated *Nr4a1* cKO mice with celastrol after ICH induction to confirm the beneficial effects of celastrol on HSD mice are indeed mediated through NR4a1.

- Third, *in vivo* and *in vitro* erythrophagocytosis were employed as functional assays to support that MDMs reduce their phagocytosis ability in the HSD ICH brain and that high levels of NR4a1 can mitigate the harmful effects of HSD.

- A total of additional 114 animals was used to complete the experiments for revision.

In summary, we found the referees' comments to be highly constructive and readily addressable. Below, we have provided a point-by-point response to the referees' comments. We have also included an updated version of the manuscript and figures for your consideration.

Warmest regards,

Che-Feng Chang, PhD

Referee #1:

This is an interesting study that investigates the original hypothesis that a high-salt diet (HSD) induces trained immunity through long-term changes in hematopoietic stem cell progenitors, and this could hamper recovery from stroke. The authors investigate an experimental model of stroke and show that HSD induces innate immune memory in the mice despite the withdrawal of salt from the diet prior to induction of the stroke. Moreover, the authors were able to demonstrate that this effect can be reproduced by transplantation of bone marrow from HSD-fed mice. The data presented are novel and demonstrate a new effect of HSD. The experiments are well performed and the study is clearly written.

Thank you for your support of our work.

Comments:

1. If I understand correctly the ICH model, the authors inject a predetermined amount of autologous blood and assess hematoma volume. The larger hematoma in the HSD mice reflect a poorer function of the reparative MDMs or a smaller number of cells accumulated at the site of the hematoma (Fig.2A)? These would be in principle two possible distinct mechanisms.

Thank you for this comment. We agree that these are possibly two distinct mechanisms. In our study, we observed no difference in the total number of infiltrating MDMs between ND and HSD groups (Figure 2G and 5F). Additionally, the number of reparative MDMs was significantly reduced after HSD (Figure 2A and 5E) while the number of proinflammatory MDMs remained comparable between the two groups (Figure 2H). Given these results, we reasoned that the larger hematoma and worse ICH outcomes in the HSD mice were more likely the result of reduced activation of the reparative MDMs.

2. The authors show reduced oxphos, but what about glycolysis, was that influenced by HSD?

We appreciate this suggestion and have included additional data on the effect of HSD on glycolysis in the bone marrow (BM) cells (Figure EV3C-D). We found that both 4- and 8-week HSD BM cells displayed significantly increased glycolysis, which agrees with our results of bulk RNAseq and findings of dampened OXPHOS in these HSD BM cells.

3. The authors performed transcriptional studies in the HPSCs. Can the authors speculate whether these changes will be present in CMPs and GMPs as well?

We speculate it is likely that similar changes would be observed in CMPs and GMPs. We observed that 8-week HSD increased the percentage of GMPs (Figure 4B). While it is evident that HSD induces GMP proliferation and a concomitant systemic inflammatory response (increased $CD11b^{+}Ly6C^{hi}$ proinflammatory monocytes), what metabolic and transcriptional changes may be in the CMPs and GMPs remain to be investigated in future studies.

4. Do the authors believe that NR4a1 has a role in trained immunity induced by HSD alone, or other stimuli as well (e.g. BCG vaccination)?

Given the results of our study and others, we theorize that the NR4a1-mediated mechanism of trained immunity in HSPCs is unique to HSD. While we cannot rule out the possibility that other stimuli may also employ the same mechanism, various studies in recent years show that different training agents such as β-glucan, BCG vaccination, LPS, and heme employ distinct mechanisms of trained immunity in HSPCs (reviewed in De Zuani and Frič, Frontiers in Immunology, 2022; Bekkering et al, Annual Review of Immunology, 2021). On the other hand, innate immune reprogramming in GMPs following a Western diet regime was shown to be mediated by NLRP3 activation and subsequent release of IL-1 (Christ et al, Cell, 2018). It will be interesting to investigate in future studies whether NR4a1-mediated trained immunity can be found following other microbial and nonmicrobial stimuli. We have now included this information in the revised discussion.

Referee #2:

High salt diet (HSD) has profound effects on a variety of systems, including the immune system. This paper shows that HSD (8% NaCl) in male mice exacerbates pathological and functional outcomes in models of intracerebral hemorrhage (ICH) (autologous blood or collagenase injection) and focal ischemic injury (middle cerebral artery occlusion). Examination of myeloid cells within the brain indicated a reduction of CD36 and CD206, interpreted as a loss of reparative phenotype of these cells. In vitro studies provided evidence of reduced CD206 and CD36 expression and erythrocyte engulfment in peritoneal macrophages, as well as reduced energy metabolism markers (OXPHOS) in bone marrow (BM) derived cells. BM chimera experiments indicated that BM from HSD mice transplanted to mice fed a normal diet (ND) conferred worse ICH outcome. BM progenitor cell studies suggested increased myelopoiesis and inflammation in HSD, which subsided after HSD was discontinued. However, BM chimera experiments showed that HSD BM transplant was still able to worsen ICH outcome after inflammation subsided. Bulk RNAseq of HSD hematopoietic stem and progenitor cells (HSPG) revealed a large number of transcriptomic changes including suppression of NR4A transcription factors. In mice fed a ND deletion of NR4A in myeloid cells phenocopied the worse ICH outcome of mice fed HSD. Lentiviral overexpression of NR4A in BM derived cells exposed to high salt in vitro restored TREM2 and apoe expression, suggesting restoration of a reparative phenotype. In vivo, administration of the NR4A activation celastrol ameliorated ICH outcome in HSD mice. It is concluded that HSD-induces an innate immune memory leading to persistent dysregulated inflammatory responses involving NR4a1, which could also be a therapeutic target.

This study is of interest because ICH-mediated injury is less well understood than ischemic injury and a better understanding of risk factors and novel therapeutic opportunities would be welcome advances in the field. The use of two models of ICH as well as focal cerebral ischemia broadens the potential impact of the findings. However, other aspects of the study raise concerns.

Thank you for your enthusiasm for our work.

1. The assessment of the ICH injury is confusing. In some studies, it was done at 7 days, in which case HSD increased hematoma volume. This finding is interpreted to indicate reduced clearance, but the evidence supporting this statement is unclear. The fact that there is reduced CD206 and CD36 expression does not demonstrate reduced clearance by these cells in vivo. More conclusive in vivo evidence of reduced clearance attributable to these cells needs to be provided.

We agree with the reviewer that the reduced CD206 and CD36 expression in MDMs alone does not necessarily support a decline in phagocytic function of MDMs *in vivo*. We have therefore performed additional experiments where we injected PKH-26-labeled erythrocytes to induce ICH in both ND and HSD mice (Figure 2B). We then measured the erythrocyte fluorescence signal in MDMs using flow cytometry and observed that the percentage of MDMs that have engulfed PKH-26-erythrocytes was significantly lower in HSD mice when compared to the ND mice, suggesting a reduced capacity to phagocytose erythrocytes and to clear hematoma in the HSD MDMs.

2. In other mice the hematoma volume was assessed at 3 days and it is stated that no difference in volumes were observed, but no quantification is provided. More quantitative assessment of hematoma volume is needed at all time points.

We have now quantified the hematoma volume and included this data in Figure 2F.

3. CD206 is a marker of border associated macrophages and not microglia (Masuda et al, Nature 2022). Therefore, the molecular changes cannot be attributed to microglia and data pertaining to this specific cell type need to be provided.

We agree with the reviewer that while CD206 was traditionally accepted as a marker of reparative and phagocytic tissue-resident macrophages (A-Gonzalez et al, Journal of Experimental Medicine, 2017), this observation remains controversial and may no longer be accurate. As requested, we have included an assessment of additional molecular signatures in microglia, including MerTK and ARG1 (Figure 2C). In agreement with our previous results, we observed no difference in percentages of MerTK- and ARG1-positive microglia between ND and HSD mice.

4. The in vitro experiments in which thrombin is defined as ICH mimic are problematic and add little to the in vivo data.

Thrombin has been used to mimic ICH-induced injury both *in vivo* and *in vitro* in 50 plus papers (Ye et al, Translational Stroke Research, 2021; Chang et al, Journal of Clinical Investigation, 2018; Taylor et al, Journal of Clinical Investigation, 2016; Babu et al, Neurosurgical Focus, 2012; Reviewed in Hua et al, Stroke, 2007). Given the plethora of studies from our group and others, we believe thrombin is an adequate stimulus to mimic ICH *in vitro*. Using thrombin to produce an *in vitro* model of ICH allowed us to validate our *in vivo* results and test the idea that HSD alters the properties of BM cells and their mature progeny. We have included additional references to support this experimental strategy in the revision.

5. The in vitro experiments in which BM cells are incubated in a high salt environment are also problematic. What is the evidence that the BM is exposed to high salt and that the observed changes are attributable to it?

We understand the reviewer's concerns that incubating BM cells in a high sodium environment may not truly mimic feeding a high salt diet to mice *in vivo*. Previous studies have described an increase in interstitial sodium in both humans and experimental animals following HSD, which has been calculated to be approximately 40 mM greater than that measured in plasms (Titze, Curr Opin Nephrol Hypertens., 2014; Kopp et al, Hypertension, 2013; Wiig et al, Journal of Clinical Investigation, 2013). Several studies have also elegantly demonstrated that NaCl affects the activation and function of immune cells by directly exposing these cells to media with an increased concentration of NaCl (Matthias et al, Journal of Clinical Investigation, 2020; Binger et al, Journal of Clinical Investigation, 2015; Wu et al, Nature, 2013). Given the results of ours and others, we feel this is currently the most appropriate way to mimic the *in vivo* condition of HSD and investigate its effect on BM cells and their progeny *in vitro*. We have included references to support this experimental strategy in the revision.

6. The experiments with celastrol are difficult to interpret. First, as noted in the discussion, this is multifunctional drug with a wide variety of antioxidant, anti-inflammatory and neuroprotective properties which cannot be specifically ascribed to NR4A activation. Second, the drug was given only to mice fed HSD and to rule out HSD-independent effects the drug should be administered also to ND mice.

We understand the reviewer's concerns. To address them, we have included new experiments in which NR4a1 conditional knockout mice (*NR4a1* cKO) were assigned to receive either celastrol or vehicle treatments (Figure EV5B-D). We observed no difference in hematoma volume or the percentages of reparative MDMs between *NR4a1* cKO mice that were given celastrol treatment and those receiving vehicle treatment, suggesting that the effect of celastrol is specific and most likely mediated through NR4a1.

7. The chimera experiments are of potential interest. However, experiments in which the BM of ND mice is transplanted into HSD mice are needed to substantiate the contention that BM reprogramming is the critical factor in the ICH phenotype.

This is an important question. We thank the reviewer for this valuable suggestion and have included additional experiments in which HSD hosts were irradiated and reconstituted with BM from either ND donors or HSD donors (Figure EV4G-K). We found that HSD mice constituted with ND BM had improved functional performance on the cylinder test than those with HSD BM. HSD mice transplanted with ND BM showed smaller hematoma volumes, decreased neuron loss and astrogliosis, as well as elevated numbers of $CD206^{+}IBA1^{+}$ and $MAC2^{+}IBA1^{+}$ cells. Together these data suggest that reconstituting HSD mice with ND BM restored the reparative MDMs in HSD mice and promoted brain rescue and functional recovery. These results support that BM reprogramming is the critical process that determines long-term tissue recovery in HSD-fed animals.

8. In the description of the focal ischemia experiments it is stated that "...the right middle cerebral artery was ligated with 10-0 nylon suture and the right common carotid artery was occluded with aneurysm clips. The cerebral blood supply was restored after 40 min of MCAO". How can the blood supply be restored to an occluded middle cerebral artery?

We apologize for the confusion and have now specified that this is a “transient MCAO (tMCAO) model” in the revised materials and methods. We have also corrected the wording to “...artery was ligated reversibly… occluded reversibly with aneurysm clips” in this section.

9. HSD for administered for 2 weeks, 4 weeks, 8 weeks. What was the rationale for the differences in HSD exposure?

We initially designed our experiments with 8 weeks of HSD based on previously established protocols (Faraco et al, Nature Neuroscience, 2018). In consideration of animal welfare and the time-consuming nature of bone marrow chimeras-related experiments, we have since tested 4-week dietary manipulations and confirmed reduced neurobehavioral recovery and reparative MDMs in HSD mice similar to what we observed with 8 weeks of HSD. Our observations also align with recent studies showing negative effects of 4-week HSD on cerebrovascular diseases in mice (Faraco et al, Nature, 2019; Faraco et al, Nature Neuroscience, 2018). In addition, 2-week HSD is shown to be sufficient to trigger a systemic inflammation in human subjects (Ferguson et al, JCI insight, 2019; Wenstedt et al, JCI insight, 2019) and impact mitochondrial energetics in human peripheral blood mononucleated cell (PBMC)-derived macrophages (Geisberger et al, Circulation, 2021). These findings support our *in vitro* studies that macrophages derived from 2-week HSD-fed mice had reduced reparative gene expressions. While we regret that different durations of HSD were employed, we are confident about the results and that the negative effects on macrophage function and long-term stroke recovery were attributable to HSD.

10. Mechanistically, the question remains how HSD reprograms the BM and the molecular bases of such reprogramming.

Thank you for this comment. We agree with the reviewer that the precise molecular mechanisms through which HSD downregulates NR4a family and metabolically rewires HSPCs remain to be explored. We have included a more in-depth discussion of these limitations in the revised manuscript. Resolving these questions will be interesting for future studies.

Referee #3:

After reviewing the manuscript entitled "Trained immunity induced by high salt diet impedes stroke recovery" I appreciate the research you have conducted. However, I have some major concerns that need to be addressed. Please find my feedback below.

We thank the reviewer for these valuable comments and will now address them in order:

1. In Figure 1F, the image presented for numbers of NeuN+ cells in the HSD group appears to be blurry. I suggest that the authors consider using a different representative figure to present it.

Thank you for this suggestion. We have replaced with a different representative image in Figure 1F.

2. Authors should provide a more detailed description of the importance of the relevant indicators, such as the Neurologic score, hindlimb abduction test, and Corner turn test, which show significant differences in Figure 1E during the recovery phase, in the Results section. Additionally, the experimental methods used for these tests should be described in greater detail as well.

Thank you for this comment. We have added more details to describe what neurobehavioral deficits are reflected by these functional tests in the result section. Additional references have also been provided in the neurobehavioral tests section. The details provided in the material and method section and the references together should satisfy the need of readers interested in performing these tests.

3. Authors should address two issues related to Figure EV2. Firstly, the gating strategies presented in Figure EV2 did not rule out the lineage markers (CD3/CD19/Ly6G), which is however mentioned in the Materials and Methods section. Secondly, the authors only gated on Ly6C+CD11b+ double-positive cells, which may overlook changes in some macrophage subtypes since not all macrophages express CD11b, and the proportion of CD11b+ cells that are macrophages can vary depending on the infection status. Thus, the authors should consider gating on additional macrophage markers or providing a clear rationale for their gating strategy.

The possible contribution of CD11b negative macrophages after stroke is a very interesting question. We agree with the reviewer that this cell population could be an important player in inflamed tissue after infection. However, we do not believe that CD11b negative macrophages are responsible for the results we observed in the current study for the following reasons. We and others have previously shown that $CD45^{hi}CD11b^{+}$ and $CD45^{hi}CD11b^{+}Ly6C^{+}$ MDMs are the major cell populations that aid in hematoma clearance after ICH (Zhou et al, Journal of Advanced Research, 2023; Chang et al, Stroke, 2021; Xu et al, Proc Natl Acad Sci U S A., 2020; Chang et al, Journal of Clinical Investigation, 2018; Reviewed in Frontiers in Neurology, 2023). In addition, based on our single-cell RNA-seq studies, we have yet observed a CD11b negative macrophage population in the human and mouse ICH brains (Goods et al, JCI insight, 2021; Askenase et al, Science Immunology, 2021). We, therefore, believe that the delayed hematoma clearance and impeded brain recovery in the HSD mice are mainly caused by the reduced reparative activation of the $CD45^{hi}CD11b^{+}Ly6C^{+}$ MDMs. We have now specified the major lineage markers used and their dump gate in the gating strategies in Appendix S1.

4. The authors' observation of a lower percentage of HSD-induced reparative phenotypes in MDMs, rather than microglia, are noteworthy. However, in Figure 2c, while investigating the expression of proinflammatory genes, such as Tnf, Tspo, and epigenetic regulator genes, Hdac1 and Hdac2, in brain-sorted microglia, the authors did not perform the same analyses on MDMs, which showed significant changes in reparative phenotypes. Given the crucial role of epigenetic regulation in trained immunity, it would be valuable for the authors to conduct additional experiments on MDMs to investigate epigenetic regulation, as well as cytokine expression (such

as IL-1b and IL-1Ra), which are crucial for promoting training effects in response to factors like BCG.

We agree that significant changes in MDM reparative phenotype in HSD mice merit a more in-depth functional investigation. We have since performed an *in vivo* erythrophagocytosis assay using the autologous blood injection model in which we fluorescently labeled the erythrocytes prior to injection. We quantified MDMs that had engulfed erythrocytes at day 7 after ICH by flow cytometry and found that MDMs from HSD brains had reduced capacity to phagocytose erythrocytes. This functional evidence along with our previous finding showing decreased percentages of $CD36^+$ and $CD206^+$ reparative MDMs in the HSD hemorrhagic brain support that MDMs had reduced reparative phenotypes in the HSD brain. Additionally, we have measured IL-1α and IL-6 expression in the MDMs and microglia by flow cytometry as requested. In agreement with our previous results, we observed no difference in percentages of IL-1α- and IL-6-positive MDMs/microglia between ND and HSD mice. These data are now presented in Figures 2B, H, and I. We also agree with the reviewer that epigenetic regulation is one of the crucial factors for inducing trained immunity. While both metabolic reprogramming and epigenetic modification are critical mechanisms through which innate immune memory can be generated (Zhang et al, Nature Metabolism, 2021; Sohrabi et al, Frontiers in Immunology, 2020; Reviewed in Bekkering et al, Annual Review of Immunology, 2021; Riksen and Netea, Molecular Aspects of Medicine, 2020), we decided to focus on understanding whether HSD triggers trained immunity through metabolic reprogramming of BM cells in this study. Understanding the epigenetic changes that underlie HSD-induced trained immunity would be valuable in future studies. As such, we have added a section to address this limitation and included relevant future directions in the revised discussion.

5. In Figure 3C, the authors suggest that the exclusion of more proinflammatory MDM/microglial phenotypes during the acute phase could be based on the non-difference of MDM in CD45+ population between the ND and HSD groups and the absence of aggravated acute brain hemorrhage seen on day 3. However, in this figure, the authors only analyzed membrane-bound form of TNF without examining soluble TNF and other cytokines closely related to trained immunity, such as IL-1beta, IL-1Ra, IL-6, and IL-32, which is somewhat premature. Besides, in Figure 3D, the non-significant increase in membrane-bound form of TNF in the HSD group may be due to the small sample size. Therefore, I recommend that the authors include analyses of other cytokines in their study and perform additional experiments with a larger sample size to validate their findings.

We measured intracellular cytokine levels by first adding brefeldin A to trap cytokine production within monocytes during sample preparation and then performed fixation, permeabilization, and intracellular staining for flow cytometry. Therefore, the cytokine levels presented in this figure are from both the membrane-bound and intracellular forms. We have now performed additional experiments by including more animals and cytokines (IL-1α- and IL-6). We observed no difference in percentages of $MHC\text{-}II^+$, $CD86^+$, TNF^+, IL-$1\alpha^+$, and IL-6^+ proinflammatory MDMs and microglia between ND and HSD mice on day 3 after ICH. These data are now presented in Figures 2H and I.

6. To strengthen their findings, the authors should present more compelling evidence for the

trend of increased CD36+ pMFs from day 0 to day 3 and day 7 after thioglycollate injection in Figure EV3B. Without a Day 0 control, it is difficult to draw definitive conclusions about the observed trend. Furthermore, the authors should discuss the interrelationship between CD36 and CD206 markers. Given that both markers are important for reparative processes, it would be expected that their expression patterns are similar. Therefore, the authors should provide more insights and explanations about the potential correlation between these two markers.

We thank the reviewer for this comment and would like to provide additional contexts. Given that the ultimate goal of this experiment is to test whether HSD similarly impacts macrophage reparative functions in the peripheral organs as it does in the ICH brain, we assessed peritoneal macrophage reparative activation between ND and HSD mice. In agreement with a previous study (Bosurgi et al, Science, 2017), we observed that peritoneal macrophages increased expression of reparative markers CD36 and CD206 from days 3 to 7 after thioglycollate injection, suggesting an alternative polarization of these cells. We also saw a significant reduction in reparative macrophage phenotype at day 7 in HSD mice, further strengthening that HSD impairs macrophage reparative polarization. We however understand the reviewer's concern and have now included the day 0 data in this figure. The roles of CD36 and CD206 have been reviewed in-depth elsewhere (Reviewed in Gordon, Immunity, 2016). Considering phagocytosis in macrophages is mediated by a large number of receptors, we reasoned that the expression patterns of these two receptors are not necessarily the same. In addition, CD36 and CD206 only serve as markers of reparative and phagocytic macrophages in this study (A-Gonzalez et al, Journal of Experimental Medicine, 2017), we do not wish to distract the readers by comparing the functional difference and potential correlation between these two scavenger receptors here.

7. Based on the data presented in Fig EV3, which demonstrated a widespread reduction in reparative macrophages regardless of the type of inflamed tissue, the authors thus proposed that the HSD-induced phenotypic alterations in macrophages may originate from bone marrow progenitors. However, it may be too early to suggest that the training effects originate from bone marrow progenitors based on the data by far shown in Fig EV3 as peripheral blood monocytes or other peritoneal resident macrophages (PRMs) residing in the peritoneal cavity could also be trained by HSD. Therefore, the authors should consider other potential sources of trained macrophages and acknowledge these possibilities in this context.

Thank you for this suggestion. We agree with the reviewer that it is too early to suggest trained immunity originated from bone marrow based on the data from the peritoneal macrophage experiments. We have removed this statement and edited our language in the result section. We have also acknowledged that HSD may trigger trained tissue-resident macrophages in other organs in the revised discussion.

8. Based on the data presented in Fig 4 A to D, the authors suggest that feeding HSD to mice can lead to a decrease in the ability of their BM cells to perform OXPHOS, as evidenced by reduced basal respiration, ATP production, and maximal respiration compared to ND BMDMs. This decrease in OXPHOS is accompanied by a downregulation of some reparative genes. However, I have the following questions regarding these findings:

Thank you for these comments.

a) Have the authors considered the temporal relationship between the decrease in OXPHOS and the downregulation of reparative phenotype genes? It would be helpful to determine which one is the primary cause and how they are linked.

We agree this is an interesting question to explore. In our current findings, we first observed reduced OXPHOS in the BM cells of HSD-fed mice. This metabolic change persisted in the differentiated BMDMs. In a separate *in vitro* experiment, differentiated BMDMs showed downregulation of reparative genes. These data suggest that HSD triggers a metabolic reprogramming of BM progenitor cells and that their progeny also inherit this property. We speculate that reduction of OXPHOS in the BM cells occurs first. However, it remains unclear whether decreased OXPHOS in the progeny cells precedes downregulation of reparative phenotype genes as our *in vitro* data suggest the two phenomena occur simultaneously. This is further complicated in an *in vivo* environment. Upon arriving at the injured tissue, macrophages constantly probe environmental cues and dynamically shift their phenotypes and metabolic properties (Avraham and Ben-Arosh, Current Opinion in Immunology, 2023; Koncz et al, Frontiers in Immunology, 2023). Our animal studies suggested that MDMs in the HSD animals fail to polarize towards a reparative phenotype, which further impedes tissue repair. We acknowledge that understanding the temporal relationship between OXPHOS and reparative gene expressions could be important. One should also note that changes between metabolic properties and reparative gene expressions that underlie macrophage polarization are intertwined. We do not plan to pursue this topic in the current study but are willing to take the reviewer's suggestion as a potential future work.

b) I am confused about the relationship with NR4A1. Can the authors clarify this further, as no direct link has been found between NR4A1 and OXPHOS?

Previous studies have reported that NR4a1 is widely involved in cell metabolism. Downregulation of NR4a1 reduces OXPHOS in RAW264.7 cells and activates glycolysis and inflammatory pathways in diseases (Koenis et al, Cell Reports, 2018; Hanna et al, Circulation, 2012; Reviewed in Deng et al, Frontier in Oncology, 2022). We have included these additional references in the revised manuscript.

c) Investigating the relationship between the decrease in OXPHOS capacity and key TCA cycle metabolites would enhance the study. Can the authors test some of these metabolites? The TCA cycle and OXPHOS are tightly coordinated, and understanding their relationship would provide valuable insights.
d) Have the authors considered which metabolic pathway plays a main role in producing energy in reparative macrophages or M2-like macrophages?

We will address both comments here. We agree with the reviewer that investigating which TCA cycle metabolites and metabolic pathways mediate OXPHOS and macrophage phenotype changes would be very interesting. These experiments will allow dissection of the molecular bases responsible for HSD-induced alternations in cellular energetics and reparative activation in macrophages. While we regret that we are unable to perform these experiments due to technical

limitations in our lab, we have now included a more in-depth discussion of these constraints in the revised manuscript.

e) From Fig 4C, it appears that feeding HSD to mice for only two weeks and then differentiating their BM cells into BMDMs in vitro for 7 days is sufficient to observe changes in reparative phenotypes. Can the authors clarify how long it actually takes to achieve these training effects caused by HSD?

We agree this is an important question. Previous studies suggest that trained immunity can be triggered as fast as 1 to 5 days after stimulating cells or animals with various immune training agents (Keating et al, Cell Reports, 2020; Sohrabi et al, Frontiers in Immunology, 2020). A two-week HSD has been shown to be sufficient to trigger systemic inflammation in human subjects (Ferguson et al, JCI insight, 2019; Wenstedt et al, JCI insight, 2019) and impact mitochondrial energetics in human peripheral blood mononucleated cell (PBMC)-derived macrophages (Geisberger et al, Circulation, 2021). Our work also indicates that BMDMs derived from BM cells of two-week HSD-fed mice had reduced reparative gene expressions suggesting a training property in these cells. A follow-up study delineating how rapidly systemic changes and trained immunity can be induced following dietary high salt intake would be important for public health concerns. We have now added this discussion to our future direction in the revised manuscript.

9. Regarding the bone marrow-chimeric mice model presented in Fig 4E to I, where ND hosts were reconstituted with bone marrow from mice fed with HSD for 4 weeks, I have a question for the authors. This experiment is critical as it represents the training effects occurring at the level of BM cells. However, I would like to clarify the following: During the 8-week recovery following transplantation, if I am not mistaken, the mice were fed with ND. Nevertheless, the data indicated that even though the mice returned to a normal salt diet for 8 weeks, they were still unable to reverse the training effects. According to my understanding, the effects of trained immunity have a certain time limit and are reversible, such as those caused by BCG. Could the authors provide
further clarification on this phenomenon?

This is an interesting question. A similar phenomenon in which the training property is retained in the BM chimeras and tissue-resident cells for 8-12 weeks has been reported in several studies (Li et al, Cell, 2022; Bhattarai et al, Nature Communications, 2022; Gao et al, Circulation, 2022; Edgar et al, Circulation, 2021; Laval et al, Cell Stem Cell, 2020). While the detailed mechanisms that mediate these observations are unknown, we believe a follow-up study delineating how rapidly HSD induces systemic changes and innate immune memory and how long these changes persist after salt reduction would be meaningful. We have expanded the discussion on this point.

10. It would be better if the authors could label the hematoma region in the other immunefluorescence images in the same way as Figure 4.

Thank you for this suggestion. As the reviewer requested, we have outlined the hematoma region in Figures 1F, 3H, 7A, 8F, and EV4J.

11. The initial hypothesis made by the authors in Figure 1A to E suggests that the effect of HSD

primarily impacts long-term stroke recovery rather than exacerbating acute brain hemorrhage. This hypothesis was based on the observation that the main effects were seen on Day 7 rather than Day 3 after intracerebral hemorrhage. However, the cylinder test in Figure 4F, 5B, and 7C showed significant functional differences as early as Day 3. Please provide further clarification on this discrepancy.

We apologize for the confusion. We would like to first clarify that only day 7 data showed a significant difference in functional performance between ND-ND and ND-HSD groups in Figure 5B. We recognize that despite best efforts to minimize variation by implementing measures such as controlling for inter-rater reliability, randomization, and blinding, the severity of ICH injuries could be slightly variable between different animals. However, we would like to point out that the cylinder test results on day 1 between ND and HSD groups are consistent across figures. We have also shown that initial hematoma volumes were comparable between the ND and HSD groups at acute stage of injury (Figure 2F and G). While we regret that the initial neurologic deficits induced in different studies might be somewhat variable, we are confident of our results showing functional recovery sustainedly impeded in the HSD groups across these experiments. These results further support our hypothesis that HSD delays long-term recovery after ICH.

12. In Figure 6A, the authors identified 289 differentially expressed genes (DEGs) in HSD HSPCs and used IPA analysis to link the training effects induced by HSD to glycolysis and cholesterol biosynthesis. This finding is consistent with the earlier observation that HSD reduces OXPHOS capacity. However, the authors did not provide a comprehensive discussion or organization of the genes related to glycolysis. Instead, they focused on the NR4A family. If the authors insist on pursuing this direction, they should provide more literature support for the importance of the NR4A family in glycolysis and perform additional experiments to further verify their findings.

Thank you for these comments. We narrowed down our focus to the NR4a family for a number of reasons. First, NR4a1 is the most significant DEG based on p-values when comparing ND and HSD HSPCs. We then looked for consistently expressed DEGs in HSD HSPCs with or without ICH induction and found 35 candidates, including the NR4a family (Figure 6G). Previous studies have also shown that NR4a1 modulates OXPHOS, glycolysis, and inflammatory pathways in different contexts of disease (Koenis et al, Cell Reports, 2018; Hanna et al, Circulation, 2012; Reviewed in Deng et al, Frontier in Oncology, 2022). This makes NR4a family a good candidate as our bulk RNA seq analysis has also shown enrichment of pathways correlated to the core signatures of trained immunity including glycolysis and inflammatory responses. We have now provided additional references in the result sections to support our rationale. We have also performed new experiments to measure glycolysis in the ND and HSD BM cells and found that BM cells increased glycolytic capacity after HSD.

13. it would be beneficial to perform a Luciferase Reporter Assay to confirm the effect of HSD on NR4a1's transcriptional activity.
14. If the authors consider NR4A1 to be a key factor in the HSD-induced training effects, it would be appropriate to analyze the promoter methylation status of NR4A1.

Thank you for these suggestions and we will address both comments here. We agree with the reviewer that the manuscript would benefit from performing both of these above assays. Unfortunately, we were not able to find a collaborator who could provide the technical expertise required for these experiments in such a short period of time. However, we believe the inclusion of both gain- and loss-of-function studies using genetic and pharmacological approaches adequately supports our conclusion that HSD-induced negative effects on macrophage functions and ICH outcomes are mediated through NR4a1. While it is outside the scope of the current study, we agree with the reviewer that investigating the epigenetic modifications induced by HSD is an interesting topic and would provide additional insights into how HSD triggers immunological memory in the innate immune cells in future studies. We have added an additional section to address this limitation and included relevant future directions in the revised discussion.

15. The authors have used lentiviral infection to overexpress Nr4a1 in BM cells and have observed successful restoration of Trem2 and Apoe expression in BMDMs differentiated under high-sodium conditions. However, changes in surface markers alone may not fully represent functional changes. Therefore, it is recommended that the authors consider performing additional tests such as erythrophagocytosis, or creating bone marrow-chimeric mice through overexpression of NR4a1 in BM cells, to investigate whether high levels of NR4a1 can mitigate the harmful effects of HSD.

We thank the reviewer for this valuable suggestion and have included additional experiments showing that overexpression of NR4a1 is sufficient to restore engulfment of fluorescently-labeled erythrocytes by BMDMs that were differentiated in the high sodium condition. This data is now presented in Figure 8C.

Dear Dr. Chang

Thank you for the submission of your revised manuscript to our editorial offices. I have already forwarded the reports from the three referees to you that I asked to re-evaluate the study, you will find again below. As you know, referees #1 and #2 are mostly satisfied by the revisions and think that the manuscript should be published in EMBO reports. In contrast, referee #3 indicates remaining issues and states that the manuscript requires further revisions. After going through your preliminary point-by-point response, and also after cross-commenting with the other referees, I ask you to address the remaining points of referees #2 and #3 in a final revised manuscript as indicated in your letter. Please also provide a final p-b-p-response for these.

Moreover, I have these editorial requests:

- Please provide the abstract written in present tense throughout.

- Please make sure that the number "n" for how many independent experiments were performed, their nature (biological versus technical replicates), the bars and error bars (e.g. SEM, SD) and the test used to calculate p-values is indicated in the respective figure legends (for main, EV and Appendix figures) of the final revised manuscript. Please also check that all the p-values are explained in the legend, and that these fit to those shown in the figure. Please provide statistical testing where applicable. Please avoid the phrase 'independent experiment', but clearly state if these were biological or technical replicates. Please also indicate (e.g. with n.s.) if testing was performed, but the differences are not significant. In case n=2, please show the data as separate datapoints without error bars and statistics. See also:
http://www.embopress.org/page/journal/14693178/authorguide#statisticalanalysis

If n<5, please show single datapoints for diagrams. Presently, for some panels n.s. is not indicated (e.g. in Fig. EV1 and EV2B).

- In the Appendix, please add a legend to Appendix Figure S2.

- Appendix Tables S1 and S2 are Datasets. Please upload these as original excel file as dataset files, named Dataset EV1 and Dataset EV2. Please add a legend on the first TAB of the excel file and update their callouts in the manuscript text. Finally, please remove the tables from the Appendix file.

In addition, I would need from you:
- a short, two-sentence summary of the manuscript (not more than 35 words).
- two to four short (!) bullet points highlighting the key findings of your study (two lines each).
- a schematic summary figure as separate file that provides a sketch of the major findings (not a data image) in jpeg or tiff format (with the exact width of 550 pixels and a height of not more than 400 pixels) that can be used as a visual synopsis on our website.

I look forward to seeing the final revised version of your manuscript when it is ready. Please let me know if you have questions regarding the revision.

Best,

Achim Breiling
Senior Editor
EMBO Reports

Referee #1:

The authors responded to my suggestions adequately.

Referee #2:

The authors have done an excellent job at addressing my comments. A minor remaining issue concerns the thrombin experiments. The fact that it is widely used does not attest to its validity and the limitations of the approach should be highlighted in the discussion.

The experiment with added salt to the cultures is also a concern. What is the concentration of NaCl in the environment of the macrophages in vivo (bone marrow, blood and brain)? Without this information the data is difficult to interpret. Again, this is a

limitation that deserves to be mentioned.

Referee #3:

The authors have demonstrated in two experimental intracerebral hemorrhage (ICH) models that a high-salt diet (HSD) exerts significant adverse effects on brain repair after stroke, which are challenging to reverse in the short term as supported by compelling evidence: (I) even when salt intake was reduced in the following week and (II) through the 8-week recovery period for HSD-bone marrow reconstituted mice consuming a normal diet, there was no significant improvement observed in hematoma clearance.

Mechanistically, the authors propose that this effect is attributed to HSD-induced training in hematopoietic stem and progenitor cells (HSPCs), resulting in the downregulation of NR4a family genes and mitochondrial oxidative phosphorylation. However, what particularly captures my interest and simultaneously raises concerns is the observation that this innate immune training selectively impacts on reparative MDMs, as evidenced by reduced CD36 and CD206 expression, without affecting inflammatory responses during the acute phase at day 3 post-ICH.This unique phenomenon appears to challenge the concept of "Trained Immunity," which suggests that training inducers, whether microbial like BCG or non-microbial like oxLDL and uric acid, typically enhance the potential to induce inflammatory responses upon secondary stimulation. Thus, this distinctiveness of HSD-induced training, which causes no effects on inflammatory responses, presents a significant deviation from the existing literature on Trained Immunity so far.

Overall, after the first round of major revisions, the manuscript has significantly improved in terms of completeness. However, the authors have yet to provide a sufficiently satisfactory response to the questions and concerns raised during my initial review. Furthermore, the manuscript falls short of meeting the expected standards for publication in this journal, especially in establishing a robust mechanistic relationship between HSD-induced delayed brain repair, OXPHOS capacity, and the involvement of NR4a family proteins, both in experimental data and within the manuscript's description.

Besides, there are aspects that demand additional improvement:

1. Regarding the immunofluorescence staining of GFAP+ and NeuN+, it is crucial to ensure the precise localization of GFAP within NeuN+ neurons. Failing to do so can pose challenges in reliably identifying true immunopositivity for GFAP within neurons. Therefore, it is strongly recommended to merge these two stainings.

2. As previously mentioned, the observed impact of HSD-induced trained immunity appears to be limited to MDMs, with data excluding the involvement of microglia and HSPC. However, during the acute phase of the ICH model on day 3, it is difficult to be convinced by the fact that no any noticeable impact on inflammatory cytokines within MDMs. Thus, it is worthwhile to investigate a broader range of inflammatory cytokines, both in terms of their serum levels and within MDMs, to provide a more comprehensive understanding of the phenomenon of trained immunity.

3. The manuscript does not provide a clear rationale for the immunofluorescence staining of MAC2+IBA1+ in either the Introduction or the Results sections.

4. Many figure legends lack sufficient explanations and clarity.

5. While the authors conducted bulk RNA sequencing to observe differentially expressed genes (DEGs) in HSPCs derived from bone marrow, it's essential to note that both macrophages and neutrophils are critical components of the hematopoietic niche, and neutrophils also play a significant role in trained immunity. Therefore, it remains unclear why the authors chose to exclusively focus on MDMs, especially considering that a substantial portion of genes are contributed by neutrophils in the bulk RNA seq data.

NATIONAL TAIWAN UNIVERSITY
COLLEGE OF MEDICINE

Department of Physiology/
Graduate Institute of Physiology
10 Floor., No.1, Sec. 1, Ren' ai Rd.,
Zhongzheng Dist., Taipei City 100,
Taiwan (R.O.C.)

TEL: +886-2-23123456
FAX: +886-2-23964350

October 22, 2023

RE: Manuscript EMBOR-2023-57164V2

Point-by-point response to reviewers:

We are very pleased with the overall positive assessment of our work by the reviewers, and we thank them for their helpful suggestions and comments to improve the manuscript. Please find our responses below.

The authors responded to my suggestions adequately.

- Thank you for your support of our work.

The authors have done an excellent job at addressing my comments. A minor remaining issue concerns the thrombin experiments. The fact that it is widely used does not attest to its validity and the limitations of the approach should be highlighted in the discussion. The experiment with added salt to the cultures is also a concern. What is the concentration of NaCl in the environment of the macrophages in vivo (bone marrow, blood and brain)? Without this information the data is difficult to interpret. Again, this is a limitation that deserves to be mentioned.

- Thank you for your enthusiasm for our work. We agree with the reviewer that the *in vitro* stimuli, in some experimental scenarios, may not be able to fully reflect the complexity of the *in vivo* environment, and the above limitations should be brought up in the discussion. We have since added this caveat in our revised discussion.

The authors have demonstrated in two experimental intracerebral hemorrhage (ICH) models that a high-salt diet (HSD) exerts significant adverse effects on brain repair after stroke, which are challenging to reverse in the short term as supported by compelling evidence: (I) even when salt intake was reduced in the following week and (II) through the 8-week recovery period for HSD-bone marrow reconstituted mice consuming a normal diet, there was no significant improvement observed in hematoma clearance. Mechanistically, the authors propose that this effect is attributed to HSD-induced training in hematopoietic stem and progenitor cells (HSPCs), resulting in the downregulation of NR4a family genes and mitochondrial oxidative phosphorylation.

- We appreciate the reviewer pointing out the significance and novelty of our work.

However, what particularly captures my interest and simultaneously raises concerns is the observation that this innate immune training selectively impacts on reparative MDMs, as evidenced by reduced CD36 and CD206 expression, without affecting inflammatory responses during the acute phase at day 3 post-ICH. This unique phenomenon appears to challenge the concept of "Trained Immunity," which suggests that training inducers, whether microbial like BCG or non-microbial like oxLDL and uric acid, typically enhance the potential to induce inflammatory responses upon secondary stimulation. Thus, this distinctiveness of HSD-induced training, which causes no effects on inflammatory responses, presents a significant deviation from the existing literature on Trained Immunity so far.

- Regarding the concept of "trained immunity," studies have reported that trained immunity can have different functions, either in accelerating inflammatory/anti-inflammatory or attenuating inflammatory processes according to the nature of the training. In some cases, the changes in phenotype expression of trained macrophages do not even fit the concept of classic and alternative macrophage polarization. These works have elegantly demonstrated the heterogeneity of training properties. Our work also revealed that trained innate immunity is not confined to heightened inflammatory responses. While the

exact molecular characteristics of diverse types of trained immunity and their cellular targets remain to be further defined, the divergency and heterogeneity of trained immunity should be acknowledged and are worthy of further investigation for disease treatment.

Here are some of the examples:
Trained immunity in type 2 immune responses. *Mucosal Immunology*. 2022 Jun;15(6):1158-1169.
Helminth imprinting of hematopoietic stem cells sustains anti-inflammatory trained innate immunity that attenuates autoimmune disease. *Journal of Immunology*. 2021 Apr 1;206(7):1618-1630.
Trained immunity modulates inflammation-induced fibrosis. *Nature Communications*. 2019 Dec 11;10(1):5670.
Anti-inflammatory trained immunity mediated by helminth products attenuates the induction of T cell-mediated autoimmune disease. *Frontiers in Immunology*. 2019 May 21:10:1109.

Overall, after the first round of major revisions, the manuscript has significantly improved in terms of completeness. However, the authors have yet to provide a sufficiently satisfactory response to the questions and concerns raised during my initial review. Furthermore, the manuscript falls short of meeting the expected standards for publication in this journal, especially in establishing a robust mechanistic relationship between HSD-induced delayed brain repair, OXPHOS capacity, and the involvement of NR4a family proteins, both in experimental data and within the manuscript's description.

- As the reviewer has nicely summarized in the comments, this study investigated the impact of HSD on long-term stroke outcomes and that mechanistically this process *"...is attributed to HSD-induced training in hematopoietic stem and progenitor cells (HSPCs), resulting in the downregulation of NR4a family genes and mitochondrial oxidative phosphorylation."*

  We first showed that HSD delays hematoma clearance and long-term ICH recovery through attenuating macrophage reparative polarization and functions. The bone marrow chimera and diet reversal studies further demonstrate that this phenomenon is mediated by innate immunological memory induced by HSD. In addition, the reduced OXPHOS and reparative activation in the HSD bone marrow cells and/or bone marrow-derived macrophages are consistent with the aforementioned decreased macrophage reparative functions in the ICH animals. Given that studies indicate NR4a1 modulates macrophage OXPHOS capacity and phenotypic changes, we further employed loss- and gain-of-function studies using NR4a TKO mice, NR4a1 cKO mice, celastrol treatment, and lentivirus-mediated NR4a1 overexpression to demonstrate that NR4a1 is a potential therapeutic target. We believe that our results as well as the supporting literature provided in the manuscript are sufficient to support the overall hypothesis of the current work.

Besides, there are aspects that demand additional improvement:

- We respectfully disagree with the new points raised by the reviewer below. For some, we fail to follow the logic of these additional experiments requested, and for others, we find them unsubstantiated and out of scope of the current study.

1. Regarding the immunofluorescence staining of GFAP+ and NeuN+, it is crucial to ensure the precise localization of GFAP within NeuN+ neurons. Failing to do so can pose challenges in reliably identifying true immunopositivity for GFAP within neurons. Therefore, it is strongly recommended to merge these two stainings.

- We do not see the scientific value of presenting GFAP- and NeuN-positive cells in one single merged image. Immunofluorescence stainings of GFAP and NeuN were performed and used as distinct measurements of two independent pathological outcomes. It is well-known that astrocytes serve as supporting cells in the brain and that they physically surround neurons and provide trophic, metabolic, and structural support to them. And yet, GFAP and NeuN stain for two distinct cell types and do not overlap. As such, we do not understand the reviewer's concern with immunopositivity for GFAP within neurons.

Here are some examples:

Figure 1G, upper row (WT). Brain regulatory T cells suppress astrogliosis and potentiate neurological recovery. Nature. 2019 Jan;565(7738):246-250. PMID 30602786

Extended Data Fig. 2 (uppermost panels). Transcranial amelioration of inflammation and cell death after brain injury. Nature. 2014 Jan 9;505(7482):223-8. PMID: 24317693

2. As previously mentioned, the observed impact of HSD-induced trained immunity appears to be limited to MDMs, with data excluding the involvement of microglia and HSPC. However, during the acute phase of the ICH model on day 3, it is difficult to be convinced by the fact that no any noticeable impact on inflammatory cytokines within MDMs. Thus, it is worthwhile to investigate a broader range of inflammatory cytokines, both in terms of their serum levels and within MDMs, to provide a more comprehensive understanding of the phenomenon of trained immunity.

- As the reviewer has previously requested, in the revision, we have added more animals and performed additional experiments to measure the expression of proinflammatory and trained immunity-related markers MHC-II, CD86, TNF, IL-1α, and IL-6 in both MDMs and microglia. These proteins are well-established indicators of inflammatory macrophages. We observed no difference in percentages of MHC-II$^+$, CD86$^+$, TNF$^+$, IL-1α^+, and IL-6$^+$ proinflammatory MDMs and microglia between ND and HSD mice at day 3 after ICH. On top of that, brain hematoma volumes were comparable between ND and HSD mice at day 3. A separate study also shows that HSD does not aggravate microglial activation in a mouse model of neurodegeneration. Given that these results clearly demonstrate similar initial injury and macrophage inflammatory responses between ND and HSD mice, we respectfully disagree with the reviewer's request to add measurements of even more inflammatory cytokines in MDMs and serum samples. Given that there is a plethora of cytokines, we chose to test our hypothesis using a number of well-established markers that were also indicative of inflammatory functions to both conserve research resources and protect animal welfare.

References:
Trained immunity: reprogramming innate immunity in health and disease. *Annual Review of Immunology*. 2021 Apr 26:39:667-693.
High-salt diet does not boost neuroinflammation and neurodegeneration in a model of α-synucleinopathy. J*ournal of Neuroinflammation*. 2020 Jan 24;17(1):35.
Macrophage Polarization. *Annual Review of Physiology*. 2017 Feb 10:79:541-566.

3. The manuscript does not provide a clear rationale for the immunofluorescence staining of MAC2+IBA1+ in either the Introduction or the Results sections.

- Similar to the other reparative markers CD206, CD36, Arg1, etc. that were used in this study, MAC2 (gelatin-3) is also a well-established marker of macrophage phagocytosis and alternatively activated macrophages, and has been widely used in detecting reparative macrophages in the CNS.

  Review article and an example:
  Galectin-3 (MAC-2) controls phagocytosis and macropinocytosis through intracellular and extracellular mechanisms. *Frontiers in Cellular Neuroscience*. 2022 Oct 5:16:949079.
  Diet-dependent regulation of TGFβ impairs reparative innate immune responses after demyelination. *Nature Metabolism*. 2021 Feb;3(2):211-227.

4. Many figure legends lack sufficient explanations and clarity.

- We believe we have included sufficient information in the figure legends allowing readers to follow the data presented. In accordance with the journal’s instructions for no redundancy with the results section, we have not repeated experimental details in the figure legends per journal guidelines.

5. While the authors conducted bulk RNA sequencing to observe differentially expressed genes (DEGs) in HSPCs derived from bone marrow, it's essential to note that both macrophages and neutrophils are critical components of the hematopoietic niche, and neutrophils also play a significant role in trained immunity. Therefore, it remains unclear why the authors chose to exclusively focus on MDMs, especially considering that a substantial portion of genes are contributed by neutrophils in the bulk RNA seq data.

- This is a completely new point raised by the reviewer and we do not understand where this comes from. In fact, in addition to macrophages and neutrophils, trained immunity has been observed in dendritic cells, NK cells, innate lymphoid cells, and nonimmune cells. Further, we did not observe a significant number of neutrophil-related genes among the DEGs with subsequent bulk RNA-seq analysis. Given that we have provided sufficient data and necessary background information in the introduction and results to support our focus on MDMs, we find the reviewer’s request unsubstantiated and out of scope of the current study.

  References:
  Trained immunity: reprogramming innate immunity in health and disease. *Annual Review of Immunology*. 2021 Apr 26:39:667-693.
  Trained immunity: adaptation within innate immune mechanisms. *Physiological Reviews*. 2023 Jan 1;103(1):313-346.

Sincerely,

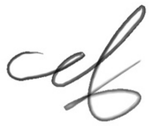

Che-Feng Chang, Ph.D.
Associate Professor
Department and Graduate Institute of Physiology
National Taiwan University College of Medicine

2nd Revision - Editorial Decision 26th Oct 2023

Che-Feng Chang
National Taiwan University College of Medicine
Department and Graduate Institute of Physiology
No. 1, Sec. 1, Jen-Ai Rd.
Room 1038
Taipei, N/A 100
Taiwan

Dear Dr. Chang,

I am very pleased to accept your manuscript for publication in the next available issue of EMBO reports. Thank you for your contribution to our journal.

At the end of this email I include important information about how to proceed. Please ensure that you take the time to read the information and complete and return the necessary forms to allow us to publish your manuscript as quickly as possible.

As part of the EMBO publication's Transparent Editorial Process, EMBO reports publishes online a Review Process File to accompany accepted manuscripts. As you are aware, this File will be published in conjunction with your paper and will include the referee reports, your point-by-point response and all pertinent correspondence relating to the manuscript.

If you do NOT want this File to be published, please inform the editorial office within 2 days, if you have not done so already, otherwise the File will be published by default [contact: emboreports@embo.org]. If you do opt out, the Review Process File link will point to the following statement: "No Review Process File is available with this article, as the authors have chosen not to make the review process public in this case."

Thank you again for your contribution to EMBO reports and congratulations on a successful publication. Please consider us again in the future for your most exciting work.

Yours sincerely,

Achim Breiling
Senior Editor
EMBO Reports

****

THINGS TO DO NOW:

Please note that you will be contacted by Wiley Author Services to complete licensing and payment information. The required 'Page Charges Authorization Form' is available here: https://www.embopress.org/pb-assets/embo-site/er_apc.pdf - please download and complete the form and return to embopressproduction@wiley.com

EMBO Press participates in many Publish and Read agreements that allow authors to publish Open Access with reduced/no publication charges. Check your eligibility: https://authorservices.wiley.com/author-resources/Journal-Authors/open-access/affiliation-policies-payments/index.html

You will receive proofs by e-mail approximately 2-3 weeks after all relevant files have been sent to our Production Office; you should return your corrections within 2 days of receiving the proofs.

Please inform us if there is likely to be any difficulty in reaching you at the above address at that time. Failure to meet our deadlines may result in a delay of publication, or publication without your corrections.

All further communications concerning your paper should quote reference number EMBOR-2023-57164V3 and be addressed to emboreports@wiley.com.

Should you be planning a Press Release on your article, please get in contact with emboreports@wiley.com as early as possible, in order to coordinate publication and release dates.

EMBO Press Author Checklist

Corresponding Author Name: Che-Feng Chang	
Journal Submitted to: EMBO reports	
Manuscript Number: EMBOR-2023-57164V2	

USEFUL LINKS FOR COMPLETING THIS FORM
The EMBO Journal - Author Guidelines
EMBO Reports - Author Guidelines
Molecular Systems Biology - Author Guidelines
EMBO Molecular Medicine - Author Guidelines

Reporting Checklist for Life Science Articles (updated January
This checklist is adapted from Materials Design Analysis Reporting (MDAR) Checklist for Authors. MDAR establishes a minimum set of requirements in transparent reporting in the life sciences (see Statement of Task: 10.31222/osf.io/9sm4x). Please follow the journal's guidelines in preparing your
Please note that a copy of this checklist will be published alongside your article.

Abridged guidelines for figures
1. Data
The data shown in figures should satisfy the following conditions:
- the data were obtained and processed according to the field's best practice and are presented to reflect the results of the experiments in an accurate and unbiased manner.
- ideally, figure panels should include only measurements that are directly comparable to each other and obtained with the same assay.
- plots include clearly labeled error bars for independent experiments and sample sizes. Unless justified, error bars should not be shown for technical
- if n<5, the individual data points from each experiment should be plotted. Any statistical test employed should be justified.
- Source Data should be included to report the data underlying figures according to the guidelines set out in the authorship guidelines on Data

2. Captions
Each figure caption should contain the following information, for each panel where they are relevant:
- a specification of the experimental system investigated (eg cell line, species name).
- the assay(s) and method(s) used to carry out the reported observations and measurements.
- an explicit mention of the biological and chemical entity(ies) that are being measured.
- an explicit mention of the biological and chemical entity(ies) that are altered/varied/perturbed in a controlled manner.
- the exact sample size (n) for each experimental group/condition, given as a number, not a range;
- a description of the sample collection allowing the reader to understand whether the samples represent technical or biological replicates (including how many animals, litters, cultures, etc.).
- a statement of how many times the experiment shown was independently replicated in the laboratory.
- definitions of statistical methods and measures:
  - common tests, such as t-test (please specify whether paired vs. unpaired), simple χ2 tests, Wilcoxon and Mann-Whitney tests, can be unambiguously identified by name only, but more complex techniques should be described in the methods section;
  - are tests one-sided or two-sided?
  - are there adjustments for multiple comparisons?
  - exact statistical test results, e.g., P values = x but not P values < x;
  - definition of 'center values' as median or average;
  - definition of error bars as s.d. or s.e.m.

Please complete ALL of the questions below.
Select "Not Applicable" only when the requested information is not relevant for your study.

Materials

**Newly Created Materials**	**Information included in the manuscript?**	**In which section is the information available?** (Reagents and Tools Table, Materials and Methods, Figures, Data Availability Section)
New materials and reagents need to be available; do any restrictions apply?	Not Applicable	

**Antibodies**	**Information included in the manuscript?**	**In which section is the information available?** (Reagents and Tools Table, Materials and Methods, Figures, Data Availability Section)
For **antibodies** provide the following information:<br>- Commercial antibodies: RRID (if possible) or supplier name, catalogue number and or/clone number<br>- Non-commercial: RRID or citation	Yes	Materials and Methods

**DNA and RNA sequences**	**Information included in the manuscript?**	**In which section is the information available?** (Reagents and Tools Table, Materials and Methods, Figures, Data Availability Section)
**Short novel DNA or RNA including primers, probes**: provide the sequences.	Not Applicable	

**Cell materials**	**Information included in the manuscript?**	**In which section is the information available?** (Reagents and Tools Table, Materials and Methods, Figures, Data Availability Section)
**Cell lines:** Provide species information, strain. Provide accession number in repository **OR** supplier name, catalog number, clone number, and/**OR** RRID.	Not Applicable	
**Primary cultures:** Provide species, strain, sex of origin, genetic modification status.	Yes	Materials and Methods
Report if the cell lines were recently **authenticated** (e.g., by STR profiling) and tested for mycoplasma contamination.	Not Applicable	

**Experimental animals**	**Information included in the manuscript?**	**In which section is the information available?** (Reagents and Tools Table, Materials and Methods, Figures, Data Availability Section)
**Laboratory animals or Model organisms:** Provide species, strain, sex, age, genetic modification status. Provide accession number in repository **OR** supplier name, catalog number, clone number, **OR** RRID.	Yes	Materials and Methods
**Animal observed in or captured from the field:** Provide species, sex, and age where possible.	Yes	Materials and Methods
Please detail **housing and husbandry conditions**.	Yes	Materials and Methods

**Plants and microbes**	**Information included in the manuscript?**	**In which section is the information available?** (Reagents and Tools Table, Materials and Methods, Figures, Data Availability Section)
**Plants:** provide species and strain, ecotype and cultivar where relevant, unique accession number if available, and source (including location for collected wild specimens).	Not Applicable	
**Microbes:** provide species and strain, unique accession number if available, and source.	Not Applicable	

**Human research participants**	**Information included in the manuscript?**	**In which section is the information available?** (Reagents and Tools Table, Materials and Methods, Figures, Data Availability Section)
If collected and within the bounds of privacy constraints report on age, sex and gender or ethnicity for all study participants.	Not Applicable	

**Core facilities**	**Information included in the manuscript?**	**In which section is the information available?** (Reagents and Tools Table, Materials and Methods, Figures, Data Availability Section)
If your work benefited from core facilities, was their service mentioned in the acknowledgments section?	Yes	Materials and Methods

Design

**Study protocol**	**Information included in the manuscript?**	**In which section is the information available?** (Reagents and Tools Table, Materials and Methods, Figures, Data Availability Section)

If study protocol has been **pre-registered, provide DOI in the manuscript**. For clinical trials, provide the trial registration number **OR** cite DOI.	Not Applicable	
Report the **clinical trial registration number** (at ClinicalTrials.gov or equivalent), where applicable.	Not Applicable	

Laboratory protocol	Information included in the manuscript?	In which section is the information available? (Reagents and Tools Table, Materials and Methods, Figures, Data Availability Section)
Provide DOI OR other citation details if **external detailed step-by-step protocols** are available.	Not Applicable	

Experimental study design and statistics	Information included in the manuscript?	In which section is the information available? (Reagents and Tools Table, Materials and Methods, Figures, Data Availability Section)
Include a statement about **sample size** estimate even if no statistical methods were used.	Yes	Materials and Methods, Figures
Were any steps taken to minimize the effects of subjective bias when allocating animals/samples to treatment (e.g. **randomization procedure**)? If yes, have they been described?	Yes	Materials and Methods
Include a statement about **blinding** even if no blinding was done.	Yes	Materials and Methods
Describe **inclusion/exclusion criteria** if samples or animals were excluded from the analysis. Were the criteria pre-established?<br><br>If sample or data points were omitted from analysis, report if this was due to attrition or intentional exclusion and provide justification.	Yes	Materials and Methods
For every figure, are **statistical tests** justified as appropriate? Do the data meet the assumptions of the tests (e.g., normal distribution)? Describe any methods used to assess it. Is there an estimate of variation within each group of data? Is the variance similar between the groups that are being statistically compared?	Yes	Materials and Methods, Figures

Sample definition and in-laboratory replication	Information included in the manuscript?	In which section is the information available? (Reagents and Tools Table, Materials and Methods, Figures, Data Availability Section)
In the figure legends: state number of times the experiment was **replicated** in laboratory.	Yes	Figures
In the figure legends: define whether data describe **technical or biological replicates**.	Yes	Figures

Ethics

Ethics	Information included in the manuscript?	In which section is the information available? (Reagents and Tools Table, Materials and Methods, Figures, Data Availability Section)
Studies involving **human participants**: State details of **authority granting ethics approval** (IRB or equivalent committee(s), provide reference number for approval.	Not Applicable	
Studies involving **human participants**: Include a statement confirming that **informed consent** was obtained from all subjects and that the experiments conformed to the principles set out in the WMA Declaration of Helsinki and the Department of Health and Human Services Belmont Report.	Not Applicable	
Studies involving **human participants:** For publication of **patient photos**, include a statement confirming that consent to publish was obtained.	Not Applicable	
Studies involving experimental **animals**: State details of **authority granting ethics approval** (IRB or equivalent committee(s), provide reference number for approval. Include a statement of compliance with ethical regulations.	Yes	Materials and Methods
Studies involving **specimen and field samples:** State if relevant **permits** obtained, provide details of authority approving study; if none were required, explain why.	Not Applicable	

Dual Use Research of Concern (DURC)	Information included in the manuscript?	In which section is the information available? (Reagents and Tools Table, Materials and Methods, Figures, Data Availability Section)
Could your study fall under dual use research restrictions? Please check biosecurity documents and list of **select agents and toxins** (CDC): https://www.selectagents.gov/sat/list.htm	Not Applicable	
If you used a select agent, is the security level of the lab appropriate and reported in the manuscript?	Not Applicable	
If a study is subject to dual use research of concern regulations, is the name of the **authority granting approval and reference number** for the regulatory approval provided in the manuscript?	Not Applicable	

Reporting

The MDAR framework recommends adoption of discipline-specific guidelines, established and endorsed through community initiatives. Journals have their own policy about requiring specific guidelines and recommendations to complement MDAR.

Adherence to community standards	Information included in the manuscript?	In which section is the information available? (Reagents and Tools Table, Materials and Methods, Figures, Data Availability Section)
State if relevant guidelines or checklists (e.g., **ICMJE, MIBBI, ARRIVE, PRISMA**) have been followed or provided.	Yes	Materials and Methods
For **tumor marker prognostic studies,** we recommend that you follow the **REMARK** reporting guidelines (see link list at top right). See author guidelines, under 'Reporting Guidelines'. Please confirm you have followed these guidelines.	Not Applicable	
For **phase II and III randomized controlled trials**, please refer to the **CONSORT** flow diagram (see link list at top right) and submit the CONSORT checklist (see link list at top right) with your submission. See author guidelines, under 'Reporting Guidelines'. Please confirm you have submitted this list.	Not Applicable	

Data Availability

Data availability	Information included in the manuscript?	In which section is the information available? (Reagents and Tools Table, Materials and Methods, Figures, Data Availability Section)
Have **primary datasets** been deposited according to the journal's guidelines (see 'Data Deposition' section) and the respective accession numbers provided in the Data Availability Section?	Yes	Data availablility Section
Were **human clinical and genomic datasets** deposited in a public access-controlled repository in accordance to ethical obligations to the patients and to the applicable consent agreement?	Not Applicable	
Are **computational models** that are central and integral to a study available without restrictions in a machine-readable form? Were the relevant accession numbers or links provided?	Not Applicable	
If publicly available data were reused, provide the respective **data citations in the reference list**.	Not Applicable	
